# Soft truncation thresholding for gene set analysis of RNA-seq data: Application to a vaccine study

**DOI:** 10.1038/srep02898

**Published:** 2013-10-09

**Authors:** Brooke L. Fridley, Gregory D. Jenkins, Diane E. Grill, Richard B. Kennedy, Gregory A. Poland, Ann L. Oberg

**Affiliations:** 1 Department of Biostatistics, University of Kansas Medical Center, Kansas City, KS, 66160; 2 Division of Biomedical Statistics and Informatics, Department of Health Sciences Research, Rochester, MN 55905; 3 Department of Molecular Medicine, Mayo Clinic College of Medicine, Rochester, MN 55905; 4 Mayo Vaccine Research Group, Mayo Clinic, Rochester MN, USA; 5 Program in Translational Immunovirology and Biodefense, Mayo Clinic, Rochester MN, USA

## Abstract

Gene set analysis (GSA) has been used for analysis of microarray data to aid the interpretation and to increase statistical power. With the advent of next-generation sequencing, the use of GSA is even more relevant, as studies are often conducted on a small number of samples. We propose the use of soft truncation thresholding and the Gamma Method (GM) to determine significant gene set (GS), where a generalized linear model is used to assess per-gene significance. The approach was compared to other methods using an extensive simulation study and RNA-seq data from smallpox vaccine study. The GM was found to outperform other proposed methods. Application of the GM to the smallpox vaccine study found the GSs to be moderately associated with response, including focal adhesion (p = 0.04) and extracellular matrix receptor interaction (p = 0.05). The application of GSA to RNA-seq data will provide new insights into the genomic basis of complex traits.

With the advent of next-generation sequencing, many researchers are using RNA-Seq to profile mRNA expression of the entire transcriptome. The use of RNA-Seq allows researchers to determine: all transcripts (novel and known); different isoforms; allelic expression; splicing patterns; fusion genes; and differences in expression levels of each transcript^1^. However, due to the relatively high cost of RNA-Seq, many experiments are done with relatively few samples, thus limiting the statistical power to detect differences in gene expression under different conditions. In addition to the limited sample size, the number of features measured on each subject has increased from approximately one million to 10–15 million features (including SNVs, indels, and structural variants) for each subject (depending on the depth of coverage and region targeted for sequencing^2^,^3^) which increases the multiple testing penalty.

As many complex disorders may be controlled by the interplay of multiple genes within the same molecular pathway or gene set (GS), gene set analysis (GSA) has been widely used for mRNA data from microarrays to aggregate the association signals for a set of genes within a GS. This incorporates biological knowledge, reduces the multiple-testing burden, and may increase the association signal, thus increasing the power to detect meaningful associations. With the advent of next-generation sequencing technologies, the use of GSA is even more relevant due to limited statistical power resulting from generally small sample sizes. Over the past ten years, many approaches have been proposed for GSA of mRNA data from microarrays^4^,^5^,^6^,^7^,^8^. In addition, many of the gene set methods proposed for mRNA data have been extended for use in genome-wide genetic association studies using single nucleotide polymorphism (SNP) microarrays^9^,^10^,^11^.

Previous research of self-contained GSA methods (i.e., approaches that test the null hypothesis *H_o_*: *genes within the GS of interest are not associated with the phenotype* versus the alternative hypothesis *H_a_*: *genes within the GS are associated with the phenotype*) found the global random effect model^12^ and Fisher's method to be two powerful approaches for analysis of microarray based mRNA expression data^13^. In addition, we found the soft truncation thresholding Gamma method (GM)^14^, a generalization of Fisher's method for combining p-values, to be more powerful than Fisher's method and the global random effects model for GSA of SNP data^15^. Thus, we hypothesized that the GM would be a powerful approach for GSA of mRNA gene expression data measured by microarray or next generation sequencing technologies to determine sets of genes associated with an endpoint (e.g. differential expressed genes between two experimental conditions). In this manuscript, we present the findings from an extensive simulation study which compares performance of the GM to that of other commonly used self-contained GSA methods on gene expression data. We also applied the GM for GSA to a smallpox vaccine immunogenetic study involving RNA-Seq data to determine GSs with differentially expressed genes between high and low responders to the vaccine.

## Results

### Simulation studies

**Figure 1:**
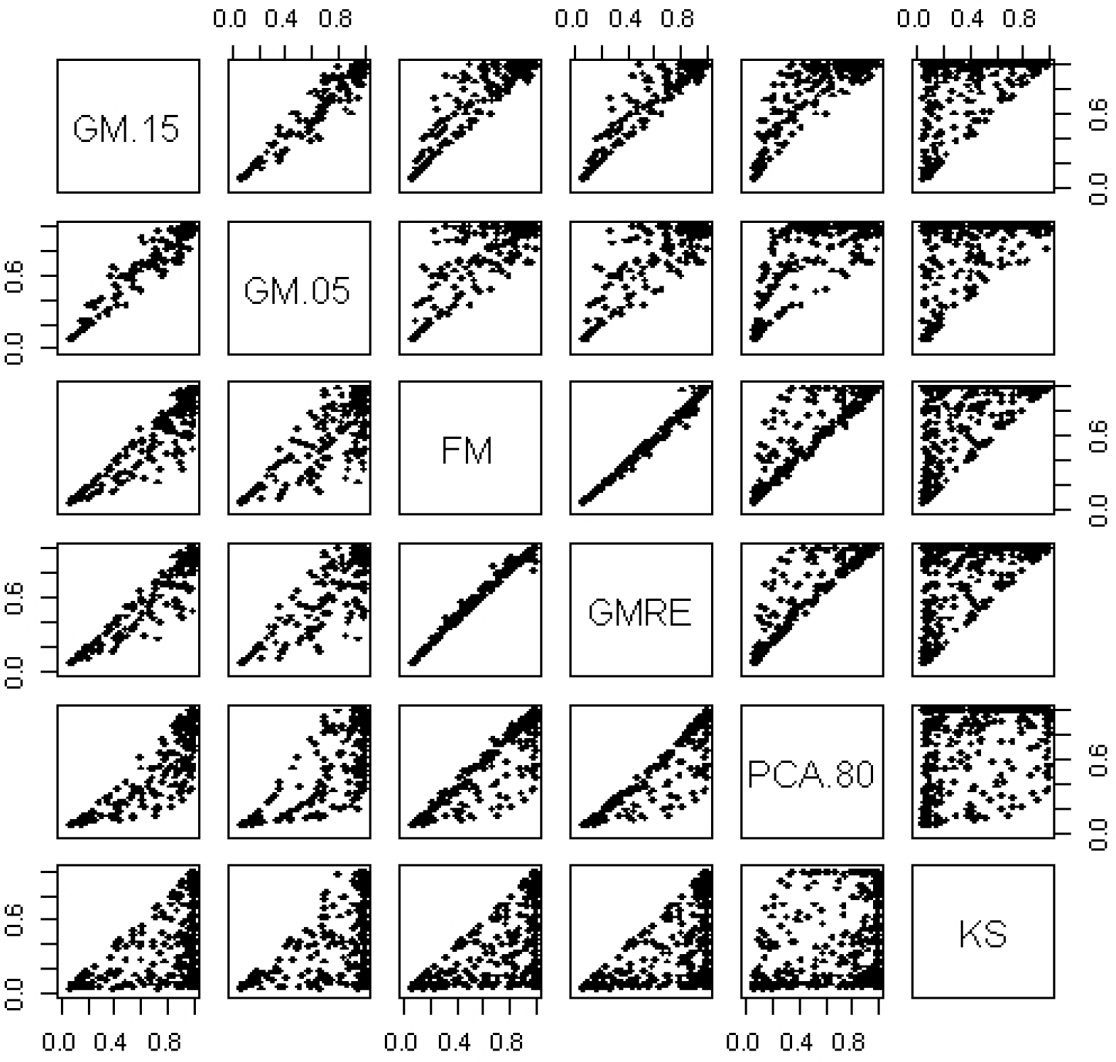
Power comparison between the Gamma Method with STT = 0.15 and 0.05 (GM.15, GM.05), Fisher's Method (FM), Global model with random effects (GMRE), Principal components analysis with 80% of components that explained the variability included in the model (PCA.80), Kolmogorov-Smirnov test (KS).

The results from the simulation studies found the GM to outperform other proposed approaches for self-contained GSA. All methods had adequate control of the type I error rate. Table 1 presents the summary of the power for the various methods across all simulation scenarios and Figure 1 presents a power comparison between simulation scenarios for the subset of the most powerful GSA methods. The full set of results for all methods assessed can be found in Supplemental Table 1. The GM with soft truncation threshold (STT) < 1/e consistently outperformed the other commonly used GSA approaches for gene expression data. The only method that had similar performance was the full model with fixed effects; however, this approach could only be applied to approximately two-thirds of the simulation scenarios where the number of genes in the GS was less than the number of samples.

**Figure 2:**
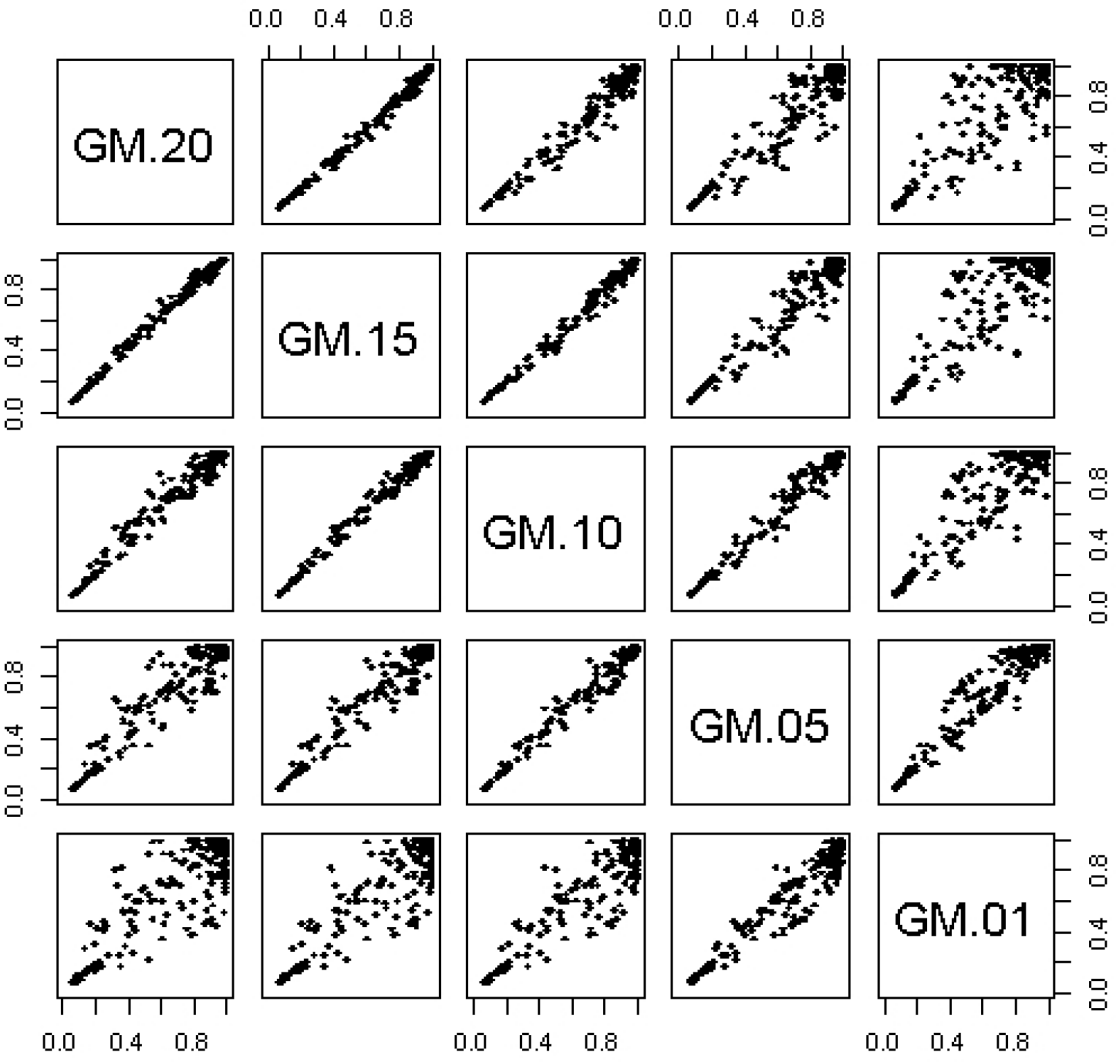
Power comparison between the Gamma Method with various STT values. STT values ranged from 0.20 to 0.01.

Comparison of the GM with various STT values, ranging from 0.20 to 0.01, is presented in Figure 2. As expected, there was more similarity between the GM with similar STT values (e.g., GM with STT = 0.20 versus GM with STT = 0.15) and less similarity between results when the STT values were further apart (e.g., GM with STT = 0.20 versus GM with STT = 0.01). The optimal value of the STT for a given GSA will depend on the underlying, unknown, disease model, but in general STT values that are not too small, e.g. between 0.01 and 1/e (≈0.36), tend to give the best power. This coincides with the rationale for GSA, in that we wish to detect GSs with multiple genes with moderate or small effects for which we have limited power to detect individually.

Finally, based on results from the null simulations designed from the smallpox vaccine study, no relationship was found between type I error rate and: number of genes in a GS; average length of genes in a GS; sum of all lengths of genes in a GS; and the number of "large" genes in a GS (Supplemental Figure 1). We also observed that the GM's type I error rate was found to be controlled for all values of STT (and thus the corresponding shape parameter *ω* in the Gamma transformation).

### Smallpox vaccine study

The GM with various STT values, including FM, was applied to the smallpox vaccine study to determine if any GSs in KEGG^16^ were associated with response to the smallpox vaccine. Table 2 presents GSs with p < 0.05 for any of the GM analyses with various STT values. To adjust for multiple testing, FDR q-values were computed^17^. For the analysis of response to smallpox vaccine, there was little difference between the GM results with STT < 0.20, while the largest p-values resulted from FM (i.e., GM with STT = 1/e). The top associated GSs (with pathway coverage ≥ 70%) included: Biotin metabolism (p = 0.0005, q = 0.02); non-homologous end-joining (p = 0.02, q = 0.17); focal adhesion (p = 0.04, q = 0.17); D-Glutamine and D-glutamate metabolism (p = 0.04, q = 0.17); and ECM-receptor interaction (p = 0.05, q = 0.17).

**Figure 3:**
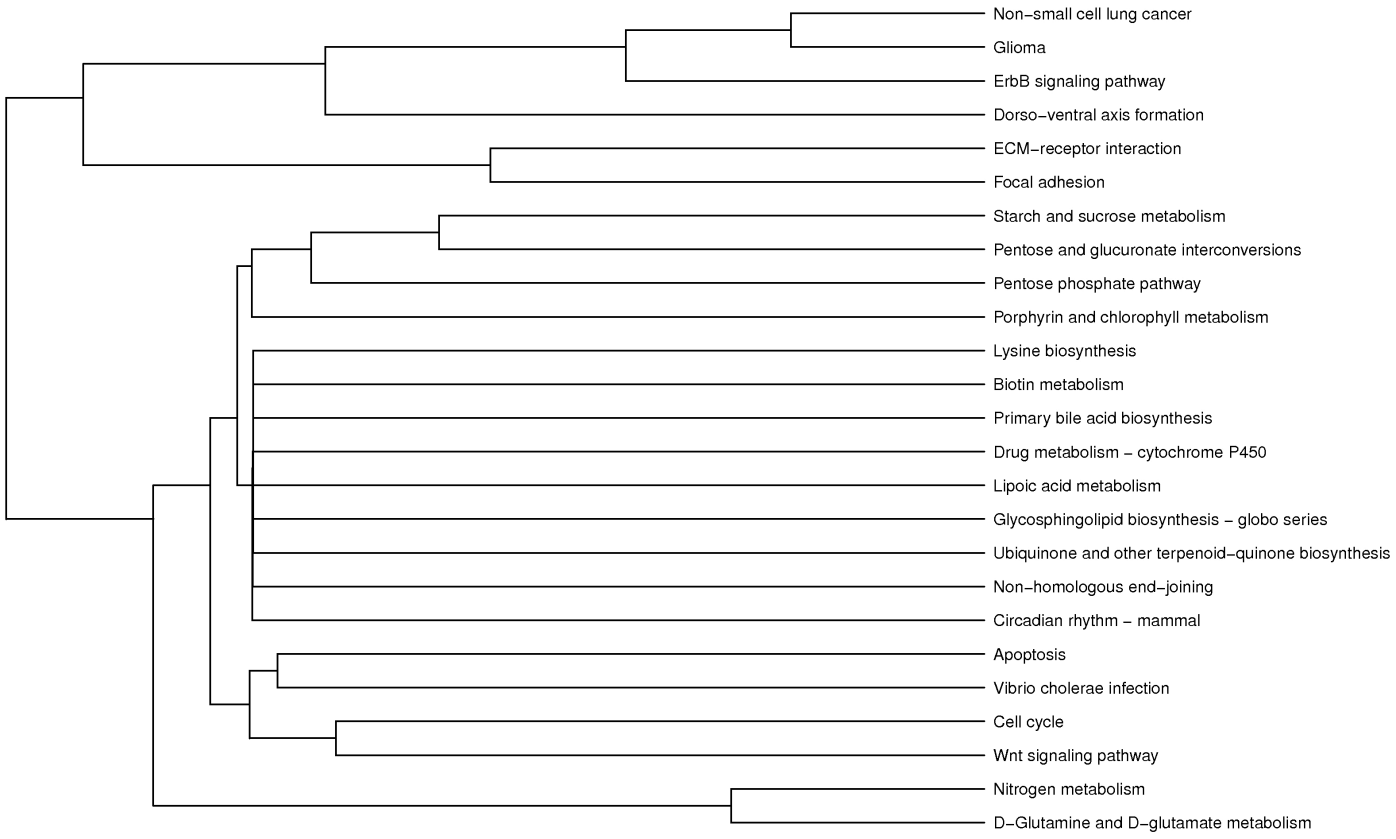
Dendrogram of top 25 GS associated with response to smallpox vaccine to visualize relationship and overlap between gene sets. GSs containing a large set of genes in common would be clustered close together while GSs with no genes in common would not be clustered together.

Within the top GSs, Table 3 presents the genes with gene-level association p < 0.15. Of particular relevance to vaccine response are the genes within the focal adhesion GS (p < 0.15 for 30 genes out of the 148 genes measured in pathway of 201 genes), as many of these genes are involved in cytokine-cytokine receptor interactions. Another interesting GS is the EMC-receptor interaction GS (p < 0.15 for 9 genes out of the 59 genes measured in the pathway of 84 genes). Genes within this GS interact with a number of immunologically important cell surface molecules including: integrins which mediate cytokine adhesion, extravasation, and homing; *CD44* (a cell surface glycoprotein involved in lymphocyte homing, migration, and activation); and *CD36* (a scavenger receptor expressed on multiple cell types including: monocytes, macrophage, dendritic cells). *SPP1* (osteopontin), the top-most associated gene in the ECM-receptor interaction GS, up-regulates the production of IFNγ and IL-12 thereby driving Th1-type immune responses. The *MAPK9* gene identified in the focal adhesion GS is a member of the MAP kinase family and is required for differentiation of T helper cells into Th1 cells. As depicted in the dendrogram in Figure 3, these two GSs contain many genes in common, including genes *COL1A2* (p = 0.0278), *THBS4* (p = 0.0311) and *ITGB3* (p = 0.0168).

## Discussion

In this paper, we present the use of the GM for GSA to determine GSs in which the transcript levels for genes within the GS are associated with a phenotype. The method is able to be used in both the context of microarray data and next-generation sequence data, along with the ability to be used for both binary and quantitative traits. An extensive simulation study, involving over 1,400 simulation scenarios, was completed to compare the GM with various levels of STT to other GSA methods, including Fisher's method and the Global model with random effects of Goeman et al. (2004), that were found to be powerful approaches for self-contained GSA in past research^13^. From our simulation study we found the GM with STT < 0.20 to uniformly outperform the other methods, while maintaining type I error rate control.

In addition to the simulation study, we applied the GM for GSA to a RNA-Seq smallpox study to identify GSs with differences in mRNA expression between high and low responders to the smallpox vaccine. The top biologically relevant GSs included focal adhesion (p = 0.04) and ECM-receptor interaction (p = 0.05). Of note, these GSs mediate communication and interactions between the leukocytes involved in immune responses. It is possible that the differential expression seen in the high and low responders reflects a differential ability of circulatory peripheral blood mononuclear cells to recognize viral infection and coordinate the resulting immune responses. These results indicate that further examination of these gene pathways in mixed cell populations and in specific cell subsets (i.e. B cells, monocytes, CD4 T cells) is warranted, as such studies may further our understanding of poxvirus immunity.

In conclusion, this research shows the GM with STT < 0.20 to be a powerful method for GSA. In practice, we suggest the selection of an STT value between 0.10 and 0.20, realizing the optimal STT depends on each individual study. Care should be taken in the interpretation of results from GSA completed based on multiple STT values (i.e., multiple testing and “data snooping”). In addition, the application of the method to a smallpox vaccine study has provided new insights into the genomic basis of individual variations in immune response to the vaccine.

## Methods

### Vaccine study

In brief, 21 high and 23 low responders to smallpox vaccine based on extremes of antibody titer were selected from a cohort of 1076 successful smallpox vaccine recipients. Aliquots of these 44 subjects' peripheral blood mononuclear cells (PBMCs) were either left unstimulated or were stimulated with vaccinia virus for a total of 88 specimens. Specimens were allocated to flow cell and lane for sequencing ensuring that response status was balanced over these potential experimental effects, ensuring the paired specimens from a given subject were in adjacent lanes on the same flow cell. For purposes of the GSA methods comparison here, we focus on the 44 stimulated specimens to test the hypothesis of differential gene expression between high and low responders. Full details of the study cohort are provided in Kennedy et al. (2009), Haralambieva et al.(2011), Ovsyannikova et al. (2011)^18^,^19^,^20^,^21^, and of the PBMC stimulation and RNA-Seq methods in Kennedy et al (2013)^22^.

### Simulation study

#### Power and type I error simulations

The simulation of gene expression data and a quantitative phenotype was completed in a similar manner as outlined in Fridley et al^13^. Briefly, let *n* represent the number of subjects and *m* represent the number of genes in a GS. The expression data for each subject (*i* = 1,…,*n*) was simulated from a multivariate normal distribution with mean equal to zero and covariance matrix Σ. The matrix Σ was set to either the case where there is no correlation between the genes in a GS or a structure in which all genes in the GS are correlated the same amount. While these precise correlation structures are not biologically realistic, they do facilitate the comparison of the algorithms, and the relative performance of the algorithms should extend to correlation structures observed in real data. The quantitative phenotype (*Y*_i_) for each subject was generated conditional on the gene expression data, *Y_i_* ~ N(*μ_i_*, *σ*^2^) with *μ_i_* = **β***^T^***X**_i_, where ***X****_i_* represents the vector of gene expression values for genes within the GS for subject *i* and **β** is the vector of gene level effects. The simulation scenarios varied in terms of the number and size of the effects (**β**), sample size, GS size, correlation between genes within a GS, and variation in the phenotype. For each of the simulation scenarios, 1000 data sets were generated to assess either the power (1440 scenarios) or the type I error rate (72 scenarios). For determining power and type I error rate, the significance level was set to 0.05.

#### Null simulations based on smallpox vaccine study

In addition to the power and type I error rate estimates based on the simulated data, we also investigated the possible impact of gene size and gene set size on the type I error rate in GSA of RNA-Seq data. Using the stimulated group of samples in the smallpox study, we permuted the high (H) and low (L) responder status 100 times to generate 100 “null” data sets with no association (beyond that of chance) between response and level of gene expression. In doing so, we keep the structure of the RNA-Seq data intact to preserve the correlation structure between the gene expression levels. For each null data set, we then completed GSA with the GM for various soft truncation threshold (STT) values for the 200 KEGG GSs. In completing the gene level association analyses, the same gene-specific dispersion estimate was used for all 100 data sets. We then compared the type I error rates between the various sizes of gene sets (number of genes), average gene length in the GS, sum of all the gene lengths within a GS, and number of genes in a gene set with gene size larger than the 75% of all gene lengths.

### Gene set analysis method for RNA-Seq data

#### Gene-level assessment

Prior to the completion of the self-contained GSA with the GM, the gene-level association p-values must be determined. In contrast to microarray based mRNA data in which relative mRNA expression is measured for pre-defined probe sets via fluorescence, RNA-Seq experiments measure the gene expression levels from the total number of reads that fall into the exons of a gene. To assess the significance of each gene with the outcome (i.e., differential gene expression analysis), we used a generalized linear model that assumed a Negative Binomial distribution^23^. The Negative Binomial distribution is appropriate for count data where within-subject technical variation follows a Poisson distribution with subject-specific mean λ, and the between-subject biological variation of λ follows a Gamma distribution. In addition, we assumed that the nature of over-dispersion differs across genes. Thus, an Empirical Bayes-like moderated test implemented in the R package *edgeR* was used, in which gene specific dispersion parameters were estimated with “shrinkage” of estimates to the overall mean using a quantile-adjusted conditional maximum likelihood method, scaled by the 75%-tile^24^,^25^,^26^. Genes were removed from analysis if they had low coverage (i.e., average count ≤5).

#### Gene set assessment

The GSA is then completed with the application of the GM to the gene-level association p-values produced from the negative binomial testing framework. The GM is based on summing p-values transformed using an inverse Gamma (ω, 1) transformation. For a particular shape parameter *ω*, the test statistic is defined as 
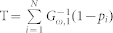
, where *G*^−1^ is the inverse of a Gamma (ω, 1) cumulative distribution function^14^. The varying of *ω* results in different transformations applied to the p-values, resulting in more emphasis being given to p-values below a particular threshold, referred to as the STT. The STT is controlled by the shape parameter *ω*, where 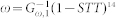
14. When *ω* is 1, the GM becomes equivalent to Fisher's method (FM) with a STT value of 1/e.

Non-independence of gene-specific p-values due to correlation of expression of genes in a gene set can lead to departures of p-values from the expected Uniform(0,1) distribution under the null hypothesis. Due to this lack of independence between the gene-level p-values, we utilized Monto Carlo estimation of the test-statistic T's null distribution to compute empirical or “non-parametric” based p-values for each GS^15^,^27^. In doing so, the phenotypic variable is randomly permuted, preserving the correlation structure in the RNA-seq gene expression count data. Using the permuted data, differential expression analysis for each gene was then computed using *edgeR*, followed by the determination of the GSA T test statistic. This process was repeated many times (e.g. 1,000 times), producing an empirical distribution of the test statistic T. The proportion of permutations in which the test statistic was smaller than the observed data test statistic was the empirical estimate of the GS p-value. To visualize the overlap of the genes within the various GSs, hierarchical clustering was completed using a distance measure between GSs defined as 1 − τ, where τ represents the average proportion of genes shared between the GSs.

For the smallpox vaccine study and the simulation studies, GSA was completed using the GM with six different STTs ranging from 0.01 to 1.0 (i.e., Fisher's method). We also compared the results from the GM to methods assessed in Fridley et al (2010)^13^: global model with fixed effects (GMFE); global model with random effects (GMRE)^12^; tail strength (TS)^28^; Kolmogorov-Smirnov (KS); and a principal component approach with either the top *k* principal components needed to explain 80% (PCA80) of the variation in the gene expression values within the gene set, the top principal component (PCA1) or top five principal components (PCA1.5).

## Author Contributions

B.L.F. conceived of the study, made the figures and tables, and wrote the majority of the manuscript text. G.D.J. and D.E.G. completed all statistical analyses outlined in the manuscript. R.B.K., A.L.O., D.E.G. and G.A.P. completed the sequencing study for the smallpox vaccine immunogenetics study. All authors reviewed and edited the manuscript text.

## Supplementary Material

Supplementary Information Supplemental Figure 1 and Table 1

## Figures and Tables

**Table 1:** Summary of power across all 1440 non-null simulation scenarios for sample sizes of N = 500 and N = 100, with 1000 simulated data sets per scenario. The GM with various STT values is compared to ten previously proposed self-contained GSA methods. Table entries are sorted by descending mean power for the scenarios with sample size of 500

		N = 500				N = 100			
Method	STT	Min.	1st Qu.	Median	Mean	Min.	1st Qu.	Median	Mean
Gamma Method (GM)	0.1	0.264	1	1	0.991	0.072	0.994	1	0.923
GM	0.05	0.331	1	1	0.993	0.073	0.990	1	0.921
GM	0.15	0.210	1	1	0.990	0.073	0.995	1	0.920
Global model with fixed effects (GMFE)*		0.223	1	1	0.985	0.068	0.998	1	0.906
GM	0.2	0.171	1	1	0.988	0.068	0.992	1	0.916
GM	0.01	0.449	1	1	0.993	0.067	0.96	1	0.903
Global model using random effects (GMRE)		0.111	1	1	0.983	0.065	0.945	1	0.896
Fisher's Method/Gamma Method (FM)	1/e	0.096	1	1	0.980	0.059	0.943	1	0.889
PCA using principal components that explain 80% of variation (PCA80)		0.101	1	1	0.974	0.06	0.856	1	0.855
Stouffer's Method (SM)		0.062	1	1	0.933	0.051	0.674	1	0.816
FTS. GS Modified Tail Strength (MTS)		0.057	1	1	0.924	0.045	0.574	1	0.787
PCA using top five principal components (PCA1.5)		0.07	0.958	1	0.920	0.056	0.631	0.993	0.788
Tail Strength (TS)		0.078	0.981	1	0.867	0.059	0.621	1	0.797
Kolmogorov-Smirnov (KS)		0.051	0.960	1	0.819	0.047	0.394	0.999	0.738
PCA using top principal component (PCA1)		0.056	0.591	1	0.808	0.051	0.314	0.991	0.704

*198 and 396 scenarios were unable to be fit do to size of gene set for N = 500 and N = 100, respectively.

**Table 2:** Top GSs associated with response to Smallpox vaccine for various STT values. Results with p < 0.05 from GSA using the GM with any of the STT values are presented

	GSA P-values for various STT Value							
Gene Set	0.05	0.10	0.15	0.20	1/e	N Genes in KEGG	N Genes in Analysis	Coverage of Pathway
Biotin metabolism	0.0005*	0.0005	0.0005	0.0005	0.002	2	2	100%
Pentose and glucuronate interconversions	0.018	0.021	0.025	0.031	0.119	28	8	29%
Non-homologous end-joining	0.022	0.026	0.031	0.039	0.121	14	12	86%
Focal adhesion	0.039	0.053	0.065	0.076	0.103	201	148	74%
D-Glutamine and D-glutamate metabolism	0.041	0.041	0.042	0.041	0.050	4	4	100%
ECM-receptor interaction	0.046	0.064	0.080	0.093	0.129	84	59	70%
Lysine biosynthesis	0.058	0.055	0.048	0.042	0.044	4	3	75%

*FDR q-value was 0.02.

**Table 3:** Gene-level results (p < 0.15) for GSs with p < 0.05

Gene Set	Gene	P-value	Gene Set	Gene	P-value
Biotin metabolism	*HLCS*	0.0005	Focal adhesion	*COL1A2*	0.0278
Pentose & glucuronate interconversions	*UGP2*	0.1160		*THBS4*	0.0311
	*DCXR*	0.0115		*AKT3*	0.0385
Non-homologous end-joining	*PRKDC*	0.0164		*SOS2*	0.0430
	*RAD50*	0.0613		*BRAF*	0.0468
	*MRE11A*	0.0802		*PDGFA*	0.0493
D-Glutamine & D-glutamate metabolism	*GLS2*	0.0176		*JUN*	0.0496
	*GLS*	0.1061		*FLNB*	0.0521
ECM-receptor interaction	*SPP1*	0.0117		*ZYX*	0.0626
	*ITGB3*	0.0168		*PIK3CA*	0.0776
	*LAMB1*	0.0208		*PPP1R12A*	0.0788
	*COL1A2*	0.0278		*MAPK8*	0.0801
	*THBS4*	0.0311		*ERBB2*	0.0814
	*COL5A1*	0.0947		*ROCK2*	0.0899
	*COL11A2*	0.1277		*COL5A1*	0.0947
	*COL5A2*	0.1288		*GRLF1*	0.0996
	*HSPG2*	0.1452		*PIK3R1*	0.1028
focal adhesion	*PRKCA*	0.0090		*CCND3*	0.1089
	*SPP1*	0.0117		*SHC1*	0.1170
	*MAPK9*	0.0131		*ARHGAP5*	0.1252
	*IGF1R*	0.0132		*COL11A2*	0.1277
	*ITGB3*	0.0168		*COL5A2*	0.1288
	*LAMB1*	0.0208		*BIRC3*	0.1440
	*VASP*	0.0219			
