# Supplementary material for: Soft truncation thresholding for gene set analysis of RNA-seq data: Application to a vaccine study
Source: Sci Rep. 2013 Oct 9;3:2898. doi: 10.1038/srep02898 (PMC3793215; doi:10.1038/srep02898)
Supplement: Supplementary Information — Supplemental Figure 1 and Table 1 [file srep02898-s1.pdf]

Title: Soft truncation thresholding for gene set analysis of RNA-seq data: Application to a vaccine study

Authors: Brooke L. Fridley, Gregory D. Jenkins, Diane E. Grill, Richard B. Kennedy, Gregory A. Poland, Ann L. Oberg

Supplemental Table 1: Power or Type I error rate for all scenarios for each self-contained gene set method assessed.

Supplemental Figure 1: Results from null simulations based on vaccine study data. No relationship observed between type I error rate and: number of genes in a gene set; average length of genes in a gene set; sum of all lengths of genes in a gene set; and the number of "large" genes in a gene set.

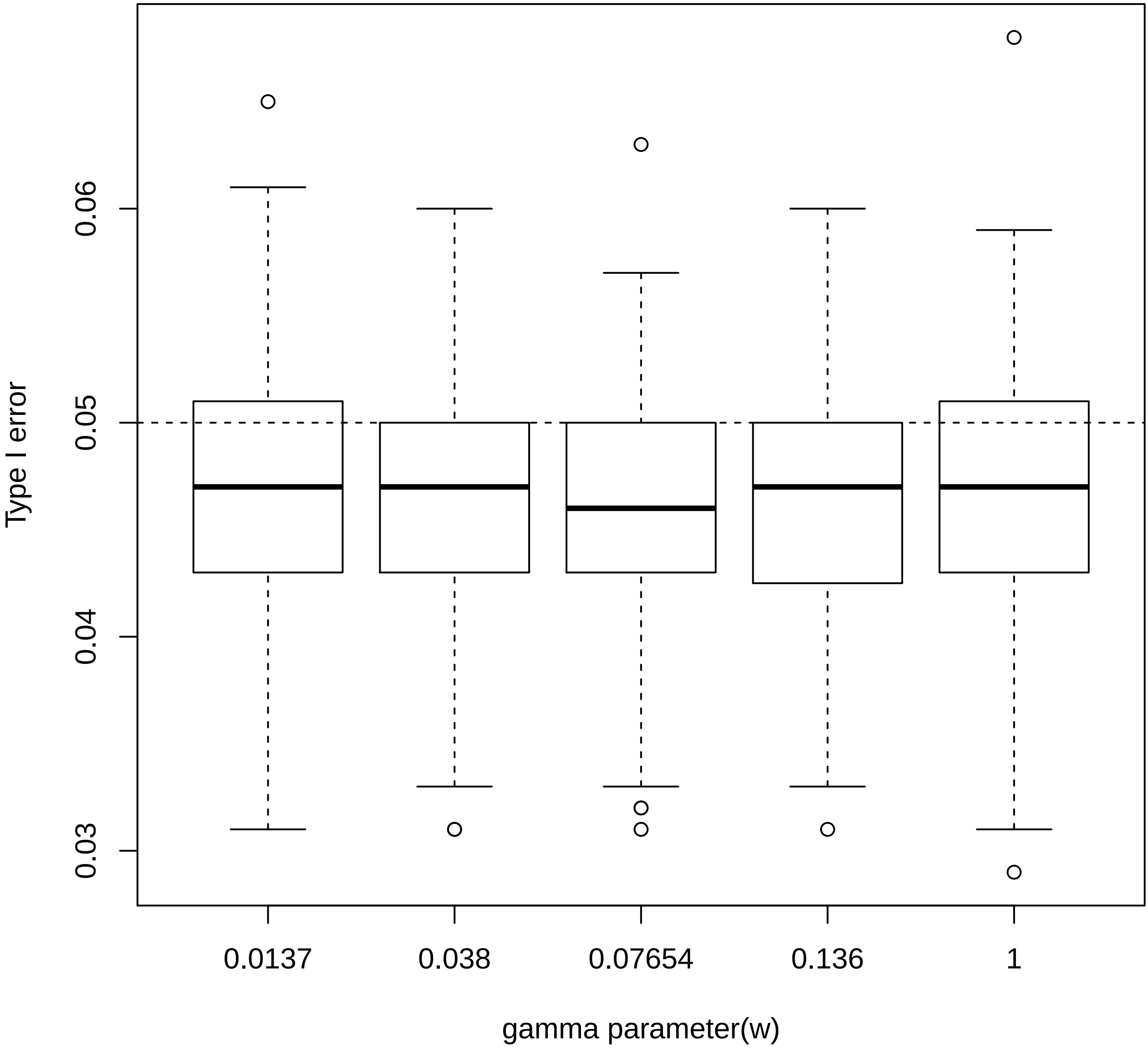

Type I error
0.03
0.04
0.05
0.06
0.0137
0.038
0.07654
0.136
1
gamma parameter(w)

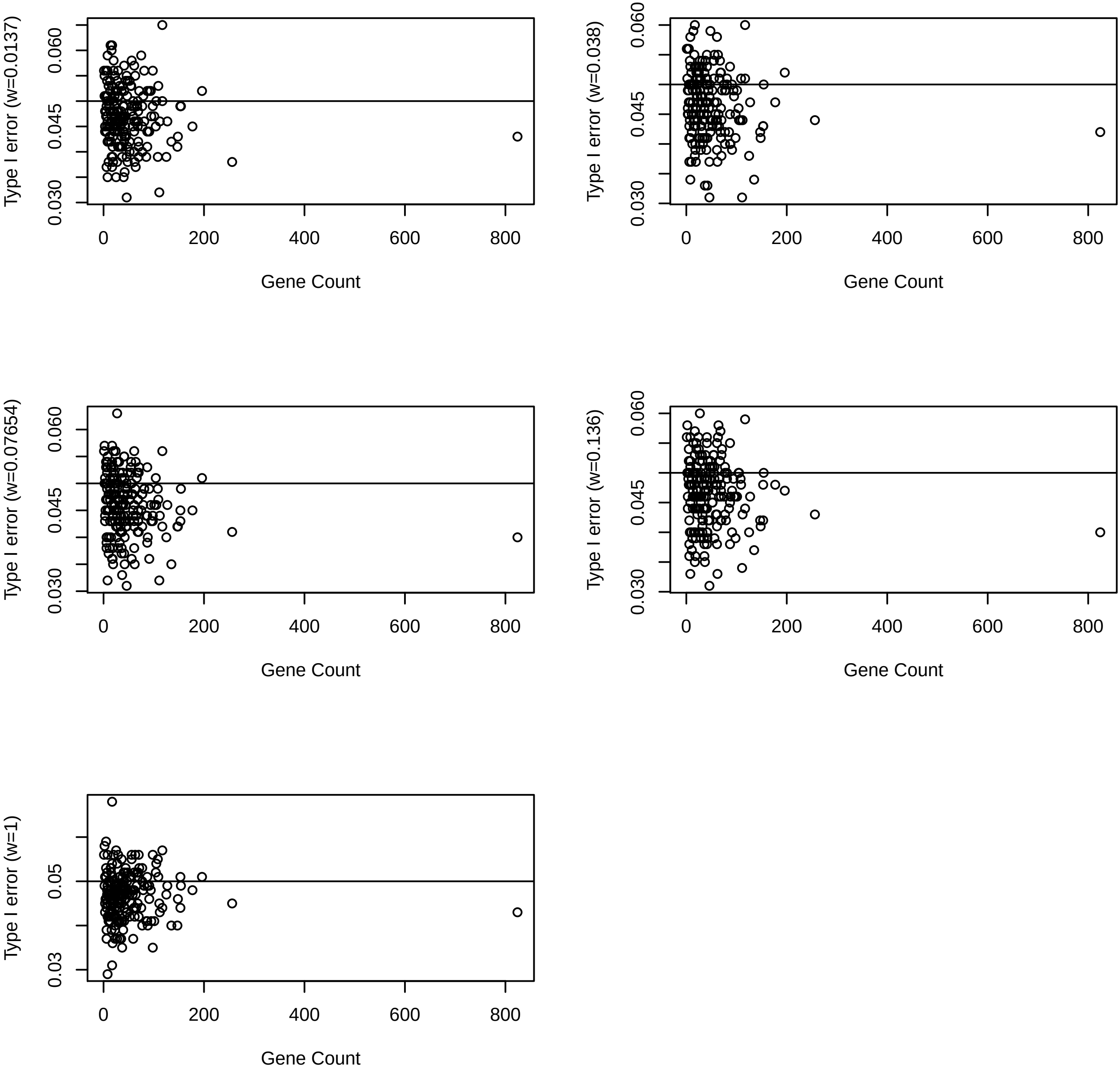
Type I error (w=0.0137)
Type I error (w=0.038)
Type I error (w=0.07654)
Type I error (w=0.136)
Type I error (w=1)
Gene Count
0.030
0.045
0.060
0.03
0.05
0
200
400
600
800

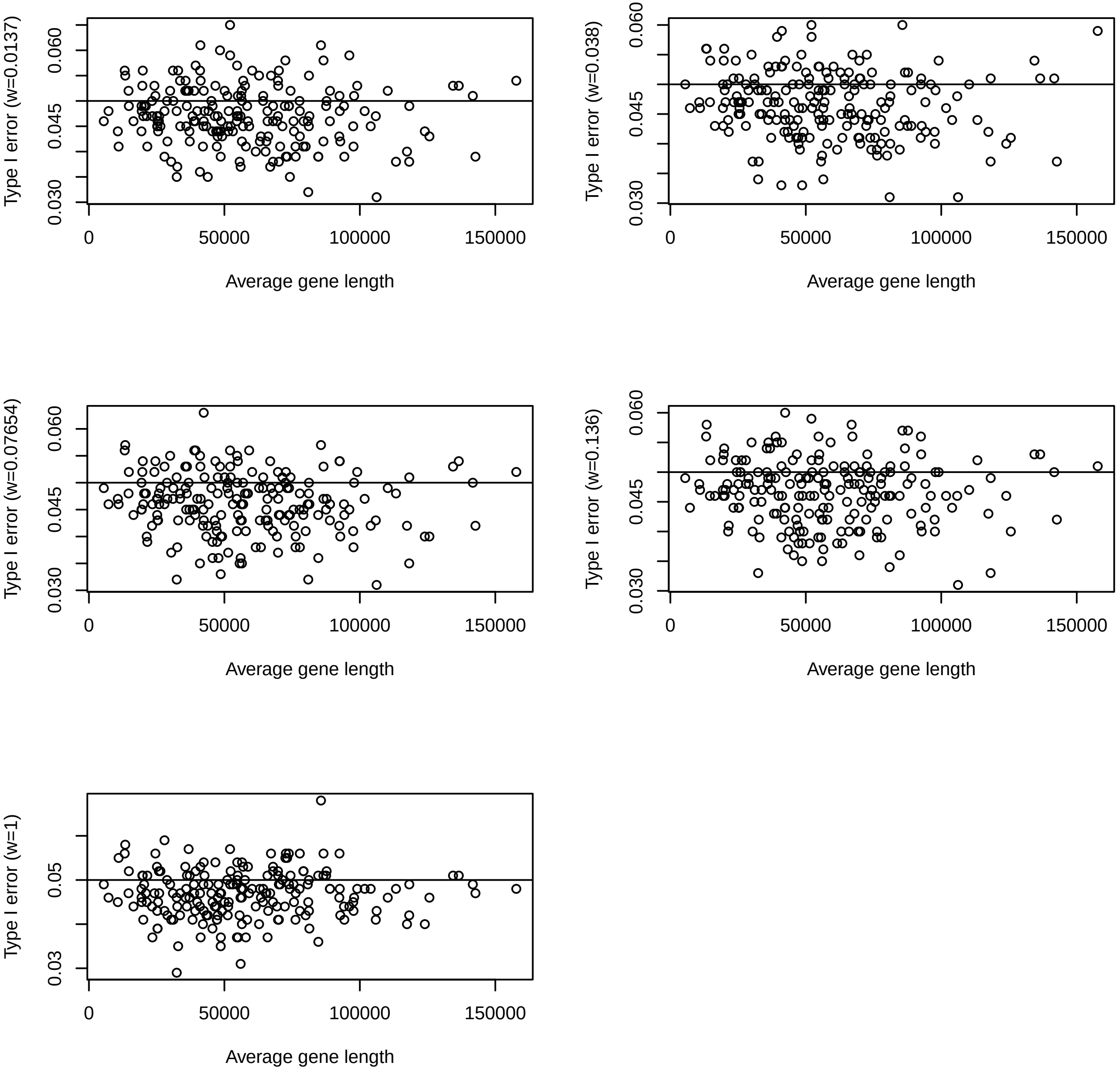
Type I error (w=0.0137)
Type I error (w=0.038)
Type I error (w=0.07654)
Type I error (w=0.136)
Type I error (w=1)
0.030
0.045
0.060
0.03
0.05
0
50000
100000
150000
Average gene length

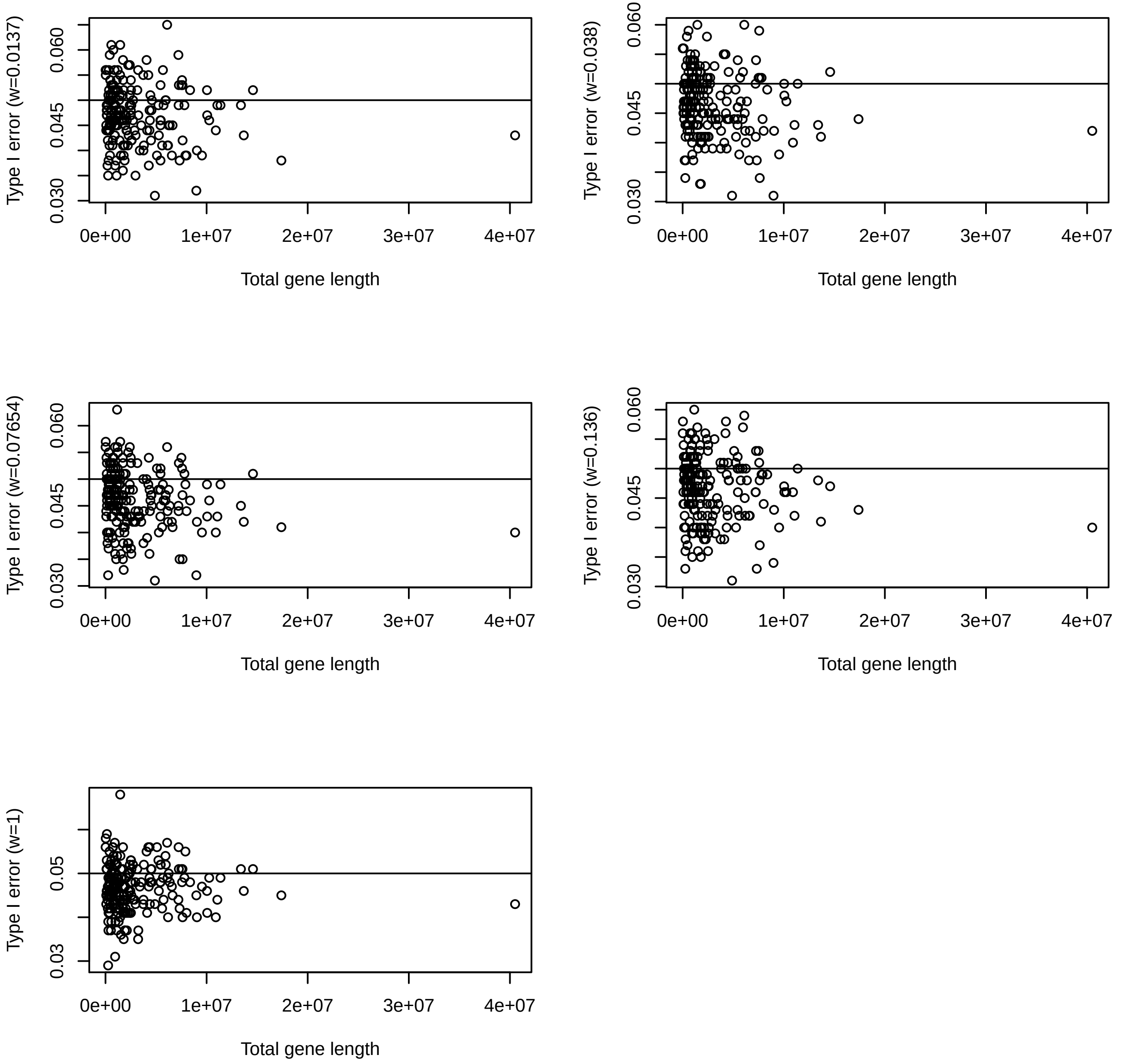
Type I error (w=0.0137)
0.030
0.045
0.060
0e+00
1e+07
2e+07
3e+07
4e+07
Total gene length
Type I error (w=0.038)
0.030
0.045
0.060
0e+00
1e+07
2e+07
3e+07
4e+07
Total gene length
Type I error (w=0.07654)
0.030
0.045
0.060
0e+00
1e+07
2e+07
3e+07
4e+07
Total gene length
Type I error (w=0.136)
0.030
0.045
0.060
0e+00
1e+07
2e+07
3e+07
4e+07
Total gene length
Type I error (w=1)
0.03
0.05
0e+00
1e+07
2e+07
3e+07
4e+07
Total gene length

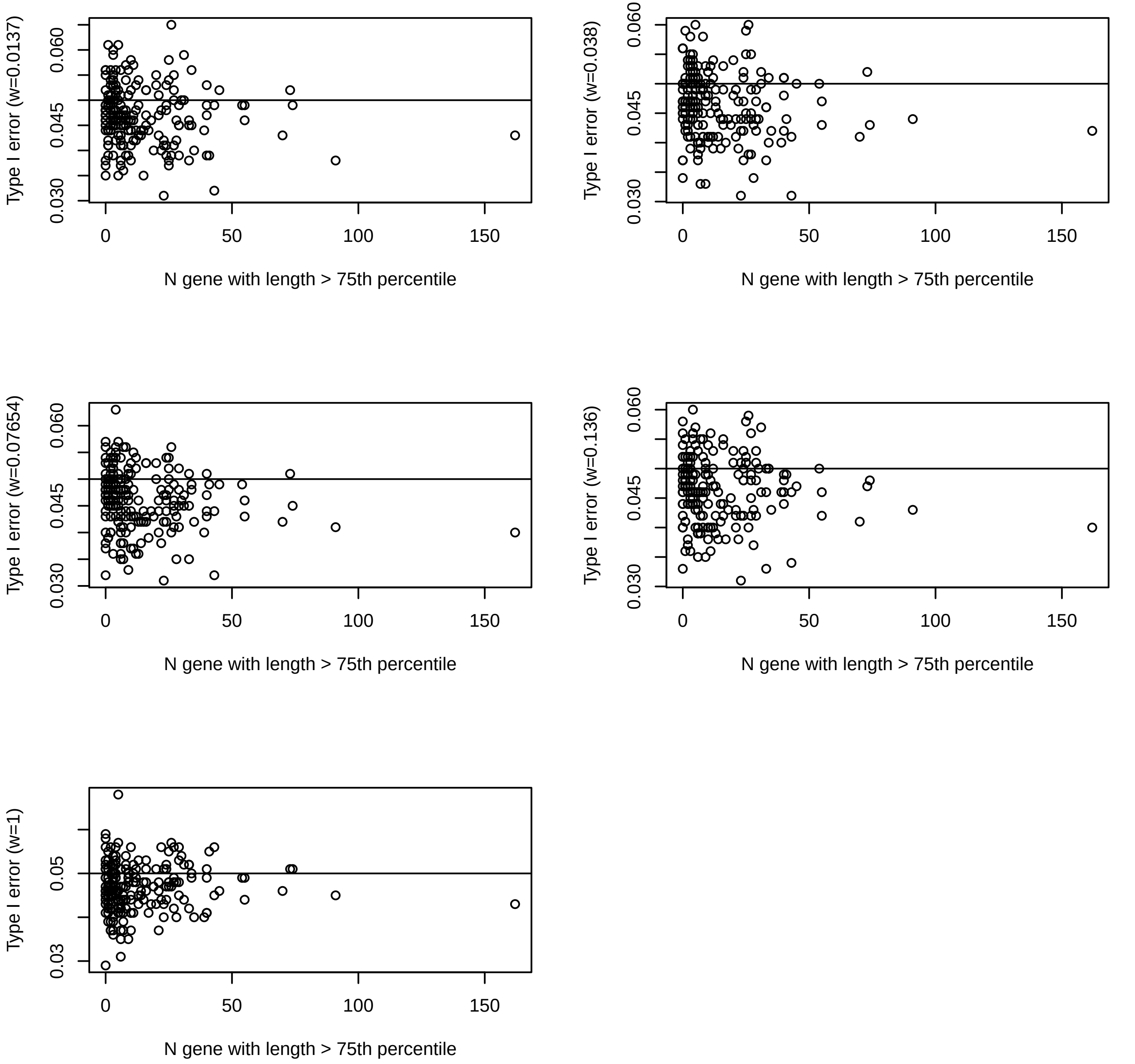
Type I error (w=0.0137)
Type I error (w=0.038)
Type I error (w=0.07654)
Type I error (w=0.136)
Type I error (w=1)
0.030
0.045
0.060
0.03
0.05
0
50
100
150
N gene with length > 75th percentile

Supplemental Table 1: Power or Type I error rate for all scenarios for each method.

				Number of Genes				Self-Contained Gene Set Methods Assessed														
													GM (given shape parameter)									
Power Scenario	N	rho	sigma	GS	Small Effect	Medium Effect	Large Effect	TS	KS	FM	SM	FTS-GS	0.136	0.0765	0.038	0.0137	0.001739	GMRE	PCA.80PCT	PCA1	PCA1.5	GMFE
0	100	0	1	10	0	0	0	0.048	0.047	0.049	0.049	0.048	0.043	0.046	0.051	0.05	0.05	0.045	0.048	0.053	0.036	0.047
0	500	0	1	10	0	0	0	0.049	0.051	0.05	0.053	0.043	0.059	0.057	0.061	0.064	0.061	0.057	0.05	0.048	0.053	0.056
0	100	0.1	1	10	0	0	0	0.038	0.038	0.043	0.041	0.034	0.05	0.049	0.049	0.05	0.052	0.047	0.042	0.048	0.045	0.047
0	500	0.1	1	10	0	0	0	0.058	0.052	0.059	0.05	0.048	0.064	0.062	0.058	0.058	0.055	0.06	0.053	0.055	0.063	0.056
0	100	0.3	1	10	0	0	0	0.048	0.047	0.045	0.049	0.052	0.042	0.042	0.044	0.049	0.047	0.044	0.037	0.041	0.043	0.047
0	500	0.3	1	10	0	0	0	0.051	0.058	0.051	0.051	0.055	0.052	0.056	0.054	0.054	0.048	0.053	0.053	0.052	0.045	0.056
0	100	0	1	50	0	0	0	0.051	0.047	0.053	0.044	0.037	0.042	0.049	0.046	0.056	0.055	0.047	0.047	0.069	0.057	0.043
0	500	0	1	50	0	0	0	0.045	0.05	0.047	0.052	0.041	0.05	0.047	0.048	0.054	0.054	0.048	0.047	0.045	0.046	0.045
0	100	0.1	1	50	0	0	0	0.057	0.057	0.057	0.06	0.057	0.053	0.056	0.059	0.065	0.06	0.053	0.049	0.048	0.052	0.043
0	500	0.1	1	50	0	0	0	0.042	0.051	0.048	0.042	0.042	0.049	0.049	0.044	0.047	0.05	0.05	0.05	0.047	0.043	0.045
0	100	0.3	1	50	0	0	0	0.054	0.055	0.053	0.054	0.056	0.055	0.056	0.056	0.057	0.051	0.054	0.046	0.047	0.046	0.043
0	500	0.3	1	50	0	0	0	0.052	0.05	0.05	0.049	0.048	0.047	0.049	0.047	0.045	0.045	0.048	0.046	0.049	0.048	0.045
0	100	0	1	100	0	0	0	0.047	0.048	0.053	0.047	0.047	0.066	0.06	0.055	0.051	0.051	0.055	0.043	0.049	0.054	NA
0	500	0	1	100	0	0	0	0.056	0.055	0.056	0.05	0.044	0.058	0.056	0.054	0.052	0.043	0.052	0.061	0.039	0.045	0.055
0	100	0.1	1	100	0	0	0	0.047	0.044	0.052	0.049	0.048	0.049	0.05	0.053	0.047	0.055	0.048	0.047	0.046	0.057	NA
0	500	0.1	1	100	0	0	0	0.051	0.055	0.049	0.054	0.051	0.044	0.045	0.052	0.048	0.046	0.049	0.068	0.05	0.048	0.055
0	100	0.3	1	100	0	0	0	0.048	0.048	0.05	0.049	0.049	0.051	0.049	0.051	0.049	0.045	0.052	0.05	0.045	0.055	NA
0	500	0.3	1	100	0	0	0	0.05	0.053	0.05	0.051	0.05	0.049	0.044	0.043	0.045	0.045	0.053	0.051	0.051	0.048	0.055
0	100	0	1	500	0	0	0	0.045	0.046	0.048	0.038	0.037	0.058	0.06	0.064	0.053	0.05	0.052	0.055	0.047	0.049	NA
0	500	0	1	500	0	0	0	0.057	0.046	0.057	0.053	0.049	0.055	0.058	0.061	0.049	0.058	0.062	0.051	0.057	0.051	NA
0	100	0.1	1	500	0	0	0	0.054	0.054	0.052	0.056	0.059	0.052	0.055	0.053	0.053	0.052	0.055	0.059	0.053	0.048	NA
0	500	0.1	1	500	0	0	0	0.05	0.05	0.054	0.046	0.044	0.055	0.055	0.057	0.058	0.063	0.054	0.05	0.049	0.05	NA
0	100	0.3	1	500	0	0	0	0.052	0.055	0.051	0.051	0.053	0.053	0.052	0.054	0.057	0.055	0.053	0.048	0.054	0.052	NA
0	500	0.3	1	500	0	0	0	0.049	0.049	0.049	0.05	0.047	0.05	0.051	0.051	0.052	0.061	0.053	0.053	0.05	0.061	NA
0	100	0	3	10	0	0	0	0.048	0.047	0.049	0.049	0.048	0.043	0.046	0.051	0.05	0.05	0.045	0.048	0.053	0.036	0.047
0	500	0	3	10	0	0	0	0.049	0.051	0.05	0.053	0.043	0.059	0.057	0.061	0.064	0.061	0.057	0.05	0.048	0.053	0.056
0	100	0.1	3	10	0	0	0	0.038	0.038	0.043	0.041	0.034	0.05	0.049	0.049	0.05	0.052	0.047	0.042	0.048	0.045	0.047
0	500	0.1	3	10	0	0	0	0.058	0.052	0.059	0.05	0.048	0.064	0.062	0.058	0.058	0.055	0.06	0.053	0.055	0.063	0.056
0	100	0.3	3	10	0	0	0	0.048	0.047	0.045	0.049	0.052	0.042	0.042	0.044	0.049	0.047	0.044	0.037	0.041	0.043	0.047
0	500	0.3	3	10	0	0	0	0.051	0.058	0.051	0.051	0.055	0.052	0.056	0.054	0.054	0.048	0.053	0.053	0.052	0.045	0.056
0	100	0	3	50	0	0	0	0.051	0.047	0.053	0.044	0.037	0.042	0.049	0.046	0.056	0.055	0.047	0.047	0.069	0.057	0.043
0	500	0	3	50	0	0	0	0.045	0.05	0.047	0.052	0.041	0.05	0.047	0.048	0.054	0.054	0.048	0.047	0.045	0.046	0.045
0	100	0.1	3	50	0	0	0	0.057	0.057	0.057	0.06	0.057	0.053	0.056	0.059	0.065	0.06	0.053	0.049	0.048	0.052	0.043
0	500	0.1	3	50	0	0	0	0.042	0.051	0.048	0.042	0.042	0.049	0.049	0.044	0.047	0.05	0.05	0.05	0.047	0.043	0.045
0	100	0.3	3	50	0	0	0	0.054	0.055	0.053	0.054	0.056	0.055	0.056	0.056	0.057	0.051	0.054	0.046	0.047	0.046	0.043

0	500	0.3	3	50	0	0	0	0.052	0.05	0.05	0.049	0.048	0.047	0.049	0.047	0.045	0.045	0.048	0.046	0.049	0.048	0.045
0	100	0	3	100	0	0	0	0.047	0.048	0.053	0.047	0.047	0.066	0.06	0.055	0.051	0.051	0.055	0.043	0.049	0.054	NA
0	500	0	3	100	0	0	0	0.056	0.055	0.056	0.05	0.044	0.058	0.056	0.054	0.052	0.043	0.052	0.061	0.039	0.045	0.055
0	100	0.1	3	100	0	0	0	0.047	0.044	0.052	0.049	0.048	0.049	0.05	0.053	0.047	0.055	0.048	0.047	0.046	0.057	NA
0	500	0.1	3	100	0	0	0	0.051	0.055	0.049	0.054	0.051	0.044	0.045	0.052	0.048	0.046	0.049	0.068	0.05	0.048	0.055
0	100	0.3	3	100	0	0	0	0.048	0.048	0.05	0.049	0.049	0.051	0.049	0.051	0.049	0.045	0.052	0.05	0.045	0.055	NA
0	500	0.3	3	100	0	0	0	0.05	0.053	0.05	0.051	0.05	0.049	0.044	0.043	0.045	0.045	0.053	0.051	0.051	0.048	0.055
0	100	0	3	500	0	0	0	0.045	0.046	0.048	0.038	0.037	0.058	0.06	0.064	0.053	0.05	0.052	0.055	0.047	0.049	NA
0	500	0	3	500	0	0	0	0.057	0.046	0.057	0.053	0.049	0.055	0.058	0.061	0.049	0.058	0.062	0.051	0.057	0.051	NA
0	100	0.1	3	500	0	0	0	0.054	0.054	0.052	0.056	0.059	0.052	0.055	0.053	0.053	0.052	0.055	0.059	0.053	0.048	NA
0	500	0.1	3	500	0	0	0	0.05	0.05	0.054	0.046	0.044	0.055	0.055	0.057	0.058	0.063	0.054	0.05	0.049	0.05	NA
0	100	0.3	3	500	0	0	0	0.052	0.055	0.051	0.051	0.053	0.053	0.052	0.054	0.057	0.055	0.053	0.048	0.054	0.052	NA
0	500	0.3	3	500	0	0	0	0.049	0.049	0.049	0.05	0.047	0.05	0.051	0.051	0.052	0.061	0.053	0.053	0.05	0.061	NA
0	100	0	6	10	0	0	0	0.048	0.047	0.049	0.049	0.048	0.043	0.046	0.051	0.05	0.05	0.045	0.048	0.053	0.036	0.047
0	500	0	6	10	0	0	0	0.049	0.051	0.05	0.053	0.043	0.059	0.057	0.061	0.064	0.061	0.057	0.05	0.048	0.053	0.056
0	100	0.1	6	10	0	0	0	0.038	0.038	0.043	0.041	0.034	0.05	0.049	0.049	0.05	0.052	0.047	0.042	0.048	0.045	0.047
0	500	0.1	6	10	0	0	0	0.058	0.052	0.059	0.05	0.048	0.064	0.062	0.058	0.058	0.055	0.06	0.053	0.055	0.063	0.056
0	100	0.3	6	10	0	0	0	0.048	0.047	0.045	0.049	0.052	0.042	0.042	0.044	0.049	0.047	0.044	0.037	0.041	0.043	0.047
0	500	0.3	6	10	0	0	0	0.051	0.058	0.051	0.051	0.055	0.052	0.056	0.054	0.054	0.048	0.053	0.053	0.052	0.045	0.056
0	100	0	6	50	0	0	0	0.051	0.047	0.053	0.044	0.037	0.042	0.049	0.046	0.056	0.055	0.047	0.047	0.069	0.057	0.043
0	500	0	6	50	0	0	0	0.045	0.05	0.047	0.052	0.041	0.05	0.047	0.048	0.054	0.054	0.048	0.047	0.045	0.046	0.045
0	100	0.1	6	50	0	0	0	0.057	0.057	0.057	0.06	0.057	0.053	0.056	0.059	0.065	0.06	0.053	0.049	0.048	0.052	0.043
0	500	0.1	6	50	0	0	0	0.042	0.051	0.048	0.042	0.042	0.049	0.049	0.044	0.047	0.05	0.05	0.05	0.047	0.043	0.045
0	100	0.3	6	50	0	0	0	0.054	0.055	0.053	0.054	0.056	0.055	0.056	0.056	0.057	0.051	0.054	0.046	0.047	0.046	0.043
0	500	0.3	6	50	0	0	0	0.052	0.05	0.05	0.049	0.048	0.047	0.049	0.047	0.045	0.045	0.048	0.046	0.049	0.048	0.045
0	100	0	6	100	0	0	0	0.047	0.048	0.053	0.047	0.047	0.066	0.06	0.055	0.051	0.051	0.055	0.043	0.049	0.054	NA
0	500	0	6	100	0	0	0	0.056	0.055	0.056	0.05	0.044	0.058	0.056	0.054	0.052	0.043	0.052	0.061	0.039	0.045	0.055
0	100	0.1	6	100	0	0	0	0.047	0.044	0.052	0.049	0.048	0.049	0.05	0.053	0.047	0.055	0.048	0.047	0.046	0.057	NA
0	500	0.1	6	100	0	0	0	0.051	0.055	0.049	0.054	0.051	0.044	0.045	0.052	0.048	0.046	0.049	0.068	0.05	0.048	0.055
0	100	0.3	6	100	0	0	0	0.048	0.048	0.05	0.049	0.049	0.051	0.049	0.051	0.049	0.045	0.052	0.05	0.045	0.055	NA
0	500	0.3	6	100	0	0	0	0.05	0.053	0.05	0.051	0.05	0.049	0.044	0.043	0.045	0.045	0.053	0.051	0.051	0.048	0.055
0	100	0	6	500	0	0	0	0.045	0.046	0.048	0.038	0.037	0.058	0.06	0.064	0.053	0.05	0.052	0.055	0.047	0.049	NA
0	500	0	6	500	0	0	0	0.057	0.046	0.057	0.053	0.049	0.055	0.058	0.061	0.049	0.058	0.062	0.051	0.057	0.051	NA
0	100	0.1	6	500	0	0	0	0.054	0.054	0.052	0.056	0.059	0.052	0.055	0.053	0.053	0.052	0.055	0.059	0.053	0.048	NA
0	500	0.1	6	500	0	0	0	0.05	0.05	0.054	0.046	0.044	0.055	0.055	0.057	0.058	0.063	0.054	0.05	0.049	0.05	NA
0	100	0.3	6	500	0	0	0	0.052	0.055	0.051	0.051	0.053	0.053	0.052	0.054	0.057	0.055	0.053	0.048	0.054	0.052	NA
0	500	0.3	6	500	0	0	0	0.049	0.049	0.049	0.05	0.047	0.05	0.051	0.051	0.052	0.061	0.053	0.053	0.05	0.061	NA
1	100	0	1	10	0	0	1	0.182	0.101	1	0.999	1	1	1	1	1	1	1	1	0.708	1	1
1	500	0	1	10	0	0	1	0.193	0.096	1	1	1	1	1	1	1	1	1	1	0.845	1	1
1	100	0.1	1	10	0	0	1	0.657	0.487	1	0.998	1	1	1	1	1	1	1	1	0.903	1	1

1	500	0.1	1	10	0	0	1	0.995	0.986	1	1	1	1	1	1	1	1	1	1	1	1	1
1	100	0.3	1	10	0	0	1	1	1	1	1	1	1	1	1	1	1	1	1	1	1	1
1	500	0.3	1	10	0	0	1	1	1	1	1	1	1	1	1	1	1	1	1	1	1	1
1	100	0	1	50	0	0	1	0.121	0.058	1	0.706	0.892	1	1	1	1	1	1	1	0.42	0.876	1
1	500	0	1	50	0	0	1	0.119	0.064	1	1	1	1	1	1	1	1	1	1	0.627	0.988	1
1	100	0.1	1	50	0	0	1	0.79	0.7	1	0.957	0.983	1	1	1	1	1	1	1	0.883	0.974	1
1	500	0.1	1	50	0	0	1	1	1	1	1	1	1	1	1	1	1	1	1	1	1	1
1	100	0.3	1	50	0	0	1	1	1	1	1	1	1	1	1	1	1	1	1	1	1	1
1	500	0.3	1	50	0	0	1	1	1	1	1	1	1	1	1	1	1	1	1	1	1	1
1	100	0	1	100	0	0	1	0.114	0.067	1	0.447	0.536	1	1	1	1	1	1	1	0.332	0.714	NA
1	500	0	1	100	0	0	1	0.088	0.052	1	0.951	1	1	1	1	1	1	1	1	0.529	0.958	1
1	100	0.1	1	100	0	0	1	0.852	0.789	1	0.934	0.944	1	1	1	1	1	1	1	0.886	0.944	NA
1	500	0.1	1	100	0	0	1	1	1	1	1	1	1	1	1	1	1	1	1	1	1	1
1	100	0.3	1	100	0	0	1	0.999	0.999	1	0.999	0.999	1	1	1	1	1	1	1	0.999	1	NA
1	500	0.3	1	100	0	0	1	1	1	1	1	1	1	1	1	1	1	1	1	1	1	1
1	100	0	1	500	0	0	1	0.099	0.066	1	0.18	0.165	1	1	1	1	1	0.891	0.809	0.19	0.351	NA
1	500	0	1	500	0	0	1	0.08	0.067	1	0.46	0.919	1	1	1	1	1	1	1	0.315	0.687	NA
1	100	0.1	1	500	0	0	1	0.852	0.841	0.987	0.878	0.866	1	1	1	1	1	0.945	0.909	0.861	0.799	NA
1	500	0.1	1	500	0	0	1	1	1	1	1	1	1	1	1	1	1	1	1	1	1	NA
1	100	0.3	1	500	0	0	1	1	1	1	1	1	1	1	1	1	1	1	0.995	1	0.999	NA
1	500	0.3	1	500	0	0	1	1	1	1	1	1	1	1	1	1	1	1	1	1	1	NA
1	100	0	3	10	0	0	1	0.216	0.111	1	0.814	0.953	1	1	1	1	1	1	1	0.6	1	1
1	500	0	3	10	0	0	1	0.206	0.107	1	1	1	1	1	1	1	1	1	1	0.792	1	1
1	100	0.1	3	10	0	0	1	0.496	0.319	1	0.929	0.978	1	1	1	1	1	1	1	0.81	1	1
1	500	0.1	3	10	0	0	1	0.94	0.883	1	1	1	1	1	1	1	1	1	1	1	1	1
1	100	0.3	3	10	0	0	1	0.978	0.96	1	0.999	0.998	1	1	1	1	1	1	1	0.996	1	1
1	500	0.3	3	10	0	0	1	1	1	1	1	1	1	1	1	1	1	1	1	1	1	1
1	100	0	3	50	0	0	1	0.128	0.061	0.993	0.316	0.225	1	1	1	1	1	0.999	0.995	0.294	0.642	0.996
1	500	0	3	50	0	0	1	0.115	0.051	1	0.816	1	1	1	1	1	1	1	1	0.53	0.953	1
1	100	0.1	3	50	0	0	1	0.55	0.45	0.997	0.67	0.611	1	1	1	1	1	0.998	0.998	0.665	0.822	0.992
1	500	0.1	3	50	0	0	1	0.997	0.992	1	1	1	1	1	1	1	1	1	1	1	0.999	1
1	100	0.3	3	50	0	0	1	0.986	0.982	0.999	0.991	0.988	1	1	1	1	1	1	0.999	0.987	0.988	0.995
1	500	0.3	3	50	0	0	1	1	1	1	1	1	1	1	1	1	1	1	1	1	1	1
1	100	0	3	100	0	0	1	0.114	0.078	0.958	0.217	0.15	1	1	1	1	1	0.961	0.914	0.228	0.449	NA
1	500	0	3	100	0	0	1	0.094	0.061	1	0.582	0.841	1	1	1	1	1	1	1	0.421	0.88	1
1	100	0.1	3	100	0	0	1	0.579	0.522	0.946	0.649	0.592	1	1	1	1	1	0.944	0.942	0.63	0.694	NA
1	500	0.1	3	100	0	0	1	0.998	0.996	1	0.998	0.999	1	1	1	1	1	1	1	0.998	1	1
1	100	0.3	3	100	0	0	1	0.983	0.985	0.993	0.988	0.985	0.999	0.999	1	1	1	0.994	0.967	0.985	0.967	NA
1	500	0.3	3	100	0	0	1	1	1	1	1	1	1	1	1	1	1	1	1	1	1	1
1	100	0	3	500	0	0	1	0.086	0.059	0.5	0.111	0.083	0.968	0.997	1	1	1	0.486	0.35	0.126	0.212	NA

1	500	0	3	500	0	0	1	0.086	0.051	1	0.211	0.237	1	1	1	1	1	1	1	0.215	0.457	NA
1	100	0.1	3	500	0	0	1	0.613	0.599	0.706	0.627	0.61	0.886	0.935	0.988	1	1	0.708	0.412	0.628	0.513	NA
1	500	0.1	3	500	0	0	1	1	1	1	1	1	1	1	1	1	1	1	1	1	0.996	NA
1	100	0.3	3	500	0	0	1	0.987	0.987	0.991	0.99	0.99	0.993	0.995	0.996	1	1	0.99	0.618	0.988	0.943	NA
1	500	0.3	3	500	0	0	1	1	1	1	1	1	1	1	1	1	1	1	1	1	1	NA
1	100	0	6	10	0	0	1	0.215	0.12	0.911	0.435	0.332	0.98	0.983	0.982	0.983	0.983	0.945	0.893	0.373	0.809	0.952
1	500	0	6	10	0	0	1	0.212	0.111	1	0.946	0.999	1	1	1	1	1	1	1	0.676	1	1
1	100	0.1	6	10	0	0	1	0.296	0.179	0.913	0.515	0.405	0.981	0.983	0.987	0.989	0.988	0.941	0.901	0.518	0.835	0.932
1	500	0.1	6	10	0	0	1	0.67	0.519	1	0.991	1	1	1	1	1	1	1	1	0.985	1	1
1	100	0.3	6	10	0	0	1	0.733	0.635	0.934	0.816	0.767	0.974	0.983	0.986	0.991	0.988	0.949	0.912	0.815	0.884	0.931
1	500	0.3	6	10	0	0	1	0.998	0.997	1	1	1	1	1	1	1	1	1	1	1	1	1
1	100	0	6	50	0	0	1	0.126	0.062	0.492	0.166	0.12	0.839	0.894	0.922	0.938	0.949	0.559	0.479	0.161	0.305	0.394
1	500	0	6	50	0	0	1	0.115	0.055	1	0.42	0.388	1	1	1	1	1	1	1	0.32	0.773	1
1	100	0.1	6	50	0	0	1	0.291	0.218	0.546	0.332	0.267	0.809	0.862	0.908	0.942	0.948	0.583	0.524	0.327	0.394	0.394
1	500	0.1	6	50	0	0	1	0.822	0.752	1	0.92	0.904	1	1	1	1	1	1	1	0.937	0.953	1
1	100	0.3	6	50	0	0	1	0.708	0.686	0.787	0.718	0.701	0.843	0.879	0.915	0.952	0.96	0.791	0.578	0.726	0.629	0.381
1	500	0.3	6	50	0	0	1	1	1	1	1	1	1	1	1	1	1	1	1	1	1	1
1	100	0	6	100	0	0	1	0.109	0.074	0.333	0.123	0.088	0.666	0.753	0.827	0.877	0.907	0.393	0.311	0.129	0.194	NA
1	500	0	6	100	0	0	1	0.112	0.069	0.999	0.291	0.237	1	1	1	1	1	1	0.999	0.227	0.56	1
1	100	0.1	6	100	0	0	1	0.286	0.243	0.403	0.296	0.258	0.618	0.703	0.806	0.882	0.916	0.428	0.324	0.299	0.302	NA
1	500	0.1	6	100	0	0	1	0.869	0.818	0.999	0.914	0.892	1	1	1	1	1	1	1	0.917	0.9	1
1	100	0.3	6	100	0	0	1	0.673	0.659	0.705	0.682	0.67	0.742	0.768	0.817	0.884	0.931	0.707	0.409	0.678	0.53	NA
1	500	0.3	6	100	0	0	1	0.999	0.999	1	1	1	1	1	1	1	1	1	1	1	1	1
1	100	0	6	500	0	0	1	0.087	0.049	0.145	0.079	0.064	0.323	0.394	0.509	0.661	0.81	0.159	0.123	0.071	0.111	NA
1	500	0	6	500	0	0	1	0.083	0.053	0.791	0.116	0.095	1	1	1	1	1	0.908	0.817	0.109	0.224	NA
1	100	0.1	6	500	0	0	1	0.305	0.292	0.327	0.305	0.295	0.365	0.382	0.436	0.592	0.817	0.337	0.141	0.306	0.208	NA
1	500	0.1	6	500	0	0	1	0.887	0.88	0.942	0.891	0.883	1	1	1	1	1	0.959	0.861	0.896	0.783	NA
1	100	0.3	6	500	0	0	1	0.721	0.719	0.728	0.721	0.721	0.731	0.741	0.748	0.77	0.853	0.727	0.208	0.719	0.48	NA
1	500	0.3	6	500	0	0	1	1	1	1	1	1	1	1	1	1	1	1	0.929	1	0.998	NA
1	100	0	1	10	0	0	2	0.469	0.197	1	1	1	1	1	1	1	1	1	1	0.651	0.996	1
1	500	0	1	10	0	0	2	0.46	0.188	1	1	1	1	1	1	1	1	1	1	0.822	1	1
1	100	0.1	1	10	0	0	2	0.946	0.833	1	1	1	1	1	1	1	1	1	1	0.98	1	1
1	500	0.1	1	10	0	0	2	1	1	1	1	1	1	1	1	1	1	1	1	1	1	1
1	100	0.3	1	10	0	0	2	1	1	1	1	1	1	1	1	1	1	1	1	1	1	1
1	500	0.3	1	10	0	0	2	1	1	1	1	1	1	1	1	1	1	1	1	1	1	1
1	100	0	1	50	0	0	2	0.201	0.079	1	0.691	0.588	1	1	1	1	1	1	1	0.393	0.862	1
1	500	0	1	50	0	0	2	0.224	0.1	1	1	1	1	1	1	1	1	1	1	0.617	0.985	1
1	100	0.1	1	50	0	0	2	0.982	0.962	1	0.997	0.995	1	1	1	1	1	1	1	0.99	0.997	1
1	500	0.1	1	50	0	0	2	1	1	1	1	1	1	1	1	1	1	1	1	1	1	1
1	100	0.3	1	50	0	0	2	1	1	1	1	1	1	1	1	1	1	1	1	1	1	1

1	500	0.3	1	50	0	0	2	1	1	1	1	1	1	1	1	1	1	1	1	1	1	1
1	100	0	1	100	0	0	2	0.187	0.085	1	0.482	0.345	1	1	1	1	1	1	1	0.33	0.683	NA
1	500	0	1	100	0	0	2	0.183	0.072	1	0.972	1	1	1	1	1	1	1	1	0.562	0.968	1
1	100	0.1	1	100	0	0	2	0.989	0.979	1	0.997	0.993	1	1	1	1	1	1	1	0.997	0.992	NA
1	500	0.1	1	100	0	0	2	1	1	1	1	1	1	1	1	1	1	1	1	1	1	1
1	100	0.3	1	100	0	0	2	1	1	1	1	1	1	1	1	1	1	1	1	1	1	NA
1	500	0.3	1	100	0	0	2	1	1	1	1	1	1	1	1	1	1	1	1	1	1	1
1	100	0	1	500	0	0	2	0.133	0.073	0.879	0.187	0.131	1	1	1	1	1	0.882	0.833	0.172	0.338	NA
1	500	0	1	500	0	0	2	0.107	0.055	1	0.501	0.61	1	1	1	1	1	1	1	0.327	0.724	NA
1	100	0.1	1	500	0	0	2	0.991	0.992	0.998	0.995	0.994	1	1	1	1	1	0.997	0.981	0.992	0.965	NA
1	500	0.1	1	500	0	0	2	1	1	1	1	1	1	1	1	1	1	1	1	1	1	NA
1	100	0.3	1	500	0	0	2	1	1	1	1	1	1	1	1	1	1	1	1	1	1	NA
1	500	0.3	1	500	0	0	2	1	1	1	1	1	1	1	1	1	1	1	1	1	1	NA
1	100	0	3	10	0	0	2	0.45	0.199	1	0.989	0.999	1	1	1	1	1	1	0.999	0.588	0.989	1
1	500	0	3	10	0	0	2	0.459	0.203	1	1	1	1	1	1	1	1	1	1	0.783	1	1
1	100	0.1	3	10	0	0	2	0.893	0.739	1	1	1	1	1	1	1	1	1	1	0.969	1	1
1	500	0.1	3	10	0	0	2	1	1	1	1	1	1	1	1	1	1	1	1	1	1	1
1	100	0.3	3	10	0	0	2	1	1	1	1	1	1	1	1	1	1	1	1	1	1	1
1	500	0.3	3	10	0	0	2	1	1	1	1	1	1	1	1	1	1	1	1	1	1	1
1	100	0	3	50	0	0	2	0.22	0.092	0.999	0.524	0.373	1	1	1	1	1	1	1	0.317	0.744	1
1	500	0	3	50	0	0	2	0.214	0.088	1	0.989	1	1	1	1	1	1	1	1	0.561	0.967	1
1	100	0.1	3	50	0	0	2	0.941	0.868	1	0.978	0.956	1	1	1	1	1	1	1	0.975	0.979	1
1	500	0.1	3	50	0	0	2	1	1	1	1	1	1	1	1	1	1	1	1	1	1	1
1	100	0.3	3	50	0	0	2	1	1	1	1	1	1	1	1	1	1	1	1	1	1	1
1	500	0.3	3	50	0	0	2	1	1	1	1	1	1	1	1	1	1	1	1	1	1	1
1	100	0	3	100	0	0	2	0.202	0.091	0.983	0.359	0.225	1	1	1	1	1	0.997	0.992	0.271	0.548	NA
1	500	0	3	100	0	0	2	0.169	0.074	1	0.885	0.985	1	1	1	1	1	1	1	0.472	0.942	1
1	100	0.1	3	100	0	0	2	0.95	0.91	0.999	0.965	0.949	1	1	1	1	1	1	1	0.964	0.958	NA
1	500	0.1	3	100	0	0	2	1	1	1	1	1	1	1	1	1	1	1	1	1	1	1
1	100	0.3	3	100	0	0	2	1	1	1	1	1	1	1	1	1	1	1	1	1	1	NA
1	500	0.3	3	100	0	0	2	1	1	1	1	1	1	1	1	1	1	1	1	1	1	1
1	100	0	3	500	0	0	2	0.13	0.069	0.615	0.167	0.113	0.981	0.996	1	1	1	0.668	0.551	0.137	0.27	NA
1	500	0	3	500	0	0	2	0.124	0.061	1	0.357	0.331	1	1	1	1	1	1	1	0.259	0.587	NA
1	100	0.1	3	500	0	0	2	0.957	0.951	0.978	0.96	0.951	0.997	0.999	1	1	1	0.98	0.752	0.959	0.876	NA
1	500	0.1	3	500	0	0	2	1	1	1	1	1	1	1	1	1	1	1	1	1	1	NA
1	100	0.3	3	500	0	0	2	1	1	1	1	1	1	1	1	1	1	1	0.984	1	1	NA
1	500	0.3	3	500	0	0	2	1	1	1	1	1	1	1	1	1	1	1	1	1	1	NA
1	100	0	6	10	0	0	2	0.477	0.205	0.997	0.832	0.754	0.998	0.999	0.999	0.999	0.998	0.998	0.987	0.464	0.916	0.999
1	500	0	6	10	0	0	2	0.478	0.205	1	1	1	1	1	1	1	1	1	1	0.702	0.998	1
1	100	0.1	6	10	0	0	2	0.76	0.522	1	0.95	0.933	1	1	1	1	1	1	0.999	0.902	0.993	1

1	500	0.1	6	10	0	0	2	0.995	0.973	1	1	1	1	1	1	1	1	1	1	1	1	1
1	100	0.3	6	10	0	0	2	0.997	0.991	1	1	1	1	1	1	1	1	1	1	1	1	1
1	500	0.3	6	10	0	0	2	1	1	1	1	1	1	1	1	1	1	1	1	1	1	1
1	100	0	6	50	0	0	2	0.235	0.097	0.814	0.312	0.181	0.971	0.981	0.987	0.991	0.987	0.875	0.829	0.209	0.428	0.788
1	500	0	6	50	0	0	2	0.218	0.097	1	0.846	0.865	1	1	1	1	1	1	1	0.429	0.883	1
1	100	0.1	6	50	0	0	2	0.709	0.585	0.953	0.755	0.678	0.992	0.995	0.997	0.998	0.996	0.969	0.9	0.787	0.778	0.835
1	500	0.1	6	50	0	0	2	1	1	1	1	1	1	1	1	1	1	1	1	1	1	1
1	100	0.3	6	50	0	0	2	0.998	0.998	0.999	0.998	0.997	1	1	1	1	1	1	0.981	0.998	0.989	0.914
1	500	0.3	6	50	0	0	2	1	1	1	1	1	1	1	1	1	1	1	1	1	1	1
1	100	0	6	100	0	0	2	0.193	0.075	0.619	0.198	0.118	0.918	0.948	0.971	0.98	0.981	0.69	0.606	0.163	0.327	NA
1	500	0	6	100	0	0	2	0.194	0.078	1	0.618	0.547	1	1	1	1	1	1	1	0.332	0.78	1
1	100	0.1	6	100	0	0	2	0.739	0.645	0.881	0.765	0.694	0.976	0.987	0.993	0.994	0.994	0.905	0.736	0.76	0.683	NA
1	500	0.1	6	100	0	0	2	0.998	0.998	1	0.998	0.998	1	1	1	1	1	1	1	0.999	0.999	1
1	100	0.3	6	100	0	0	2	0.998	0.998	0.999	0.999	0.998	0.999	0.999	1	1	1	0.999	0.903	0.998	0.983	NA
1	500	0.3	6	100	0	0	2	1	1	1	1	1	1	1	1	1	1	1	1	1	1	1
1	100	0	6	500	0	0	2	0.128	0.072	0.252	0.104	0.079	0.527	0.634	0.749	0.859	0.907	0.286	0.217	0.097	0.143	NA
1	500	0	6	500	0	0	2	0.117	0.062	0.984	0.193	0.126	1	1	1	1	1	0.998	0.993	0.148	0.342	NA
1	100	0.1	6	500	0	0	2	0.742	0.728	0.783	0.745	0.729	0.82	0.841	0.898	0.954	0.982	0.786	0.29	0.746	0.548	NA
1	500	0.1	6	500	0	0	2	1	1	1	1	1	1	1	1	1	1	1	0.999	1	0.999	NA
1	100	0.3	6	500	0	0	2	0.996	0.997	0.997	0.996	0.997	0.998	0.998	0.999	0.999	1	0.999	0.551	0.998	0.975	NA
1	500	0.3	6	500	0	0	2	1	1	1	1	1	1	1	1	1	1	1	1	1	1	NA
1	100	0	1	10	0	0	5	0.999	1	1	1	1	1	1	1	1	1	1	0.998	0.667	0.993	1
1	500	0	1	10	0	0	5	0.999	1	1	1	1	1	1	1	1	1	1	1	0.808	0.999	1
1	100	0.1	1	10	0	0	5	1	1	1	1	1	1	1	1	1	1	1	1	0.999	1	1
1	500	0.1	1	10	0	0	5	1	1	1	1	1	1	1	1	1	1	1	1	1	1	1
1	100	0.3	1	10	0	0	5	1	1	1	1	1	1	1	1	1	1	1	1	1	1	1
1	500	0.3	1	10	0	0	5	1	1	1	1	1	1	1	1	1	1	1	1	1	1	1
1	100	0	1	50	0	0	5	0.675	0.276	1	0.937	0.813	1	1	1	1	1	1	1	0.431	0.853	1
1	500	0	1	50	0	0	5	0.719	0.265	1	1	1	1	1	1	1	1	1	1	0.643	0.992	1
1	100	0.1	1	50	0	0	5	1	1	1	1	1	1	1	1	1	1	1	1	1	1	1
1	500	0.1	1	50	0	0	5	1	1	1	1	1	1	1	1	1	1	1	1	1	1	1
1	100	0.3	1	50	0	0	5	1	1	1	1	1	1	1	1	1	1	1	1	1	1	1
1	500	0.3	1	50	0	0	5	1	1	1	1	1	1	1	1	1	1	1	1	1	1	1
1	100	0	1	100	0	0	5	0.506	0.172	1	0.705	0.471	1	1	1	1	1	1	1	0.302	0.671	NA
1	500	0	1	100	0	0	5	0.536	0.169	1	1	1	1	1	1	1	1	1	1	0.55	0.958	1
1	100	0.1	1	100	0	0	5	1	1	1	1	1	1	1	1	1	1	1	1	1	1	NA
1	500	0.1	1	100	0	0	5	1	1	1	1	1	1	1	1	1	1	1	1	1	1	1
1	100	0.3	1	100	0	0	5	1	1	1	1	1	1	1	1	1	1	1	1	1	1	NA
1	500	0.3	1	100	0	0	5	1	1	1	1	1	1	1	1	1	1	1	1	1	1	1
1	100	0	1	500	0	0	5	0.275	0.09	0.833	0.279	0.148	0.997	1	1	1	1	0.903	0.856	0.153	0.328	NA

1	500	0	1	500	0	0	5	0.254	0.073	1	0.743	0.611	1	1	1	1	1	1	1	0.331	0.706	NA
1	100	0.1	1	500	0	0	5	1	1	1	1	1	1	1	1	1	1	1	0.999	1	1	NA
1	500	0.1	1	500	0	0	5	1	1	1	1	1	1	1	1	1	1	1	1	1	1	NA
1	100	0.3	1	500	0	0	5	1	1	1	1	1	1	1	1	1	1	1	1	1	1	NA
1	500	0.3	1	500	0	0	5	1	1	1	1	1	1	1	1	1	1	1	1	1	1	NA
1	100	0	3	10	0	0	5	0.999	1	1	1	1	1	1	1	1	1	1	0.999	0.642	0.984	1
1	500	0	3	10	0	0	5	0.999	1	1	1	1	1	1	1	1	1	1	1	0.792	1	1
1	100	0.1	3	10	0	0	5	1	1	1	1	1	1	1	1	1	1	1	1	0.999	1	1
1	500	0.1	3	10	0	0	5	1	1	1	1	1	1	1	1	1	1	1	1	1	1	1
1	100	0.3	3	10	0	0	5	1	1	1	1	1	1	1	1	1	1	1	1	1	1	1
1	500	0.3	3	10	0	0	5	1	1	1	1	1	1	1	1	1	1	1	1	1	1	1
1	100	0	3	50	0	0	5	0.676	0.263	1	0.894	0.712	1	1	1	1	1	1	1	0.405	0.803	1
1	500	0	3	50	0	0	5	0.711	0.262	1	1	1	1	1	1	1	1	1	1	0.62	0.986	1
1	100	0.1	3	50	0	0	5	1	1	1	1	1	1	1	1	1	1	1	1	1	1	1
1	500	0.1	3	50	0	0	5	1	1	1	1	1	1	1	1	1	1	1	1	1	1	1
1	100	0.3	3	50	0	0	5	1	1	1	1	1	1	1	1	1	1	1	1	1	1	1
1	500	0.3	3	50	0	0	5	1	1	1	1	1	1	1	1	1	1	1	1	1	1	1
1	100	0	3	100	0	0	5	0.508	0.18	0.997	0.643	0.392	1	1	1	1	1	0.999	0.998	0.282	0.605	NA
1	500	0	3	100	0	0	5	0.54	0.165	1	1	1	1	1	1	1	1	1	1	0.523	0.948	1
1	100	0.1	3	100	0	0	5	1	1	1	1	1	1	1	1	1	1	1	1	1	1	NA
1	500	0.1	3	100	0	0	5	1	1	1	1	1	1	1	1	1	1	1	1	1	1	1
1	100	0.3	3	100	0	0	5	1	1	1	1	1	1	1	1	1	1	1	1	1	1	NA
1	500	0.3	3	100	0	0	5	1	1	1	1	1	1	1	1	1	1	1	1	1	1	1
1	100	0	3	500	0	0	5	0.266	0.095	0.718	0.246	0.147	0.985	0.999	0.999	1	1	0.795	0.74	0.131	0.275	NA
1	500	0	3	500	0	0	5	0.248	0.087	1	0.663	0.51	1	1	1	1	1	1	1	0.3	0.653	NA
1	100	0.1	3	500	0	0	5	1	1	1	1	1	1	1	1	1	1	1	0.989	1	1	NA
1	500	0.1	3	500	0	0	5	1	1	1	1	1	1	1	1	1	1	1	1	1	1	NA
1	100	0.3	3	500	0	0	5	1	1	1	1	1	1	1	1	1	1	1	1	1	1	NA
1	500	0.3	3	500	0	0	5	1	1	1	1	1	1	1	1	1	1	1	1	1	1	NA
1	100	0	6	10	0	0	5	0.997	0.995	1	1	1	1	1	1	1	1	1	0.997	0.556	0.952	1
1	500	0	6	10	0	0	5	1	1	1	1	1	1	1	1	1	1	1	1	0.753	0.998	1
1	100	0.1	6	10	0	0	5	1	1	1	1	1	1	1	1	1	1	1	1	0.998	1	1
1	500	0.1	6	10	0	0	5	1	1	1	1	1	1	1	1	1	1	1	1	1	1	1
1	100	0.3	6	10	0	0	5	1	1	1	1	1	1	1	1	1	1	1	1	1	1	1
1	500	0.3	6	10	0	0	5	1	1	1	1	1	1	1	1	1	1	1	1	1	1	1
1	100	0	6	50	0	0	5	0.644	0.249	0.99	0.708	0.489	1	1	0.999	0.999	0.992	0.994	0.99	0.304	0.674	0.998
1	500	0	6	50	0	0	5	0.706	0.265	1	1	1	1	1	1	1	1	1	1	0.544	0.959	1
1	100	0.1	6	50	0	0	5	1	0.996	1	1	0.999	1	1	1	1	1	1	1	1	0.999	1
1	500	0.1	6	50	0	0	5	1	1	1	1	1	1	1	1	1	1	1	1	1	1	1
1	100	0.3	6	50	0	0	5	1	1	1	1	1	1	1	1	1	1	1	1	1	1	1

1	500	0.3	6	50	0	0	5	1	1	1	1	1	1	1	1	1	1	1	1	1	1	1
1	100	0	6	100	0	0	5	0.475	0.155	0.92	0.482	0.293	0.996	0.997	0.999	0.998	0.991	0.953	0.931	0.214	0.444	NA
1	500	0	6	100	0	0	5	0.562	0.167	1	0.984	0.969	1	1	1	1	1	1	1	0.45	0.895	1
1	100	0.1	6	100	0	0	5	1	0.998	1	1	0.999	1	1	1	1	1	1	0.998	1	0.998	NA
1	500	0.1	6	100	0	0	5	1	1	1	1	1	1	1	1	1	1	1	1	1	1	1
1	100	0.3	6	100	0	0	5	1	1	1	1	1	1	1	1	1	1	1	1	1	1	NA
1	500	0.3	6	100	0	0	5	1	1	1	1	1	1	1	1	1	1	1	1	1	1	1
1	100	0	6	500	0	0	5	0.243	0.088	0.456	0.194	0.127	0.791	0.872	0.92	0.953	0.94	0.534	0.402	0.101	0.189	NA
1	500	0	6	500	0	0	5	0.252	0.071	1	0.481	0.302	1	1	1	1	1	1	1	0.217	0.508	NA
1	100	0.1	6	500	0	0	5	1	1	1	1	1	1	1	1	1	1	1	0.817	1	0.997	NA
1	500	0.1	6	500	0	0	5	1	1	1	1	1	1	1	1	1	1	1	1	1	1	NA
1	100	0.3	6	500	0	0	5	1	1	1	1	1	1	1	1	1	1	1	1	1	1	NA
1	500	0.3	6	500	0	0	5	1	1	1	1	1	1	1	1	1	1	1	1	1	1	NA
1	100	0	1	10	0	0	10	1	1	1	1	1	1	1	1	1	1	1	1	0.633	0.99	1
1	500	0	1	10	0	0	10	1	1	1	1	1	1	1	1	1	1	1	1	0.822	1	1
1	100	0.1	1	10	0	0	10	1	1	1	1	1	1	1	1	1	1	1	1	0.999	1	1
1	500	0.1	1	10	0	0	10	1	1	1	1	1	1	1	1	1	1	1	1	1	1	1
1	100	0.3	1	10	0	0	10	1	1	1	1	1	1	1	1	1	1	1	1	1	1	1
1	500	0.3	1	10	0	0	10	1	1	1	1	1	1	1	1	1	1	1	1	1	1	1
1	100	0	1	50	0	0	10	0.988	0.9	1	0.997	0.967	1	1	1	1	0.999	1	1	0.43	0.85	1
1	500	0	1	50	0	0	10	0.993	1	1	1	1	1	1	1	1	1	1	1	0.617	0.991	1
1	100	0.1	1	50	0	0	10	1	1	1	1	1	1	1	1	1	1	1	1	1	1	1
1	500	0.1	1	50	0	0	10	1	1	1	1	1	1	1	1	1	1	1	1	1	1	1
1	100	0.3	1	50	0	0	10	1	1	1	1	1	1	1	1	1	1	1	1	1	1	1
1	500	0.3	1	50	0	0	10	1	1	1	1	1	1	1	1	1	1	1	1	1	1	1
1	100	0	1	100	0	0	10	0.891	0.453	1	0.902	0.701	1	1	1	1	1	1	1	0.303	0.696	NA
1	500	0	1	100	0	0	10	0.957	0.564	1	1	1	1	1	1	1	1	1	1	0.539	0.96	1
1	100	0.1	1	100	0	0	10	1	1	1	1	1	1	1	1	1	1	1	1	1	1	NA
1	500	0.1	1	100	0	0	10	1	1	1	1	1	1	1	1	1	1	1	1	1	1	1
1	100	0.3	1	100	0	0	10	1	1	1	1	1	1	1	1	1	1	1	1	1	1	NA
1	500	0.3	1	100	0	0	10	1	1	1	1	1	1	1	1	1	1	1	1	1	1	1
1	100	0	1	500	0	0	10	0.503	0.156	0.835	0.383	0.233	0.99	0.996	0.998	0.999	0.995	0.91	0.864	0.149	0.355	NA
1	500	0	1	500	0	0	10	0.591	0.151	1	0.92	0.767	1	1	1	1	1	1	1	0.346	0.738	NA
1	100	0.1	1	500	0	0	10	1	1	1	1	1	1	1	1	1	1	1	1	1	1	NA
1	500	0.1	1	500	0	0	10	1	1	1	1	1	1	1	1	1	1	1	1	1	1	NA
1	100	0.3	1	500	0	0	10	1	1	1	1	1	1	1	1	1	1	1	1	1	1	NA
1	500	0.3	1	500	0	0	10	1	1	1	1	1	1	1	1	1	1	1	1	1	1	NA
1	100	0	3	10	0	0	10	1	1	1	1	1	1	1	1	1	1	1	1	0.618	0.988	1
1	500	0	3	10	0	0	10	1	1	1	1	1	1	1	1	1	1	1	1	0.807	1	1
1	100	0.1	3	10	0	0	10	1	1	1	1	1	1	1	1	1	1	1	1	0.999	1	1

1	500	0.1	3	10	0	0	10	1	1	1	1	1	1	1	1	1	1	1	1	1	1	1
1	100	0.3	3	10	0	0	10	1	1	1	1	1	1	1	1	1	1	1	1	1	1	1
1	500	0.3	3	10	0	0	10	1	1	1	1	1	1	1	1	1	1	1	1	1	1	1
1	100	0	3	50	0	0	10	0.988	0.872	1	0.993	0.941	1	1	1	1	0.999	1	1	0.403	0.829	1
1	500	0	3	50	0	0	10	0.993	1	1	1	1	1	1	1	1	1	1	1	0.604	0.986	1
1	100	0.1	3	50	0	0	10	1	1	1	1	1	1	1	1	1	1	1	1	1	1	1
1	500	0.1	3	50	0	0	10	1	1	1	1	1	1	1	1	1	1	1	1	1	1	1
1	100	0.3	3	50	0	0	10	1	1	1	1	1	1	1	1	1	1	1	1	1	1	1
1	500	0.3	3	50	0	0	10	1	1	1	1	1	1	1	1	1	1	1	1	1	1	1
1	100	0	3	100	0	0	10	0.881	0.45	0.999	0.878	0.649	1	1	1	1	1	1	1	0.281	0.66	NA
1	500	0	3	100	0	0	10	0.957	0.557	1	1	1	1	1	1	1	1	1	1	0.524	0.951	1
1	100	0.1	3	100	0	0	10	1	1	1	1	1	1	1	1	1	1	1	1	1	1	NA
1	500	0.1	3	100	0	0	10	1	1	1	1	1	1	1	1	1	1	1	1	1	1	1
1	100	0.3	3	100	0	0	10	1	1	1	1	1	1	1	1	1	1	1	1	1	1	NA
1	500	0.3	3	100	0	0	10	1	1	1	1	1	1	1	1	1	1	1	1	1	1	1
1	100	0	3	500	0	0	10	0.481	0.156	0.778	0.355	0.224	0.981	0.989	0.994	0.996	0.983	0.872	0.789	0.135	0.32	NA
1	500	0	3	500	0	0	10	0.596	0.146	1	0.907	0.699	1	1	1	1	1	1	1	0.318	0.71	NA
1	100	0.1	3	500	0	0	10	1	1	1	1	1	1	1	1	1	1	1	1	1	1	NA
1	500	0.1	3	500	0	0	10	1	1	1	1	1	1	1	1	1	1	1	1	1	1	NA
1	100	0.3	3	500	0	0	10	1	1	1	1	1	1	1	1	1	1	1	1	1	1	NA
1	500	0.3	3	500	0	0	10	1	1	1	1	1	1	1	1	1	1	1	1	1	1	NA
1	100	0	6	10	0	0	10	1	1	1	1	1	1	1	1	1	0.999	1	0.999	0.578	0.978	1
1	500	0	6	10	0	0	10	1	1	1	1	1	1	1	1	1	1	1	1	0.784	1	1
1	100	0.1	6	10	0	0	10	1	1	1	1	1	1	1	1	1	1	1	1	0.999	1	1
1	500	0.1	6	10	0	0	10	1	1	1	1	1	1	1	1	1	1	1	1	1	1	1
1	100	0.3	6	10	0	0	10	1	1	1	1	1	1	1	1	1	1	1	1	1	1	1
1	500	0.3	6	10	0	0	10	1	1	1	1	1	1	1	1	1	1	1	1	1	1	1
1	100	0	6	50	0	0	10	0.965	0.764	1	0.961	0.852	1	1	1	0.999	0.989	1	0.999	0.35	0.753	1
1	500	0	6	50	0	0	10	0.994	1	1	1	1	1	1	1	1	1	1	1	0.569	0.978	1
1	100	0.1	6	50	0	0	10	1	1	1	1	1	1	1	1	1	1	1	1	1	1	1
1	500	0.1	6	50	0	0	10	1	1	1	1	1	1	1	1	1	1	1	1	1	1	1
1	100	0.3	6	50	0	0	10	1	1	1	1	1	1	1	1	1	1	1	1	1	1	1
1	500	0.3	6	50	0	0	10	1	1	1	1	1	1	1	1	1	1	1	1	1	1	1
1	100	0	6	100	0	0	10	0.84	0.408	0.989	0.78	0.545	0.999	0.999	0.999	0.999	0.976	0.994	0.99	0.244	0.564	NA
1	500	0	6	100	0	0	10	0.954	0.581	1	1	1	1	1	1	1	1	1	1	0.466	0.929	1
1	100	0.1	6	100	0	0	10	1	1	1	1	1	1	1	1	1	1	1	1	1	1	NA
1	500	0.1	6	100	0	0	10	1	1	1	1	1	1	1	1	1	1	1	1	1	1	1
1	100	0.3	6	100	0	0	10	1	1	1	1	1	1	1	1	1	1	1	1	1	1	NA
1	500	0.3	6	100	0	0	10	1	1	1	1	1	1	1	1	1	1	1	1	1	1	1
1	100	0	6	500	0	0	10	0.415	0.147	0.609	0.288	0.184	0.875	0.915	0.94	0.948	0.886	0.699	0.59	0.123	0.253	NA

1	500	0	6	500	0	0	10	0.584	0.14	1	0.824	0.55	1	1	1	1	1	1	1	0.272	0.613	NA
1	100	0.1	6	500	0	0	10	1	1	1	1	1	1	1	1	1	1	1	0.997	1	1	NA
1	500	0.1	6	500	0	0	10	1	1	1	1	1	1	1	1	1	1	1	1	1	1	NA
1	100	0.3	6	500	0	0	10	1	1	1	1	1	1	1	1	1	1	1	1	1	1	NA
1	500	0.3	6	500	0	0	10	1	1	1	1	1	1	1	1	1	1	1	1	1	1	NA
1	100	0	1	50	0	0	25	1	1	1	1	1	1	1	1	0.999	0.979	1	1	0.42	0.844	1
1	500	0	1	50	0	0	25	1	1	1	1	1	1	1	1	1	1	1	1	0.628	0.989	1
1	100	0.1	1	50	0	0	25	1	1	1	1	1	1	1	1	1	1	1	1	1	1	1
1	500	0.1	1	50	0	0	25	1	1	1	1	1	1	1	1	1	1	1	1	1	1	1
1	100	0.3	1	50	0	0	25	1	1	1	1	1	1	1	1	1	1	1	1	1	1	1
1	500	0.3	1	50	0	0	25	1	1	1	1	1	1	1	1	1	1	1	1	1	1	1
1	100	0	1	100	0	0	25	1	0.973	1	0.998	0.959	1	1	0.999	0.995	0.95	1	0.999	0.312	0.715	NA
1	500	0	1	100	0	0	25	1	1	1	1	1	1	1	1	1	1	1	1	0.552	0.951	1
1	100	0.1	1	100	0	0	25	1	1	1	1	1	1	1	1	1	1	1	1	1	1	NA
1	500	0.1	1	100	0	0	25	1	1	1	1	1	1	1	1	1	1	1	1	1	1	1
1	100	0.3	1	100	0	0	25	1	1	1	1	1	1	1	1	1	1	1	1	1	1	NA
1	500	0.3	1	100	0	0	25	1	1	1	1	1	1	1	1	1	1	1	1	1	1	1
1	100	0	1	500	0	0	25	0.757	0.326	0.865	0.548	0.366	0.961	0.966	0.968	0.936	0.784	0.904	0.873	0.146	0.329	NA
1	500	0	1	500	0	0	25	0.994	0.73	1	1	0.966	1	1	1	1	1	1	1	0.332	0.732	NA
1	100	0.1	1	500	0	0	25	1	1	1	1	1	1	1	1	1	1	1	1	1	1	NA
1	500	0.1	1	500	0	0	25	1	1	1	1	1	1	1	1	1	1	1	1	1	1	NA
1	100	0.3	1	500	0	0	25	1	1	1	1	1	1	1	1	1	1	1	1	1	1	NA
1	500	0.3	1	500	0	0	25	1	1	1	1	1	1	1	1	1	1	1	1	1	1	NA
1	100	0	3	50	0	0	25	1	1	1	1	1	1	1	1	0.999	0.971	1	1	0.412	0.849	1
1	500	0	3	50	0	0	25	1	1	1	1	1	1	1	1	1	1	1	1	0.625	0.986	1
1	100	0.1	3	50	0	0	25	1	1	1	1	1	1	1	1	1	1	1	1	1	1	1
1	500	0.1	3	50	0	0	25	1	1	1	1	1	1	1	1	1	1	1	1	1	1	1
1	100	0.3	3	50	0	0	25	1	1	1	1	1	1	1	1	1	1	1	1	1	1	1
1	500	0.3	3	50	0	0	25	1	1	1	1	1	1	1	1	1	1	1	1	1	1	1
1	100	0	3	100	0	0	25	0.998	0.959	1	0.996	0.951	1	1	0.999	0.992	0.937	1	0.999	0.301	0.699	NA
1	500	0	3	100	0	0	25	1	1	1	1	1	1	1	1	1	1	1	1	0.546	0.951	1
1	100	0.1	3	100	0	0	25	1	1	1	1	1	1	1	1	1	1	1	1	1	1	NA
1	500	0.1	3	100	0	0	25	1	1	1	1	1	1	1	1	1	1	1	1	1	1	1
1	100	0.3	3	100	0	0	25	1	1	1	1	1	1	1	1	1	1	1	1	1	1	NA
1	500	0.3	3	100	0	0	25	1	1	1	1	1	1	1	1	1	1	1	1	1	1	1
1	100	0	3	500	0	0	25	0.732	0.33	0.847	0.533	0.357	0.945	0.956	0.957	0.921	0.753	0.888	0.828	0.141	0.325	NA
1	500	0	3	500	0	0	25	0.995	0.724	1	0.998	0.964	1	1	1	1	1	1	1	0.318	0.716	NA
1	100	0.1	3	500	0	0	25	1	1	1	1	1	1	1	1	1	1	1	1	1	1	NA
1	500	0.1	3	500	0	0	25	1	1	1	1	1	1	1	1	1	1	1	1	1	1	NA
1	100	0.3	3	500	0	0	25	1	1	1	1	1	1	1	1	1	1	1	1	1	1	NA

1	500	0.3	3	500	0	0	25	1	1	1	1	1	1	1	1	1	1	1	1	1	1	NA
1	100	0	6	50	0	0	25	1	0.999	1	1	0.999	1	1	1	0.997	0.932	1	1	0.388	0.815	1
1	500	0	6	50	0	0	25	1	1	1	1	1	1	1	1	1	1	1	1	0.595	0.983	1
1	100	0.1	6	50	0	0	25	1	1	1	1	1	1	1	1	1	1	1	1	1	1	1
1	500	0.1	6	50	0	0	25	1	1	1	1	1	1	1	1	1	1	1	1	1	1	1
1	100	0.3	6	50	0	0	25	1	1	1	1	1	1	1	1	1	1	1	1	1	1	1
1	500	0.3	6	50	0	0	25	1	1	1	1	1	1	1	1	1	1	1	1	1	1	1
1	100	0	6	100	0	0	25	0.995	0.929	1	0.98	0.899	1	1	0.995	0.985	0.87	1	0.998	0.283	0.652	NA
1	500	0	6	100	0	0	25	1	1	1	1	1	1	1	1	1	1	1	1	0.531	0.939	1
1	100	0.1	6	100	0	0	25	1	1	1	1	1	1	1	1	1	1	1	1	1	1	NA
1	500	0.1	6	100	0	0	25	1	1	1	1	1	1	1	1	1	1	1	1	1	1	1
1	100	0.3	6	100	0	0	25	1	1	1	1	1	1	1	1	1	1	1	1	1	1	NA
1	500	0.3	6	100	0	0	25	1	1	1	1	1	1	1	1	1	1	1	1	1	1	1
1	100	0	6	500	0	0	25	0.669	0.302	0.762	0.508	0.339	0.88	0.893	0.892	0.843	0.662	0.819	0.734	0.137	0.306	NA
1	500	0	6	500	0	0	25	0.993	0.719	1	0.998	0.95	1	1	1	1	1	1	1	0.287	0.682	NA
1	100	0.1	6	500	0	0	25	1	1	1	1	1	1	1	1	1	1	1	1	1	1	NA
1	500	0.1	6	500	0	0	25	1	1	1	1	1	1	1	1	1	1	1	1	1	1	NA
1	100	0.3	6	500	0	0	25	1	1	1	1	1	1	1	1	1	1	1	1	1	1	NA
1	500	0.3	6	500	0	0	25	1	1	1	1	1	1	1	1	1	1	1	1	1	1	NA
1	100	0	1	50	0	0	50	1	1	1	1	1	1	1	1	0.992	0.858	1	1	0.419	0.852	1
1	500	0	1	50	0	0	50	1	1	1	1	1	1	1	1	1	1	1	1	0.623	0.984	1
1	100	0.1	1	50	0	0	50	1	1	1	1	1	1	1	1	1	1	1	1	1	1	1
1	500	0.1	1	50	0	0	50	1	1	1	1	1	1	1	1	1	1	1	1	1	1	1
1	100	0.3	1	50	0	0	50	1	1	1	1	1	1	1	1	1	1	1	1	1	1	1
1	500	0.3	1	50	0	0	50	1	1	1	1	1	1	1	1	1	1	1	1	1	1	1
1	100	0	1	100	0	0	50	1	0.996	1	0.999	0.993	1	0.999	0.996	0.977	0.772	1	1	0.304	0.69	NA
1	500	0	1	100	0	0	50	1	1	1	1	1	1	1	1	1	1	1	1	0.544	0.962	1
1	100	0.1	1	100	0	0	50	1	1	1	1	1	1	1	1	1	1	1	1	1	1	NA
1	500	0.1	1	100	0	0	50	1	1	1	1	1	1	1	1	1	1	1	1	1	1	1
1	100	0.3	1	100	0	0	50	1	1	1	1	1	1	1	1	1	1	1	1	1	1	NA
1	500	0.3	1	100	0	0	50	1	1	1	1	1	1	1	1	1	1	1	1	1	1	1
1	100	0	1	500	0	0	50	0.809	0.484	0.861	0.673	0.511	0.916	0.911	0.88	0.771	0.477	0.896	0.871	0.177	0.327	NA
1	500	0	1	500	0	0	50	1	1	1	1	0.999	1	1	1	1	1	1	1	0.322	0.71	NA
1	100	0.1	1	500	0	0	50	1	1	1	1	1	1	1	1	1	1	1	1	1	1	NA
1	500	0.1	1	500	0	0	50	1	1	1	1	1	1	1	1	1	1	1	1	1	1	NA
1	100	0.3	1	500	0	0	50	1	1	1	1	1	1	1	1	1	1	1	1	1	1	NA
1	500	0.3	1	500	0	0	50	1	1	1	1	1	1	1	1	1	1	1	1	1	1	NA
1	100	0	3	50	0	0	50	1	1	1	1	1	1	1	1	0.992	0.85	1	1	0.42	0.848	1
1	500	0	3	50	0	0	50	1	1	1	1	1	1	1	1	1	1	1	1	0.618	0.982	1
1	100	0.1	3	50	0	0	50	1	1	1	1	1	1	1	1	1	1	1	1	1	1	1

1	500	0.1	3	50	0	0	50	1	1	1	1	1	1	1	1	1	1	1	1	1	1	1
1	100	0.3	3	50	0	0	50	1	1	1	1	1	1	1	1	1	1	1	1	1	1	1
1	500	0.3	3	50	0	0	50	1	1	1	1	1	1	1	1	1	1	1	1	1	1	1
1	100	0	3	100	0	0	50	1	0.994	1	0.999	0.995	1	1	0.996	0.974	0.769	1	1	0.291	0.686	NA
1	500	0	3	100	0	0	50	1	1	1	1	1	1	1	1	1	1	1	1	0.546	0.958	1
1	100	0.1	3	100	0	0	50	1	1	1	1	1	1	1	1	1	1	1	1	1	1	NA
1	500	0.1	3	100	0	0	50	1	1	1	1	1	1	1	1	1	1	1	1	1	1	1
1	100	0.3	3	100	0	0	50	1	1	1	1	1	1	1	1	1	1	1	1	1	1	NA
1	500	0.3	3	100	0	0	50	1	1	1	1	1	1	1	1	1	1	1	1	1	1	1
1	100	0	3	500	0	0	50	0.804	0.496	0.851	0.674	0.48	0.908	0.9	0.875	0.748	0.484	0.883	0.855	0.169	0.335	NA
1	500	0	3	500	0	0	50	1	1	1	1	1	1	1	1	1	1	1	1	0.323	0.7	NA
1	100	0.1	3	500	0	0	50	1	1	1	1	1	1	1	1	1	1	1	1	1	1	NA
1	500	0.1	3	500	0	0	50	1	1	1	1	1	1	1	1	1	1	1	1	1	1	NA
1	100	0.3	3	500	0	0	50	1	1	1	1	1	1	1	1	1	1	1	1	1	1	NA
1	500	0.3	3	500	0	0	50	1	1	1	1	1	1	1	1	1	1	1	1	1	1	NA
1	100	0	6	50	0	0	50	1	1	1	1	0.999	1	1	1	0.983	0.834	1	1	0.397	0.834	1
1	500	0	6	50	0	0	50	1	1	1	1	1	1	1	1	1	1	1	1	0.602	0.982	1
1	100	0.1	6	50	0	0	50	1	1	1	1	1	1	1	1	1	1	1	1	1	1	1
1	500	0.1	6	50	0	0	50	1	1	1	1	1	1	1	1	1	1	1	1	1	1	1
1	100	0.3	6	50	0	0	50	1	1	1	1	1	1	1	1	1	1	1	1	1	1	1
1	500	0.3	6	50	0	0	50	1	1	1	1	1	1	1	1	1	1	1	1	1	1	1
1	100	0	6	100	0	0	50	1	0.991	1	0.998	0.989	1	0.999	0.994	0.963	0.723	1	1	0.277	0.665	NA
1	500	0	6	100	0	0	50	1	1	1	1	1	1	1	1	1	1	1	1	0.531	0.95	1
1	100	0.1	6	100	0	0	50	1	1	1	1	1	1	1	1	1	1	1	1	1	1	NA
1	500	0.1	6	100	0	0	50	1	1	1	1	1	1	1	1	1	1	1	1	1	1	1
1	100	0.3	6	100	0	0	50	1	1	1	1	1	1	1	1	1	1	1	1	1	1	NA
1	500	0.3	6	100	0	0	50	1	1	1	1	1	1	1	1	1	1	1	1	1	1	1
1	100	0	6	500	0	0	50	0.77	0.456	0.819	0.627	0.459	0.868	0.861	0.823	0.704	0.453	0.845	0.813	0.156	0.321	NA
1	500	0	6	500	0	0	50	1	1	1	1	0.999	1	1	1	1	1	1	1	0.306	0.696	NA
1	100	0.1	6	500	0	0	50	1	1	1	1	1	1	1	1	1	1	1	1	1	1	NA
1	500	0.1	6	500	0	0	50	1	1	1	1	1	1	1	1	1	1	1	1	1	1	NA
1	100	0.3	6	500	0	0	50	1	1	1	1	1	1	1	1	1	1	1	1	1	1	NA
1	500	0.3	6	500	0	0	50	1	1	1	1	1	1	1	1	1	1	1	1	1	1	NA
1	100	0	1	10	0	1	0	0.191	0.105	1	0.994	1	1	1	1	1	1	1	1	0.684	1	1
1	500	0	1	10	0	1	0	0.194	0.095	1	1	1	1	1	1	1	1	1	1	0.836	1	1
1	100	0.1	1	10	0	1	0	0.623	0.446	1	0.996	1	1	1	1	1	1	1	1	0.889	1	1
1	500	0.1	1	10	0	1	0	0.992	0.979	1	1	1	1	1	1	1	1	1	1	1	1	1
1	100	0.3	1	10	0	1	0	1	0.997	1	1	1	1	1	1	1	1	1	1	1	1	1
1	500	0.3	1	10	0	1	0	1	1	1	1	1	1	1	1	1	1	1	1	1	1	1
1	100	0	1	50	0	1	0	0.119	0.058	1	0.567	0.647	1	1	1	1	1	1	1	0.39	0.828	1

1	500	0	1	50	0	1	0	0.115	0.058	1	0.994	1	1	1	1	1	1	1	1	0.603	0.987	1
1	100	0.1	1	50	0	1	0	0.746	0.65	1	0.917	0.932	1	1	1	1	1	1	1	0.85	0.957	1
1	500	0.1	1	50	0	1	0	1	1	1	1	1	1	1	1	1	1	1	1	1	1	1
1	100	0.3	1	50	0	1	0	1	1	1	1	1	1	1	1	1	1	1	1	1	1	1
1	500	0.3	1	50	0	1	0	1	1	1	1	1	1	1	1	1	1	1	1	1	1	1
1	100	0	1	100	0	1	0	0.11	0.077	1	0.372	0.362	1	1	1	1	1	1	1	0.311	0.658	NA
1	500	0	1	100	0	1	0	0.089	0.053	1	0.875	1	1	1	1	1	1	1	1	0.503	0.955	1
1	100	0.1	1	100	0	1	0	0.794	0.734	1	0.89	0.878	1	1	1	1	1	1	1	0.844	0.912	NA
1	500	0.1	1	100	0	1	0	1	0.999	1	1	1	1	1	1	1	1	1	1	1	1	1
1	100	0.3	1	100	0	1	0	0.999	0.999	1	0.999	0.999	1	1	1	1	1	1	1	0.999	1	NA
1	500	0.3	1	100	0	1	0	1	1	1	1	1	1	1	1	1	1	1	1	1	1	1
1	100	0	1	500	0	1	0	0.1	0.06	0.98	0.153	0.133	1	1	1	1	1	0.817	0.713	0.175	0.306	NA
1	500	0	1	500	0	1	0	0.078	0.054	1	0.366	0.679	1	1	1	1	1	1	1	0.298	0.652	NA
1	100	0.1	1	500	0	1	0	0.812	0.799	0.948	0.826	0.817	1	1	1	1	1	0.907	0.819	0.82	0.727	NA
1	500	0.1	1	500	0	1	0	1	1	1	1	1	1	1	1	1	1	1	1	1	1	NA
1	100	0.3	1	500	0	1	0	1	1	1	1	1	1	1	1	1	1	1	0.974	1	0.997	NA
1	500	0.3	1	500	0	1	0	1	1	1	1	1	1	1	1	1	1	1	1	1	1	NA
1	100	0	3	10	0	1	0	0.219	0.108	0.995	0.584	0.55	1	1	1	1	1	0.998	0.993	0.471	0.965	0.998
1	500	0	3	10	0	1	0	0.207	0.105	1	0.994	1	1	1	1	1	1	1	1	0.729	1	1
1	100	0.1	3	10	0	1	0	0.37	0.238	0.997	0.734	0.675	0.999	1	1	1	1	0.999	0.988	0.658	0.97	0.998
1	500	0.1	3	10	0	1	0	0.827	0.708	1	1	1	1	1	1	1	1	1	1	0.997	1	1
1	100	0.3	3	10	0	1	0	0.876	0.822	0.996	0.954	0.935	1	1	1	1	1	0.998	0.994	0.955	0.988	0.999
1	500	0.3	3	10	0	1	0	1	1	1	1	1	1	1	1	1	1	1	1	1	1	1
1	100	0	3	50	0	1	0	0.134	0.067	0.799	0.218	0.148	0.989	0.994	0.999	1	1	0.868	0.816	0.213	0.446	0.715
1	500	0	3	50	0	1	0	0.113	0.066	1	0.583	0.746	1	1	1	1	1	1	1	0.425	0.886	1
1	100	0.1	3	50	0	1	0	0.393	0.294	0.823	0.466	0.402	0.987	0.994	0.997	0.998	1	0.864	0.824	0.473	0.6	0.735
1	500	0.1	3	50	0	1	0	0.94	0.911	1	0.989	0.995	1	1	1	1	1	1	1	0.993	0.994	1
1	100	0.3	3	50	0	1	0	0.902	0.882	0.946	0.912	0.897	0.982	0.989	1	1	1	0.951	0.876	0.917	0.872	0.725
1	500	0.3	3	50	0	1	0	1	1	1	1	1	1	1	1	1	1	1	1	1	1	1
1	100	0	3	100	0	1	0	0.112	0.066	0.595	0.15	0.1	0.948	0.979	0.994	0.998	0.999	0.648	0.546	0.17	0.295	NA
1	500	0	3	100	0	1	0	0.107	0.066	1	0.402	0.43	1	1	1	1	1	1	1	0.323	0.73	1
1	100	0.1	3	100	0	1	0	0.398	0.33	0.634	0.421	0.369	0.912	0.961	0.991	0.996	0.997	0.668	0.582	0.439	0.459	NA
1	500	0.1	3	100	0	1	0	0.969	0.958	1	0.984	0.982	1	1	1	1	1	1	1	0.984	0.986	1
1	100	0.3	3	100	0	1	0	0.872	0.857	0.904	0.879	0.866	0.949	0.96	0.973	0.993	0.997	0.908	0.693	0.877	0.759	NA
1	500	0.3	3	100	0	1	0	1	1	1	1	1	1	1	1	1	1	1	1	1	1	1
1	100	0	3	500	0	1	0	0.093	0.049	0.231	0.088	0.072	0.6	0.732	0.863	0.956	0.991	0.263	0.193	0.09	0.154	NA
1	500	0	3	500	0	1	0	0.087	0.053	0.99	0.141	0.127	1	1	1	1	1	0.997	0.993	0.152	0.332	NA
1	100	0.1	3	500	0	1	0	0.431	0.418	0.476	0.437	0.428	0.551	0.607	0.718	0.904	0.989	0.474	0.22	0.43	0.314	NA
1	500	0.1	3	500	0	1	0	0.974	0.971	0.996	0.981	0.98	1	1	1	1	1	0.997	0.997	0.979	0.949	NA
1	100	0.3	3	500	0	1	0	0.883	0.883	0.892	0.882	0.881	0.903	0.909	0.918	0.939	0.993	0.893	0.318	0.882	0.716	NA

1	500	0.3	3	500	0	1	0	1	1	1	1	1	1	1	1	1	1	1	1	1	1	NA
1	100	0	6	10	0	1	0	0.189	0.121	0.517	0.257	0.196	0.655	0.675	0.685	0.697	0.698	0.57	0.522	0.231	0.438	0.547
1	500	0	6	10	0	1	0	0.202	0.114	1	0.75	0.795	1	1	1	1	1	1	0.999	0.551	0.98	1
1	100	0.1	6	10	0	1	0	0.227	0.147	0.512	0.295	0.219	0.679	0.696	0.707	0.71	0.715	0.582	0.521	0.3	0.475	0.55
1	500	0.1	6	10	0	1	0	0.467	0.312	1	0.837	0.844	1	1	1	1	1	1	0.998	0.841	0.987	1
1	100	0.3	6	10	0	1	0	0.459	0.386	0.608	0.493	0.443	0.678	0.706	0.721	0.725	0.728	0.622	0.53	0.511	0.514	0.536
1	500	0.3	6	10	0	1	0	0.968	0.934	1	0.99	0.981	1	1	1	1	1	1	1	0.988	0.997	1
1	100	0	6	50	0	1	0	0.111	0.059	0.216	0.117	0.09	0.377	0.435	0.463	0.503	0.521	0.239	0.219	0.109	0.159	0.163
1	500	0	6	50	0	1	0	0.115	0.069	0.95	0.26	0.176	1	1	1	1	1	0.991	0.97	0.201	0.495	0.986
1	100	0.1	6	50	0	1	0	0.18	0.151	0.252	0.187	0.154	0.371	0.415	0.476	0.529	0.539	0.266	0.23	0.196	0.21	0.17
1	500	0.1	6	50	0	1	0	0.524	0.429	0.956	0.632	0.55	1	1	1	1	1	0.988	0.961	0.667	0.713	0.984
1	100	0.3	6	50	0	1	0	0.414	0.395	0.44	0.415	0.405	0.472	0.492	0.533	0.58	0.597	0.447	0.239	0.412	0.304	0.162
1	500	0.3	6	50	0	1	0	0.977	0.972	0.994	0.981	0.976	0.998	1	1	1	1	0.995	0.982	0.983	0.96	0.981
1	100	0	6	100	0	1	0	0.094	0.06	0.145	0.091	0.08	0.25	0.293	0.342	0.385	0.407	0.163	0.149	0.083	0.113	NA
1	500	0	6	100	0	1	0	0.11	0.067	0.824	0.192	0.125	0.993	0.996	0.999	0.999	1	0.917	0.885	0.141	0.305	0.883
1	100	0.1	6	100	0	1	0	0.162	0.139	0.197	0.162	0.151	0.264	0.305	0.358	0.406	0.438	0.216	0.142	0.179	0.162	NA
1	500	0.1	6	100	0	1	0	0.606	0.532	0.874	0.636	0.597	0.989	0.994	0.998	1	1	0.92	0.889	0.664	0.592	0.902
1	100	0.3	6	100	0	1	0	0.384	0.376	0.396	0.388	0.374	0.418	0.425	0.446	0.497	0.538	0.387	0.186	0.379	0.278	NA
1	500	0.3	6	100	0	1	0	0.975	0.973	0.985	0.975	0.973	0.992	0.993	0.997	1	1	0.987	0.919	0.979	0.937	0.897
1	100	0	6	500	0	1	0	0.081	0.05	0.092	0.064	0.055	0.138	0.152	0.178	0.226	0.287	0.101	0.088	0.056	0.08	NA
1	500	0	6	500	0	1	0	0.087	0.058	0.327	0.087	0.07	0.813	0.916	0.975	0.996	0.998	0.437	0.342	0.077	0.121	NA
1	100	0.1	6	500	0	1	0	0.18	0.175	0.185	0.182	0.177	0.199	0.206	0.218	0.237	0.317	0.189	0.092	0.185	0.117	NA
1	500	0.1	6	500	0	1	0	0.624	0.604	0.674	0.635	0.626	0.781	0.835	0.918	0.986	0.999	0.695	0.384	0.634	0.449	NA
1	100	0.3	6	500	0	1	0	0.418	0.421	0.419	0.416	0.42	0.423	0.425	0.424	0.438	0.47	0.426	0.113	0.418	0.239	NA
1	500	0.3	6	500	0	1	0	0.975	0.974	0.976	0.975	0.973	0.979	0.98	0.984	0.993	0.999	0.974	0.484	0.971	0.888	NA
1	100	0	1	10	0	2	0	0.454	0.204	1	0.998	1	1	1	1	1	1	1	1	0.64	0.996	1
1	500	0	1	10	0	2	0	0.459	0.193	1	1	1	1	1	1	1	1	1	1	0.813	1	1
1	100	0.1	1	10	0	2	0	0.935	0.814	1	1	1	1	1	1	1	1	1	1	0.976	1	1
1	500	0.1	1	10	0	2	0	1	1	1	1	1	1	1	1	1	1	1	1	1	1	1
1	100	0.3	1	10	0	2	0	1	1	1	1	1	1	1	1	1	1	1	1	1	1	1
1	500	0.3	1	10	0	2	0	1	1	1	1	1	1	1	1	1	1	1	1	1	1	1
1	100	0	1	50	0	2	0	0.204	0.078	1	0.66	0.557	1	1	1	1	1	1	1	0.382	0.841	1
1	500	0	1	50	0	2	0	0.22	0.095	1	0.998	1	1	1	1	1	1	1	1	0.607	0.984	1
1	100	0.1	1	50	0	2	0	0.979	0.948	1	0.996	0.995	1	1	1	1	1	1	1	0.988	0.996	1
1	500	0.1	1	50	0	2	0	1	1	1	1	1	1	1	1	1	1	1	1	1	1	1
1	100	0.3	1	50	0	2	0	1	1	1	1	1	1	1	1	1	1	1	1	1	1	1
1	500	0.3	1	50	0	2	0	1	1	1	1	1	1	1	1	1	1	1	1	1	1	1
1	100	0	1	100	0	2	0	0.193	0.094	1	0.459	0.321	1	1	1	1	1	1	1	0.323	0.66	NA
1	500	0	1	100	0	2	0	0.175	0.065	1	0.962	1	1	1	1	1	1	1	1	0.548	0.964	1
1	100	0.1	1	100	0	2	0	0.982	0.967	1	0.994	0.986	1	1	1	1	1	1	1	0.993	0.991	NA

1	500	0.1	1	100	0	2	0	1	1	1	1	1	1	1	1	1	1	1	1	1	1	1
1	100	0.3	1	100	0	2	0	1	1	1	1	1	1	1	1	1	1	1	1	1	1	NA
1	500	0.3	1	100	0	2	0	1	1	1	1	1	1	1	1	1	1	1	1	1	1	1
1	100	0	1	500	0	2	0	0.129	0.079	0.847	0.192	0.137	0.999	1	1	1	1	0.853	0.794	0.172	0.321	NA
1	500	0	1	500	0	2	0	0.115	0.053	1	0.47	0.534	1	1	1	1	1	1	1	0.309	0.701	NA
1	100	0.1	1	500	0	2	0	0.99	0.987	0.997	0.99	0.989	1	1	1	1	1	0.997	0.958	0.991	0.963	NA
1	500	0.1	1	500	0	2	0	1	1	1	1	1	1	1	1	1	1	1	1	1	1	NA
1	100	0.3	1	500	0	2	0	1	1	1	1	1	1	1	1	1	1	1	1	1	1	NA
1	500	0.3	1	500	0	2	0	1	1	1	1	1	1	1	1	1	1	1	1	1	1	NA
1	100	0	3	10	0	2	0	0.47	0.202	1	0.943	0.936	1	1	1	1	1	1	0.997	0.535	0.969	1
1	500	0	3	10	0	2	0	0.467	0.208	1	1	1	1	1	1	1	1	1	1	0.737	1	1
1	100	0.1	3	10	0	2	0	0.829	0.625	1	0.991	0.992	1	1	1	1	1	1	0.999	0.943	0.999	1
1	500	0.1	3	10	0	2	0	1	0.996	1	1	1	1	1	1	1	1	1	1	1	1	1
1	100	0.3	3	10	0	2	0	1	0.998	1	1	1	1	1	1	1	1	1	1	1	1	1
1	500	0.3	3	10	0	2	0	1	1	1	1	1	1	1	1	1	1	1	1	1	1	1
1	100	0	3	50	0	2	0	0.235	0.102	0.959	0.388	0.234	1	1	1	1	1	0.985	0.97	0.258	0.597	0.981
1	500	0	3	50	0	2	0	0.222	0.094	1	0.952	0.992	1	1	1	1	1	1	1	0.493	0.932	1
1	100	0.1	3	50	0	2	0	0.842	0.733	0.995	0.912	0.832	1	1	1	1	1	0.998	0.991	0.901	0.92	0.984
1	500	0.1	3	50	0	2	0	1	1	1	1	1	1	1	1	1	1	1	1	1	1	1
1	100	0.3	3	50	0	2	0	1	1	1	1	1	1	1	1	1	1	1	1	1	0.999	0.998
1	500	0.3	3	50	0	2	0	1	1	1	1	1	1	1	1	1	1	1	1	1	1	1
1	100	0	3	100	0	2	0	0.197	0.082	0.847	0.256	0.146	0.995	0.996	0.998	0.998	0.998	0.908	0.866	0.227	0.424	NA
1	500	0	3	100	0	2	0	0.189	0.074	1	0.751	0.79	1	1	1	1	1	1	1	0.39	0.87	1
1	100	0.1	3	100	0	2	0	0.865	0.789	0.979	0.882	0.844	0.999	1	1	1	1	0.987	0.948	0.885	0.851	NA
1	500	0.1	3	100	0	2	0	1	0.999	1	1	1	1	1	1	1	1	1	1	1	1	1
1	100	0.3	3	100	0	2	0	0.999	0.999	1	1	0.999	1	1	1	1	1	1	0.995	0.999	1	NA
1	500	0.3	3	100	0	2	0	1	1	1	1	1	1	1	1	1	1	1	1	1	1	1
1	100	0	3	500	0	2	0	0.13	0.08	0.388	0.136	0.097	0.803	0.9	0.967	0.987	0.995	0.448	0.34	0.114	0.189	NA
1	500	0	3	500	0	2	0	0.122	0.058	1	0.26	0.196	1	1	1	1	1	1	1	0.197	0.45	NA
1	100	0.1	3	500	0	2	0	0.872	0.859	0.903	0.878	0.868	0.953	0.969	0.988	0.999	1	0.914	0.474	0.871	0.722	NA
1	500	0.1	3	500	0	2	0	1	1	1	1	1	1	1	1	1	1	1	1	1	1	NA
1	100	0.3	3	500	0	2	0	1	1	1	1	1	1	1	1	1	1	1	0.813	1	0.999	NA
1	500	0.3	3	500	0	2	0	1	1	1	1	1	1	1	1	1	1	1	1	1	1	NA
1	100	0	6	10	0	2	0	0.43	0.213	0.854	0.578	0.448	0.904	0.904	0.898	0.886	0.864	0.881	0.834	0.345	0.706	0.904
1	500	0	6	10	0	2	0	0.485	0.213	1	0.997	0.999	1	1	1	1	1	1	1	0.612	0.987	1
1	100	0.1	6	10	0	2	0	0.613	0.384	0.934	0.757	0.659	0.956	0.956	0.951	0.946	0.937	0.944	0.888	0.736	0.853	0.921
1	500	0.1	6	10	0	2	0	0.953	0.845	1	1	1	1	1	1	1	1	1	1	1	1	1
1	100	0.3	6	10	0	2	0	0.949	0.909	0.992	0.976	0.952	0.991	0.991	0.991	0.99	0.989	0.993	0.972	0.972	0.959	0.965
1	500	0.3	6	10	0	2	0	1	1	1	1	1	1	1	1	1	1	1	1	1	1	1
1	100	0	6	50	0	2	0	0.202	0.087	0.43	0.206	0.142	0.639	0.691	0.72	0.729	0.697	0.47	0.449	0.137	0.243	0.344

1	500	0	6	50	0	2	0	0.22	0.092	1	0.624	0.439	1	1	1	1	1	1	1	0.321	0.706	1
1	100	0.1	6	50	0	2	0	0.505	0.38	0.67	0.511	0.43	0.803	0.829	0.845	0.85	0.83	0.692	0.537	0.513	0.483	0.371
1	500	0.1	6	50	0	2	0	0.988	0.96	1	0.999	0.995	1	1	1	1	1	1	1	0.999	0.996	1
1	100	0.3	6	50	0	2	0	0.931	0.913	0.943	0.935	0.923	0.966	0.973	0.976	0.98	0.977	0.954	0.702	0.94	0.836	0.466
1	500	0.3	6	50	0	2	0	1	1	1	1	1	1	1	1	1	1	1	1	1	1	1
1	100	0	6	100	0	2	0	0.158	0.076	0.291	0.138	0.094	0.478	0.533	0.573	0.608	0.594	0.34	0.278	0.098	0.186	NA
1	500	0	6	100	0	2	0	0.197	0.08	0.998	0.411	0.277	1	1	1	1	1	1	0.999	0.226	0.527	1
1	100	0.1	6	100	0	2	0	0.492	0.421	0.568	0.48	0.433	0.672	0.7	0.743	0.763	0.742	0.59	0.348	0.481	0.409	NA
1	500	0.1	6	100	0	2	0	0.984	0.974	0.998	0.987	0.985	1	1	1	1	1	1	1	0.989	0.984	1
1	100	0.3	6	100	0	2	0	0.916	0.909	0.933	0.915	0.909	0.942	0.951	0.958	0.96	0.947	0.93	0.526	0.922	0.763	NA
1	500	0.3	6	100	0	2	0	1	1	1	1	1	1	1	1	1	1	1	1	1	1	1
1	100	0	6	500	0	2	0	0.113	0.061	0.134	0.092	0.075	0.216	0.247	0.287	0.367	0.421	0.155	0.124	0.071	0.1	NA
1	500	0	6	500	0	2	0	0.113	0.063	0.683	0.129	0.082	0.989	0.998	0.999	1	1	0.853	0.754	0.098	0.193	NA
1	100	0.1	6	500	0	2	0	0.493	0.476	0.51	0.49	0.483	0.534	0.542	0.568	0.613	0.649	0.514	0.144	0.489	0.307	NA
1	500	0.1	6	500	0	2	0	0.99	0.99	0.995	0.991	0.988	0.998	1	1	1	1	0.995	0.87	0.992	0.958	NA
1	100	0.3	6	500	0	2	0	0.917	0.915	0.918	0.916	0.917	0.921	0.922	0.92	0.925	0.935	0.916	0.261	0.915	0.75	NA
1	500	0.3	6	500	0	2	0	1	1	1	1	1	1	1	1	1	1	1	0.987	1	1	NA
1	100	0	1	10	0	5	0	1	1	1	1	1	1	1	1	1	1	1	0.998	0.663	0.991	1
1	500	0	1	10	0	5	0	0.999	1	1	1	1	1	1	1	1	1	1	1	0.805	0.999	1
1	100	0.1	1	10	0	5	0	1	1	1	1	1	1	1	1	1	1	1	1	0.999	1	1
1	500	0.1	1	10	0	5	0	1	1	1	1	1	1	1	1	1	1	1	1	1	1	1
1	100	0.3	1	10	0	5	0	1	1	1	1	1	1	1	1	1	1	1	1	1	1	1
1	500	0.3	1	10	0	5	0	1	1	1	1	1	1	1	1	1	1	1	1	1	1	1
1	100	0	1	50	0	5	0	0.672	0.271	1	0.932	0.802	1	1	1	1	1	1	1	0.427	0.847	1
1	500	0	1	50	0	5	0	0.721	0.273	1	1	1	1	1	1	1	1	1	1	0.638	0.992	1
1	100	0.1	1	50	0	5	0	1	1	1	1	1	1	1	1	1	1	1	1	1	1	1
1	500	0.1	1	50	0	5	0	1	1	1	1	1	1	1	1	1	1	1	1	1	1	1
1	100	0.3	1	50	0	5	0	1	1	1	1	1	1	1	1	1	1	1	1	1	1	1
1	500	0.3	1	50	0	5	0	1	1	1	1	1	1	1	1	1	1	1	1	1	1	1
1	100	0	1	100	0	5	0	0.5	0.165	1	0.696	0.441	1	1	1	1	1	1	1	0.295	0.658	NA
1	500	0	1	100	0	5	0	0.534	0.178	1	1	1	1	1	1	1	1	1	1	0.549	0.956	1
1	100	0.1	1	100	0	5	0	1	1	1	1	1	1	1	1	1	1	1	1	1	1	NA
1	500	0.1	1	100	0	5	0	1	1	1	1	1	1	1	1	1	1	1	1	1	1	1
1	100	0.3	1	100	0	5	0	1	1	1	1	1	1	1	1	1	1	1	1	1	1	NA
1	500	0.3	1	100	0	5	0	1	1	1	1	1	1	1	1	1	1	1	1	1	1	1
1	100	0	1	500	0	5	0	0.272	0.091	0.81	0.264	0.151	0.996	0.999	1	1	1	0.888	0.845	0.151	0.324	NA
1	500	0	1	500	0	5	0	0.244	0.07	1	0.735	0.576	1	1	1	1	1	1	1	0.329	0.696	NA
1	100	0.1	1	500	0	5	0	1	1	1	1	1	1	1	1	1	1	1	0.999	1	1	NA
1	500	0.1	1	500	0	5	0	1	1	1	1	1	1	1	1	1	1	1	1	1	1	NA
1	100	0.3	1	500	0	5	0	1	1	1	1	1	1	1	1	1	1	1	1	1	1	NA

1	500	0.3	1	500	0	5	0	1	1	1	1	1	1	1	1	1	1	1	1	1	1	NA
1	100	0	3	10	0	5	0	0.999	1	1	1	1	1	1	1	1	1	1	0.997	0.604	0.972	1
1	500	0	3	10	0	5	0	1	1	1	1	1	1	1	1	1	1	1	1	0.771	0.999	1
1	100	0.1	3	10	0	5	0	1	1	1	1	1	1	1	1	1	1	1	1	0.999	1	1
1	500	0.1	3	10	0	5	0	1	1	1	1	1	1	1	1	1	1	1	1	1	1	1
1	100	0.3	3	10	0	5	0	1	1	1	1	1	1	1	1	1	1	1	1	1	1	1
1	500	0.3	3	10	0	5	0	1	1	1	1	1	1	1	1	1	1	1	1	1	1	1
1	100	0	3	50	0	5	0	0.673	0.252	1	0.828	0.6	1	1	1	1	1	1	1	0.363	0.744	1
1	500	0	3	50	0	5	0	0.704	0.259	1	1	1	1	1	1	1	1	1	1	0.58	0.977	1
1	100	0.1	3	50	0	5	0	1	1	1	1	1	1	1	1	1	1	1	1	1	1	1
1	500	0.1	3	50	0	5	0	1	1	1	1	1	1	1	1	1	1	1	1	1	1	1
1	100	0.3	3	50	0	5	0	1	1	1	1	1	1	1	1	1	1	1	1	1	1	1
1	500	0.3	3	50	0	5	0	1	1	1	1	1	1	1	1	1	1	1	1	1	1	1
1	100	0	3	100	0	5	0	0.503	0.164	0.979	0.56	0.336	1	1	1	1	1	0.993	0.992	0.253	0.522	NA
1	500	0	3	100	0	5	0	0.569	0.163	1	0.997	0.997	1	1	1	1	1	1	1	0.489	0.927	1
1	100	0.1	3	100	0	5	0	1	1	1	1	1	1	1	1	1	1	1	1	1	1	NA
1	500	0.1	3	100	0	5	0	1	1	1	1	1	1	1	1	1	1	1	1	1	1	1
1	100	0.3	3	100	0	5	0	1	1	1	1	1	1	1	1	1	1	1	1	1	1	NA
1	500	0.3	3	100	0	5	0	1	1	1	1	1	1	1	1	1	1	1	1	1	1	1
1	100	0	3	500	0	5	0	0.257	0.082	0.593	0.217	0.128	0.934	0.964	0.992	0.999	0.997	0.674	0.564	0.108	0.224	NA
1	500	0	3	500	0	5	0	0.258	0.065	1	0.578	0.398	1	1	1	1	1	1	1	0.26	0.572	NA
1	100	0.1	3	500	0	5	0	1	1	1	1	1	1	1	1	1	1	1	0.944	1	1	NA
1	500	0.1	3	500	0	5	0	1	1	1	1	1	1	1	1	1	1	1	1	1	1	NA
1	100	0.3	3	500	0	5	0	1	1	1	1	1	1	1	1	1	1	1	1	1	1	NA
1	500	0.3	3	500	0	5	0	1	1	1	1	1	1	1	1	1	1	1	1	1	1	NA
1	100	0	6	10	0	5	0	0.974	0.9	0.996	0.986	0.974	0.995	0.995	0.993	0.982	0.96	0.996	0.976	0.486	0.902	0.999
1	500	0	6	10	0	5	0	0.999	1	1	1	1	1	1	1	1	1	1	1	0.703	0.996	1
1	100	0.1	6	10	0	5	0	1	0.998	1	1	1	1	1	1	1	1	1	1	0.996	1	1
1	500	0.1	6	10	0	5	0	1	1	1	1	1	1	1	1	1	1	1	1	1	1	1
1	100	0.3	6	10	0	5	0	1	1	1	1	1	1	1	1	1	1	1	1	1	1	1
1	500	0.3	6	10	0	5	0	1	1	1	1	1	1	1	1	1	1	1	1	1	1	1
1	100	0	6	50	0	5	0	0.544	0.216	0.86	0.513	0.343	0.934	0.934	0.935	0.913	0.844	0.889	0.862	0.24	0.49	0.83
1	500	0	6	50	0	5	0	0.693	0.252	1	0.997	0.996	1	1	1	1	1	1	1	0.437	0.909	1
1	100	0.1	6	50	0	5	0	0.998	0.975	1	0.999	0.987	1	1	1	1	0.997	1	0.991	0.996	0.985	0.954
1	500	0.1	6	50	0	5	0	1	1	1	1	1	1	1	1	1	1	1	1	1	1	1
1	100	0.3	6	50	0	5	0	1	1	1	1	1	1	1	1	1	1	1	1	1	1	0.999
1	500	0.3	6	50	0	5	0	1	1	1	1	1	1	1	1	1	1	1	1	1	1	1
1	100	0	6	100	0	5	0	0.388	0.154	0.646	0.33	0.205	0.838	0.858	0.864	0.837	0.763	0.715	0.648	0.15	0.313	NA
1	500	0	6	100	0	5	0	0.564	0.175	1	0.932	0.801	1	1	1	1	1	1	1	0.349	0.773	1
1	100	0.1	6	100	0	5	0	0.993	0.976	0.999	0.989	0.982	1	1	1	1	0.992	0.999	0.919	0.993	0.969	NA

1	500	0.1	6	100	0	5	0	1	1	1	1	1	1	1	1	1	1	1	1	1	1	1
1	100	0.3	6	100	0	5	0	1	1	1	1	1	1	1	1	1	1	1	0.999	1	1	NA
1	500	0.3	6	100	0	5	0	1	1	1	1	1	1	1	1	1	1	1	1	1	1	1
1	100	0	6	500	0	5	0	0.191	0.083	0.248	0.168	0.117	0.442	0.503	0.553	0.603	0.595	0.288	0.229	0.079	0.138	NA
1	500	0	6	500	0	5	0	0.247	0.077	0.993	0.332	0.189	1	1	1	1	1	0.999	0.995	0.156	0.364	NA
1	100	0.1	6	500	0	5	0	0.991	0.99	0.991	0.991	0.99	0.994	0.996	0.997	0.999	0.994	0.991	0.498	0.99	0.919	NA
1	500	0.1	6	500	0	5	0	1	1	1	1	1	1	1	1	1	1	1	1	1	1	NA
1	100	0.3	6	500	0	5	0	1	1	1	1	1	1	1	1	1	1	1	0.938	1	1	NA
1	500	0.3	6	500	0	5	0	1	1	1	1	1	1	1	1	1	1	1	1	1	1	NA
1	100	0	1	10	0	10	0	1	1	1	1	1	1	1	1	1	1	1	1	0.632	0.99	1
1	500	0	1	10	0	10	0	1	1	1	1	1	1	1	1	1	1	1	1	0.82	1	1
1	100	0.1	1	10	0	10	0	1	1	1	1	1	1	1	1	1	1	1	1	0.999	1	1
1	500	0.1	1	10	0	10	0	1	1	1	1	1	1	1	1	1	1	1	1	1	1	1
1	100	0.3	1	10	0	10	0	1	1	1	1	1	1	1	1	1	1	1	1	1	1	1
1	500	0.3	1	10	0	10	0	1	1	1	1	1	1	1	1	1	1	1	1	1	1	1
1	100	0	1	50	0	10	0	0.989	0.89	1	0.997	0.964	1	1	1	1	0.999	1	1	0.429	0.849	1
1	500	0	1	50	0	10	0	0.992	1	1	1	1	1	1	1	1	1	1	1	0.618	0.989	1
1	100	0.1	1	50	0	10	0	1	1	1	1	1	1	1	1	1	1	1	1	1	1	1
1	500	0.1	1	50	0	10	0	1	1	1	1	1	1	1	1	1	1	1	1	1	1	1
1	100	0.3	1	50	0	10	0	1	1	1	1	1	1	1	1	1	1	1	1	1	1	1
1	500	0.3	1	50	0	10	0	1	1	1	1	1	1	1	1	1	1	1	1	1	1	1
1	100	0	1	100	0	10	0	0.891	0.449	1	0.908	0.686	1	1	1	1	1	1	1	0.303	0.686	NA
1	500	0	1	100	0	10	0	0.953	0.565	1	1	1	1	1	1	1	1	1	1	0.537	0.96	1
1	100	0.1	1	100	0	10	0	1	1	1	1	1	1	1	1	1	1	1	1	1	1	NA
1	500	0.1	1	100	0	10	0	1	1	1	1	1	1	1	1	1	1	1	1	1	1	1
1	100	0.3	1	100	0	10	0	1	1	1	1	1	1	1	1	1	1	1	1	1	1	NA
1	500	0.3	1	100	0	10	0	1	1	1	1	1	1	1	1	1	1	1	1	1	1	1
1	100	0	1	500	0	10	0	0.503	0.156	0.829	0.366	0.227	0.989	0.996	0.998	0.998	0.993	0.902	0.854	0.148	0.344	NA
1	500	0	1	500	0	10	0	0.59	0.156	1	0.917	0.756	1	1	1	1	1	1	1	0.344	0.737	NA
1	100	0.1	1	500	0	10	0	1	1	1	1	1	1	1	1	1	1	1	1	1	1	NA
1	500	0.1	1	500	0	10	0	1	1	1	1	1	1	1	1	1	1	1	1	1	1	NA
1	100	0.3	1	500	0	10	0	1	1	1	1	1	1	1	1	1	1	1	1	1	1	NA
1	500	0.3	1	500	0	10	0	1	1	1	1	1	1	1	1	1	1	1	1	1	1	NA
1	100	0	3	10	0	10	0	1	1	1	1	1	1	1	1	1	1	1	1	0.604	0.985	1
1	500	0	3	10	0	10	0	1	1	1	1	1	1	1	1	1	1	1	1	0.793	1	1
1	100	0.1	3	10	0	10	0	1	1	1	1	1	1	1	1	1	1	1	1	0.999	1	1
1	500	0.1	3	10	0	10	0	1	1	1	1	1	1	1	1	1	1	1	1	1	1	1
1	100	0.3	3	10	0	10	0	1	1	1	1	1	1	1	1	1	1	1	1	1	1	1
1	500	0.3	3	10	0	10	0	1	1	1	1	1	1	1	1	1	1	1	1	1	1	1
1	100	0	3	50	0	10	0	0.979	0.826	1	0.99	0.912	1	1	1	1	0.999	1	1	0.379	0.792	1

1	500	0	3	50	0	10	0	0.993	1	1	1	1	1	1	1	1	1	1	1	0.586	0.985	1
1	100	0.1	3	50	0	10	0	1	1	1	1	1	1	1	1	1	1	1	1	1	1	1
1	500	0.1	3	50	0	10	0	1	1	1	1	1	1	1	1	1	1	1	1	1	1	1
1	100	0.3	3	50	0	10	0	1	1	1	1	1	1	1	1	1	1	1	1	1	1	1
1	500	0.3	3	50	0	10	0	1	1	1	1	1	1	1	1	1	1	1	1	1	1	1
1	100	0	3	100	0	10	0	0.866	0.433	0.995	0.836	0.594	1	1	1	1	0.993	0.999	0.998	0.268	0.612	NA
1	500	0	3	100	0	10	0	0.958	0.557	1	1	1	1	1	1	1	1	1	1	0.498	0.94	1
1	100	0.1	3	100	0	10	0	1	1	1	1	1	1	1	1	1	1	1	1	1	1	NA
1	500	0.1	3	100	0	10	0	1	1	1	1	1	1	1	1	1	1	1	1	1	1	1
1	100	0.3	3	100	0	10	0	1	1	1	1	1	1	1	1	1	1	1	1	1	1	NA
1	500	0.3	3	100	0	10	0	1	1	1	1	1	1	1	1	1	1	1	1	1	1	1
1	100	0	3	500	0	10	0	0.447	0.144	0.707	0.31	0.192	0.956	0.972	0.98	0.985	0.959	0.796	0.716	0.13	0.288	NA
1	500	0	3	500	0	10	0	0.584	0.153	1	0.88	0.634	1	1	1	1	1	1	1	0.295	0.673	NA
1	100	0.1	3	500	0	10	0	1	1	1	1	1	1	1	1	1	1	1	0.999	1	1	NA
1	500	0.1	3	500	0	10	0	1	1	1	1	1	1	1	1	1	1	1	1	1	1	NA
1	100	0.3	3	500	0	10	0	1	1	1	1	1	1	1	1	1	1	1	1	1	1	NA
1	500	0.3	3	500	0	10	0	1	1	1	1	1	1	1	1	1	1	1	1	1	1	NA
1	100	0	6	10	0	10	0	1	1	1	1	1	1	1	1	0.996	0.987	1	0.994	0.519	0.956	1
1	500	0	6	10	0	10	0	1	1	1	1	1	1	1	1	1	1	1	1	0.736	0.999	1
1	100	0.1	6	10	0	10	0	1	1	1	1	1	1	1	1	1	1	1	1	0.999	1	1
1	500	0.1	6	10	0	10	0	1	1	1	1	1	1	1	1	1	1	1	1	1	1	1
1	100	0.3	6	10	0	10	0	1	1	1	1	1	1	1	1	1	1	1	1	1	1	1
1	500	0.3	6	10	0	10	0	1	1	1	1	1	1	1	1	1	1	1	1	1	1	1
1	100	0	6	50	0	10	0	0.912	0.61	0.99	0.87	0.711	0.996	0.993	0.989	0.972	0.906	0.993	0.987	0.3	0.632	0.996
1	500	0	6	50	0	10	0	0.995	1	1	1	1	1	1	1	1	1	1	1	0.503	0.954	1
1	100	0.1	6	50	0	10	0	1	1	1	1	1	1	1	1	1	1	1	1	1	1	1
1	500	0.1	6	50	0	10	0	1	1	1	1	1	1	1	1	1	1	1	1	1	1	1
1	100	0.3	6	50	0	10	0	1	1	1	1	1	1	1	1	1	1	1	1	1	1	1
1	500	0.3	6	50	0	10	0	1	1	1	1	1	1	1	1	1	1	1	1	1	1	1
1	100	0	6	100	0	10	0	0.719	0.361	0.912	0.638	0.443	0.968	0.968	0.96	0.935	0.836	0.941	0.923	0.203	0.452	NA
1	500	0	6	100	0	10	0	0.962	0.561	1	0.999	0.999	1	1	1	1	1	1	1	0.415	0.87	1
1	100	0.1	6	100	0	10	0	1	1	1	1	1	1	1	1	1	1	1	1	1	1	NA
1	500	0.1	6	100	0	10	0	1	1	1	1	1	1	1	1	1	1	1	1	1	1	1
1	100	0.3	6	100	0	10	0	1	1	1	1	1	1	1	1	1	1	1	1	1	1	NA
1	500	0.3	6	100	0	10	0	1	1	1	1	1	1	1	1	1	1	1	1	1	1	1
1	100	0	6	500	0	10	0	0.316	0.134	0.425	0.232	0.155	0.617	0.659	0.696	0.698	0.596	0.471	0.379	0.108	0.187	NA
1	500	0	6	500	0	10	0	0.569	0.139	1	0.712	0.417	1	1	1	1	1	1	1	0.215	0.494	NA
1	100	0.1	6	500	0	10	0	1	1	1	1	1	1	1	1	1	1	1	0.953	1	1	NA
1	500	0.1	6	500	0	10	0	1	1	1	1	1	1	1	1	1	1	1	1	1	1	NA
1	100	0.3	6	500	0	10	0	1	1	1	1	1	1	1	1	1	1	1	1	1	1	NA

1	500	0.3	6	500	0	10	0	1	1	1	1	1	1	1	1	1	1	1	1	1	1	NA
1	100	0	1	50	0	25	0	1	1	1	1	1	1	1	1	0.999	0.979	1	1	0.419	0.846	1
1	500	0	1	50	0	25	0	1	1	1	1	1	1	1	1	1	1	1	1	0.626	0.989	1
1	100	0.1	1	50	0	25	0	1	1	1	1	1	1	1	1	1	1	1	1	1	1	1
1	500	0.1	1	50	0	25	0	1	1	1	1	1	1	1	1	1	1	1	1	1	1	1
1	100	0.3	1	50	0	25	0	1	1	1	1	1	1	1	1	1	1	1	1	1	1	1
1	500	0.3	1	50	0	25	0	1	1	1	1	1	1	1	1	1	1	1	1	1	1	1
1	100	0	1	100	0	25	0	1	0.975	1	0.997	0.958	1	1	0.999	0.995	0.953	1	0.999	0.31	0.714	NA
1	500	0	1	100	0	25	0	1	1	1	1	1	1	1	1	1	1	1	1	0.552	0.951	1
1	100	0.1	1	100	0	25	0	1	1	1	1	1	1	1	1	1	1	1	1	1	1	NA
1	500	0.1	1	100	0	25	0	1	1	1	1	1	1	1	1	1	1	1	1	1	1	1
1	100	0.3	1	100	0	25	0	1	1	1	1	1	1	1	1	1	1	1	1	1	1	NA
1	500	0.3	1	100	0	25	0	1	1	1	1	1	1	1	1	1	1	1	1	1	1	1
1	100	0	1	500	0	25	0	0.755	0.338	0.863	0.546	0.364	0.957	0.963	0.966	0.937	0.78	0.905	0.869	0.142	0.331	NA
1	500	0	1	500	0	25	0	0.995	0.731	1	1	0.966	1	1	1	1	1	1	1	0.333	0.726	NA
1	100	0.1	1	500	0	25	0	1	1	1	1	1	1	1	1	1	1	1	1	1	1	NA
1	500	0.1	1	500	0	25	0	1	1	1	1	1	1	1	1	1	1	1	1	1	1	NA
1	100	0.3	1	500	0	25	0	1	1	1	1	1	1	1	1	1	1	1	1	1	1	NA
1	500	0.3	1	500	0	25	0	1	1	1	1	1	1	1	1	1	1	1	1	1	1	NA
1	100	0	3	50	0	25	0	1	0.999	1	1	1	1	1	1	0.998	0.959	1	1	0.401	0.833	1
1	500	0	3	50	0	25	0	1	1	1	1	1	1	1	1	1	1	1	1	0.609	0.984	1
1	100	0.1	3	50	0	25	0	1	1	1	1	1	1	1	1	1	1	1	1	1	1	1
1	500	0.1	3	50	0	25	0	1	1	1	1	1	1	1	1	1	1	1	1	1	1	1
1	100	0.3	3	50	0	25	0	1	1	1	1	1	1	1	1	1	1	1	1	1	1	1
1	500	0.3	3	50	0	25	0	1	1	1	1	1	1	1	1	1	1	1	1	1	1	1
1	100	0	3	100	0	25	0	0.998	0.945	1	0.989	0.928	1	1	0.999	0.991	0.917	1	0.999	0.291	0.676	NA
1	500	0	3	100	0	25	0	1	1	1	1	1	1	1	1	1	1	1	1	0.54	0.948	1
1	100	0.1	3	100	0	25	0	1	1	1	1	1	1	1	1	1	1	1	1	1	1	NA
1	500	0.1	3	100	0	25	0	1	1	1	1	1	1	1	1	1	1	1	1	1	1	1
1	100	0.3	3	100	0	25	0	1	1	1	1	1	1	1	1	1	1	1	1	1	1	NA
1	500	0.3	3	100	0	25	0	1	1	1	1	1	1	1	1	1	1	1	1	1	1	1
1	100	0	3	500	0	25	0	0.706	0.32	0.811	0.513	0.344	0.919	0.937	0.926	0.894	0.716	0.866	0.788	0.141	0.318	NA
1	500	0	3	500	0	25	0	0.996	0.728	1	0.998	0.963	1	1	1	1	1	1	1	0.303	0.694	NA
1	100	0.1	3	500	0	25	0	1	1	1	1	1	1	1	1	1	1	1	1	1	1	NA
1	500	0.1	3	500	0	25	0	1	1	1	1	1	1	1	1	1	1	1	1	1	1	NA
1	100	0.3	3	500	0	25	0	1	1	1	1	1	1	1	1	1	1	1	1	1	1	NA
1	500	0.3	3	500	0	25	0	1	1	1	1	1	1	1	1	1	1	1	1	1	1	NA
1	100	0	6	50	0	25	0	0.999	0.993	1	0.999	0.995	1	1	0.997	0.979	0.863	1	1	0.349	0.772	1
1	500	0	6	50	0	25	0	1	1	1	1	1	1	1	1	1	1	1	1	0.557	0.968	1
1	100	0.1	6	50	0	25	0	1	1	1	1	1	1	1	1	1	1	1	1	1	1	1

1	500	0.1	6	50	0	25	0	1	1	1	1	1	1	1	1	1	1	1	1	1	1	1
1	100	0.3	6	50	0	25	0	1	1	1	1	1	1	1	1	1	1	1	1	1	1	1
1	500	0.3	6	50	0	25	0	1	1	1	1	1	1	1	1	1	1	1	1	1	1	1
1	100	0	6	100	0	25	0	0.984	0.868	0.996	0.948	0.851	0.995	0.991	0.983	0.95	0.774	0.996	0.993	0.255	0.574	NA
1	500	0	6	100	0	25	0	1	1	1	1	1	1	1	1	1	1	1	1	0.497	0.918	1
1	100	0.1	6	100	0	25	0	1	1	1	1	1	1	1	1	1	1	1	1	1	1	NA
1	500	0.1	6	100	0	25	0	1	1	1	1	1	1	1	1	1	1	1	1	1	1	1
1	100	0.3	6	100	0	25	0	1	1	1	1	1	1	1	1	1	1	1	1	1	1	NA
1	500	0.3	6	100	0	25	0	1	1	1	1	1	1	1	1	1	1	1	1	1	1	1
1	100	0	6	500	0	25	0	0.574	0.269	0.656	0.425	0.307	0.786	0.786	0.763	0.702	0.495	0.702	0.594	0.131	0.268	NA
1	500	0	6	500	0	25	0	0.991	0.697	1	0.992	0.881	1	1	1	1	1	1	1	0.255	0.626	NA
1	100	0.1	6	500	0	25	0	1	1	1	1	1	1	1	1	1	1	1	1	1	1	NA
1	500	0.1	6	500	0	25	0	1	1	1	1	1	1	1	1	1	1	1	1	1	1	NA
1	100	0.3	6	500	0	25	0	1	1	1	1	1	1	1	1	1	1	1	1	1	1	NA
1	500	0.3	6	500	0	25	0	1	1	1	1	1	1	1	1	1	1	1	1	1	1	NA
1	100	0	1	50	0	50	0	1	1	1	1	1	1	1	1	0.994	0.858	1	1	0.42	0.852	1
1	500	0	1	50	0	50	0	1	1	1	1	1	1	1	1	1	1	1	1	0.622	0.984	1
1	100	0.1	1	50	0	50	0	1	1	1	1	1	1	1	1	1	1	1	1	1	1	1
1	500	0.1	1	50	0	50	0	1	1	1	1	1	1	1	1	1	1	1	1	1	1	1
1	100	0.3	1	50	0	50	0	1	1	1	1	1	1	1	1	1	1	1	1	1	1	1
1	500	0.3	1	50	0	50	0	1	1	1	1	1	1	1	1	1	1	1	1	1	1	1
1	100	0	1	100	0	50	0	1	0.996	1	0.999	0.995	1	0.999	0.996	0.977	0.771	1	1	0.301	0.688	NA
1	500	0	1	100	0	50	0	1	1	1	1	1	1	1	1	1	1	1	1	0.545	0.962	1
1	100	0.1	1	100	0	50	0	1	1	1	1	1	1	1	1	1	1	1	1	1	1	NA
1	500	0.1	1	100	0	50	0	1	1	1	1	1	1	1	1	1	1	1	1	1	1	1
1	100	0.3	1	100	0	50	0	1	1	1	1	1	1	1	1	1	1	1	1	1	1	NA
1	500	0.3	1	100	0	50	0	1	1	1	1	1	1	1	1	1	1	1	1	1	1	1
1	100	0	1	500	0	50	0	0.803	0.476	0.862	0.671	0.511	0.916	0.914	0.879	0.763	0.483	0.893	0.869	0.175	0.33	NA
1	500	0	1	500	0	50	0	1	1	1	1	0.999	1	1	1	1	1	1	1	0.325	0.706	NA
1	100	0.1	1	500	0	50	0	1	1	1	1	1	1	1	1	1	1	1	1	1	1	NA
1	500	0.1	1	500	0	50	0	1	1	1	1	1	1	1	1	1	1	1	1	1	1	NA
1	100	0.3	1	500	0	50	0	1	1	1	1	1	1	1	1	1	1	1	1	1	1	NA
1	500	0.3	1	500	0	50	0	1	1	1	1	1	1	1	1	1	1	1	1	1	1	NA
1	100	0	3	50	0	50	0	1	1	1	1	1	1	1	1	0.99	0.843	1	1	0.406	0.84	1
1	500	0	3	50	0	50	0	1	1	1	1	1	1	1	1	1	1	1	1	0.613	0.982	1
1	100	0.1	3	50	0	50	0	1	1	1	1	1	1	1	1	1	1	1	1	1	1	1
1	500	0.1	3	50	0	50	0	1	1	1	1	1	1	1	1	1	1	1	1	1	1	1
1	100	0.3	3	50	0	50	0	1	1	1	1	1	1	1	1	1	1	1	1	1	1	1
1	500	0.3	3	50	0	50	0	1	1	1	1	1	1	1	1	1	1	1	1	1	1	1
1	100	0	3	100	0	50	0	1	0.993	1	0.999	0.988	1	1	0.996	0.967	0.745	1	1	0.289	0.678	NA

1	500	0	3	100	0	50	0	1	1	1	1	1	1	1	1	1	1	1	1	0.542	0.956	1
1	100	0.1	3	100	0	50	0	1	1	1	1	1	1	1	1	1	1	1	1	1	1	NA
1	500	0.1	3	100	0	50	0	1	1	1	1	1	1	1	1	1	1	1	1	1	1	1
1	100	0.3	3	100	0	50	0	1	1	1	1	1	1	1	1	1	1	1	1	1	1	NA
1	500	0.3	3	100	0	50	0	1	1	1	1	1	1	1	1	1	1	1	1	1	1	1
1	100	0	3	500	0	50	0	0.791	0.475	0.837	0.653	0.477	0.887	0.881	0.846	0.736	0.473	0.855	0.84	0.164	0.332	NA
1	500	0	3	500	0	50	0	1	1	1	1	0.999	1	1	1	1	1	1	1	0.317	0.701	NA
1	100	0.1	3	500	0	50	0	1	1	1	1	1	1	1	1	1	1	1	1	1	1	NA
1	500	0.1	3	500	0	50	0	1	1	1	1	1	1	1	1	1	1	1	1	1	1	NA
1	100	0.3	3	500	0	50	0	1	1	1	1	1	1	1	1	1	1	1	1	1	1	NA
1	500	0.3	3	500	0	50	0	1	1	1	1	1	1	1	1	1	1	1	1	1	1	NA
1	100	0	6	50	0	50	0	1	1	1	1	0.999	1	1	0.995	0.966	0.776	1	1	0.384	0.802	1
1	500	0	6	50	0	50	0	1	1	1	1	1	1	1	1	1	1	1	1	0.59	0.978	1
1	100	0.1	6	50	0	50	0	1	1	1	1	1	1	1	1	1	1	1	1	1	1	1
1	500	0.1	6	50	0	50	0	1	1	1	1	1	1	1	1	1	1	1	1	1	1	1
1	100	0.3	6	50	0	50	0	1	1	1	1	1	1	1	1	1	1	1	1	1	1	1
1	500	0.3	6	50	0	50	0	1	1	1	1	1	1	1	1	1	1	1	1	1	1	1
1	100	0	6	100	0	50	0	1	0.98	1	0.998	0.975	0.999	0.996	0.989	0.931	0.664	1	1	0.252	0.626	NA
1	500	0	6	100	0	50	0	1	1	1	1	1	1	1	1	1	1	1	1	0.514	0.942	1
1	100	0.1	6	100	0	50	0	1	1	1	1	1	1	1	1	1	1	1	1	1	1	NA
1	500	0.1	6	100	0	50	0	1	1	1	1	1	1	1	1	1	1	1	1	1	1	1
1	100	0.3	6	100	0	50	0	1	1	1	1	1	1	1	1	1	1	1	1	1	1	NA
1	500	0.3	6	100	0	50	0	1	1	1	1	1	1	1	1	1	1	1	1	1	1	1
1	100	0	6	500	0	50	0	0.707	0.41	0.754	0.572	0.426	0.801	0.79	0.745	0.636	0.385	0.774	0.726	0.146	0.299	NA
1	500	0	6	500	0	50	0	1	1	1	1	0.996	1	1	1	1	1	1	1	0.286	0.659	NA
1	100	0.1	6	500	0	50	0	1	1	1	1	1	1	1	1	1	1	1	1	1	1	NA
1	500	0.1	6	500	0	50	0	1	1	1	1	1	1	1	1	1	1	1	1	1	1	NA
1	100	0.3	6	500	0	50	0	1	1	1	1	1	1	1	1	1	1	1	1	1	1	NA
1	500	0.3	6	500	0	50	0	1	1	1	1	1	1	1	1	1	1	1	1	1	1	NA
1	100	0	1	10	1	0	0	0.216	0.111	1	0.814	0.953	1	1	1	1	1	1	1	0.6	1	1
1	500	0	1	10	1	0	0	0.206	0.107	1	1	1	1	1	1	1	1	1	1	0.792	1	1
1	100	0.1	1	10	1	0	0	0.496	0.319	1	0.929	0.978	1	1	1	1	1	1	1	0.81	1	1
1	500	0.1	1	10	1	0	0	0.94	0.883	1	1	1	1	1	1	1	1	1	1	1	1	1
1	100	0.3	1	10	1	0	0	0.978	0.96	1	0.999	0.998	1	1	1	1	1	1	1	0.996	1	1
1	500	0.3	1	10	1	0	0	1	1	1	1	1	1	1	1	1	1	1	1	1	1	1
1	100	0	1	50	1	0	0	0.128	0.061	0.993	0.316	0.225	1	1	1	1	1	0.999	0.995	0.294	0.642	0.996
1	500	0	1	50	1	0	0	0.115	0.051	1	0.816	1	1	1	1	1	1	1	1	0.53	0.953	1
1	100	0.1	1	50	1	0	0	0.55	0.45	0.997	0.67	0.611	1	1	1	1	1	0.998	0.998	0.665	0.822	0.992
1	500	0.1	1	50	1	0	0	0.997	0.992	1	1	1	1	1	1	1	1	1	1	1	0.999	1
1	100	0.3	1	50	1	0	0	0.986	0.982	0.999	0.991	0.988	1	1	1	1	1	1	0.999	0.987	0.988	0.995

1	500	0.3	1	50	1	0	0	1	1	1	1	1	1	1	1	1	1	1	1	1	1	1
1	100	0	1	100	1	0	0	0.114	0.078	0.958	0.217	0.15	1	1	1	1	1	0.961	0.914	0.228	0.449	NA
1	500	0	1	100	1	0	0	0.094	0.061	1	0.582	0.841	1	1	1	1	1	1	1	0.421	0.88	1
1	100	0.1	1	100	1	0	0	0.579	0.522	0.946	0.649	0.592	1	1	1	1	1	0.944	0.942	0.63	0.694	NA
1	500	0.1	1	100	1	0	0	0.998	0.996	1	0.998	0.999	1	1	1	1	1	1	1	0.998	1	1
1	100	0.3	1	100	1	0	0	0.983	0.985	0.993	0.988	0.985	0.999	0.999	1	1	1	0.994	0.967	0.985	0.967	NA
1	500	0.3	1	100	1	0	0	1	1	1	1	1	1	1	1	1	1	1	1	1	1	1
1	100	0	1	500	1	0	0	0.086	0.059	0.5	0.111	0.083	0.968	0.997	1	1	1	0.486	0.35	0.126	0.212	NA
1	500	0	1	500	1	0	0	0.086	0.051	1	0.211	0.237	1	1	1	1	1	1	1	0.215	0.457	NA
1	100	0.1	1	500	1	0	0	0.613	0.599	0.706	0.627	0.61	0.886	0.935	0.988	1	1	0.708	0.412	0.628	0.513	NA
1	500	0.1	1	500	1	0	0	1	1	1	1	1	1	1	1	1	1	1	1	1	0.996	NA
1	100	0.3	1	500	1	0	0	0.987	0.987	0.991	0.99	0.99	0.993	0.995	0.996	1	1	0.99	0.618	0.988	0.943	NA
1	500	0.3	1	500	1	0	0	1	1	1	1	1	1	1	1	1	1	1	1	1	1	NA
1	100	0	3	10	1	0	0	0.189	0.121	0.517	0.257	0.196	0.655	0.675	0.685	0.697	0.698	0.57	0.522	0.231	0.438	0.547
1	500	0	3	10	1	0	0	0.202	0.114	1	0.75	0.795	1	1	1	1	1	1	0.999	0.551	0.98	1
1	100	0.1	3	10	1	0	0	0.227	0.147	0.512	0.295	0.219	0.679	0.696	0.707	0.71	0.715	0.582	0.521	0.3	0.475	0.55
1	500	0.1	3	10	1	0	0	0.467	0.312	1	0.837	0.844	1	1	1	1	1	1	0.998	0.841	0.987	1
1	100	0.3	3	10	1	0	0	0.459	0.386	0.608	0.493	0.443	0.678	0.706	0.721	0.725	0.728	0.622	0.53	0.511	0.514	0.536
1	500	0.3	3	10	1	0	0	0.968	0.934	1	0.99	0.981	1	1	1	1	1	1	1	0.988	0.997	1
1	100	0	3	50	1	0	0	0.111	0.059	0.216	0.117	0.09	0.377	0.435	0.463	0.503	0.521	0.239	0.219	0.109	0.159	0.163
1	500	0	3	50	1	0	0	0.115	0.069	0.95	0.26	0.176	1	1	1	1	1	0.991	0.97	0.201	0.495	0.986
1	100	0.1	3	50	1	0	0	0.18	0.151	0.252	0.187	0.154	0.371	0.415	0.476	0.529	0.539	0.266	0.23	0.196	0.21	0.17
1	500	0.1	3	50	1	0	0	0.524	0.429	0.956	0.632	0.55	1	1	1	1	1	0.988	0.961	0.667	0.713	0.984
1	100	0.3	3	50	1	0	0	0.414	0.395	0.44	0.415	0.405	0.472	0.492	0.533	0.58	0.597	0.447	0.239	0.412	0.304	0.162
1	500	0.3	3	50	1	0	0	0.977	0.972	0.994	0.981	0.976	0.998	1	1	1	1	0.995	0.982	0.983	0.96	0.981
1	100	0	3	100	1	0	0	0.094	0.06	0.145	0.091	0.08	0.25	0.293	0.342	0.385	0.407	0.163	0.149	0.083	0.113	NA
1	500	0	3	100	1	0	0	0.11	0.067	0.824	0.192	0.125	0.993	0.996	0.999	0.999	1	0.917	0.885	0.141	0.305	0.883
1	100	0.1	3	100	1	0	0	0.162	0.139	0.197	0.162	0.151	0.264	0.305	0.358	0.406	0.438	0.216	0.142	0.179	0.162	NA
1	500	0.1	3	100	1	0	0	0.606	0.532	0.874	0.636	0.597	0.989	0.994	0.998	1	1	0.92	0.889	0.664	0.592	0.902
1	100	0.3	3	100	1	0	0	0.384	0.376	0.396	0.388	0.374	0.418	0.425	0.446	0.497	0.538	0.387	0.186	0.379	0.278	NA
1	500	0.3	3	100	1	0	0	0.975	0.973	0.985	0.975	0.973	0.992	0.993	0.997	1	1	0.987	0.919	0.979	0.937	0.897
1	100	0	3	500	1	0	0	0.081	0.05	0.092	0.064	0.055	0.138	0.152	0.178	0.226	0.287	0.101	0.088	0.056	0.08	NA
1	500	0	3	500	1	0	0	0.087	0.058	0.327	0.087	0.07	0.813	0.916	0.975	0.996	0.998	0.437	0.342	0.077	0.121	NA
1	100	0.1	3	500	1	0	0	0.18	0.175	0.185	0.182	0.177	0.199	0.206	0.218	0.237	0.317	0.189	0.092	0.185	0.117	NA
1	500	0.1	3	500	1	0	0	0.624	0.604	0.674	0.635	0.626	0.781	0.835	0.918	0.986	0.999	0.695	0.384	0.634	0.449	NA
1	100	0.3	3	500	1	0	0	0.418	0.421	0.419	0.416	0.42	0.423	0.425	0.424	0.438	0.47	0.426	0.113	0.418	0.239	NA
1	500	0.3	3	500	1	0	0	0.975	0.974	0.976	0.975	0.973	0.979	0.98	0.984	0.993	0.999	0.974	0.484	0.971	0.888	NA
1	100	0	6	10	1	0	0	0.109	0.099	0.133	0.112	0.102	0.163	0.164	0.16	0.157	0.153	0.149	0.131	0.09	0.14	0.132
1	500	0	6	10	1	0	0	0.2	0.104	0.643	0.315	0.223	0.783	0.8	0.809	0.811	0.81	0.713	0.633	0.241	0.526	0.71
1	100	0.1	6	10	1	0	0	0.117	0.097	0.149	0.116	0.111	0.179	0.177	0.179	0.171	0.168	0.16	0.135	0.121	0.157	0.131

1	500	0.1	6	10	1	0	0	0.257	0.151	0.649	0.352	0.266	0.793	0.812	0.819	0.824	0.826	0.708	0.646	0.366	0.546	0.697
1	100	0.3	6	10	1	0	0	0.168	0.144	0.186	0.166	0.161	0.196	0.204	0.209	0.192	0.192	0.188	0.138	0.176	0.152	0.129
1	500	0.3	6	10	1	0	0	0.53	0.462	0.713	0.582	0.529	0.794	0.815	0.822	0.826	0.822	0.738	0.652	0.591	0.615	0.702
1	100	0	6	50	1	0	0	0.076	0.056	0.082	0.073	0.064	0.094	0.101	0.101	0.105	0.112	0.088	0.086	0.084	0.072	0.069
1	500	0	6	50	1	0	0	0.105	0.062	0.283	0.111	0.078	0.491	0.55	0.606	0.651	0.678	0.35	0.325	0.098	0.143	0.327
1	100	0.1	6	50	1	0	0	0.106	0.093	0.105	0.099	0.097	0.11	0.113	0.113	0.117	0.115	0.1	0.083	0.099	0.096	0.068
1	500	0.1	6	50	1	0	0	0.223	0.16	0.344	0.224	0.172	0.503	0.561	0.627	0.679	0.69	0.381	0.33	0.235	0.207	0.333
1	100	0.3	6	50	1	0	0	0.161	0.155	0.16	0.157	0.148	0.161	0.169	0.169	0.167	0.157	0.161	0.079	0.152	0.105	0.075
1	500	0.3	6	50	1	0	0	0.518	0.504	0.557	0.528	0.513	0.594	0.613	0.649	0.705	0.739	0.566	0.327	0.527	0.371	0.326
1	100	0	6	100	1	0	0	0.068	0.053	0.071	0.066	0.06	0.079	0.081	0.077	0.075	0.075	0.071	0.07	0.06	0.068	NA
1	500	0	6	100	1	0	0	0.112	0.066	0.218	0.116	0.076	0.401	0.462	0.54	0.599	0.637	0.268	0.248	0.06	0.087	0.223
1	100	0.1	6	100	1	0	0	0.079	0.073	0.083	0.078	0.078	0.091	0.093	0.103	0.098	0.103	0.089	0.064	0.075	0.084	NA
1	500	0.1	6	100	1	0	0	0.23	0.185	0.29	0.237	0.213	0.397	0.448	0.512	0.583	0.628	0.314	0.242	0.232	0.178	0.226
1	100	0.3	6	100	1	0	0	0.136	0.135	0.138	0.135	0.139	0.143	0.145	0.144	0.144	0.128	0.14	0.076	0.143	0.106	NA
1	500	0.3	6	100	1	0	0	0.557	0.549	0.576	0.564	0.556	0.593	0.611	0.63	0.682	0.713	0.587	0.268	0.561	0.328	0.233
1	100	0	6	500	1	0	0	0.059	0.047	0.059	0.051	0.045	0.068	0.073	0.072	0.073	0.067	0.065	0.06	0.051	0.056	NA
1	500	0	6	500	1	0	0	0.079	0.062	0.096	0.062	0.057	0.171	0.21	0.264	0.353	0.449	0.111	0.101	0.056	0.07	NA
1	100	0.1	6	500	1	0	0	0.087	0.089	0.09	0.088	0.083	0.086	0.087	0.081	0.08	0.083	0.091	0.061	0.082	0.059	NA
1	500	0.1	6	500	1	0	0	0.225	0.222	0.237	0.225	0.216	0.252	0.262	0.279	0.331	0.469	0.233	0.105	0.22	0.127	NA
1	100	0.3	6	500	1	0	0	0.153	0.148	0.15	0.15	0.153	0.15	0.152	0.152	0.15	0.141	0.15	0.065	0.146	0.084	NA
1	500	0.3	6	500	1	0	0	0.531	0.521	0.534	0.527	0.525	0.548	0.548	0.555	0.563	0.597	0.532	0.114	0.529	0.313	NA
1	100	0	1	10	2	0	0	0.45	0.199	1	0.989	0.999	1	1	1	1	1	1	0.999	0.588	0.989	1
1	500	0	1	10	2	0	0	0.459	0.203	1	1	1	1	1	1	1	1	1	1	0.783	1	1
1	100	0.1	1	10	2	0	0	0.893	0.739	1	1	1	1	1	1	1	1	1	1	0.969	1	1
1	500	0.1	1	10	2	0	0	1	1	1	1	1	1	1	1	1	1	1	1	1	1	1
1	100	0.3	1	10	2	0	0	1	1	1	1	1	1	1	1	1	1	1	1	1	1	1
1	500	0.3	1	10	2	0	0	1	1	1	1	1	1	1	1	1	1	1	1	1	1	1
1	100	0	1	50	2	0	0	0.22	0.092	0.999	0.524	0.373	1	1	1	1	1	1	1	0.317	0.744	1
1	500	0	1	50	2	0	0	0.214	0.088	1	0.989	1	1	1	1	1	1	1	1	0.561	0.967	1
1	100	0.1	1	50	2	0	0	0.941	0.868	1	0.978	0.956	1	1	1	1	1	1	1	0.975	0.979	1
1	500	0.1	1	50	2	0	0	1	1	1	1	1	1	1	1	1	1	1	1	1	1	1
1	100	0.3	1	50	2	0	0	1	1	1	1	1	1	1	1	1	1	1	1	1	1	1
1	500	0.3	1	50	2	0	0	1	1	1	1	1	1	1	1	1	1	1	1	1	1	1
1	100	0	1	100	2	0	0	0.202	0.091	0.983	0.359	0.225	1	1	1	1	1	0.997	0.992	0.271	0.548	NA
1	500	0	1	100	2	0	0	0.169	0.074	1	0.885	0.985	1	1	1	1	1	1	1	0.472	0.942	1
1	100	0.1	1	100	2	0	0	0.95	0.91	0.999	0.965	0.949	1	1	1	1	1	1	1	0.964	0.958	NA
1	500	0.1	1	100	2	0	0	1	1	1	1	1	1	1	1	1	1	1	1	1	1	1
1	100	0.3	1	100	2	0	0	1	1	1	1	1	1	1	1	1	1	1	1	1	1	NA
1	500	0.3	1	100	2	0	0	1	1	1	1	1	1	1	1	1	1	1	1	1	1	1
1	100	0	1	500	2	0	0	0.13	0.069	0.615	0.167	0.113	0.981	0.996	1	1	1	0.668	0.551	0.137	0.27	NA

1	500	0	1	500	2	0	0	0.124	0.061	1	0.357	0.331	1	1	1	1	1	1	1	0.259	0.587	NA
1	100	0.1	1	500	2	0	0	0.957	0.951	0.978	0.96	0.951	0.997	0.999	1	1	1	0.98	0.752	0.959	0.876	NA
1	500	0.1	1	500	2	0	0	1	1	1	1	1	1	1	1	1	1	1	1	1	1	NA
1	100	0.3	1	500	2	0	0	1	1	1	1	1	1	1	1	1	1	1	0.984	1	1	NA
1	500	0.3	1	500	2	0	0	1	1	1	1	1	1	1	1	1	1	1	1	1	1	NA
1	100	0	3	10	2	0	0	0.43	0.213	0.854	0.578	0.448	0.904	0.904	0.898	0.886	0.864	0.881	0.834	0.345	0.706	0.904
1	500	0	3	10	2	0	0	0.485	0.213	1	0.997	0.999	1	1	1	1	1	1	1	0.612	0.987	1
1	100	0.1	3	10	2	0	0	0.613	0.384	0.934	0.757	0.659	0.956	0.956	0.951	0.946	0.937	0.944	0.888	0.736	0.853	0.921
1	500	0.1	3	10	2	0	0	0.953	0.845	1	1	1	1	1	1	1	1	1	1	1	1	1
1	100	0.3	3	10	2	0	0	0.949	0.909	0.992	0.976	0.952	0.991	0.991	0.991	0.99	0.989	0.993	0.972	0.972	0.959	0.965
1	500	0.3	3	10	2	0	0	1	1	1	1	1	1	1	1	1	1	1	1	1	1	1
1	100	0	3	50	2	0	0	0.202	0.087	0.43	0.206	0.142	0.639	0.691	0.72	0.729	0.697	0.47	0.449	0.137	0.243	0.344
1	500	0	3	50	2	0	0	0.22	0.092	1	0.624	0.439	1	1	1	1	1	1	1	0.321	0.706	1
1	100	0.1	3	50	2	0	0	0.505	0.38	0.67	0.511	0.43	0.803	0.829	0.845	0.85	0.83	0.692	0.537	0.513	0.483	0.371
1	500	0.1	3	50	2	0	0	0.988	0.96	1	0.999	0.995	1	1	1	1	1	1	1	0.999	0.996	1
1	100	0.3	3	50	2	0	0	0.931	0.913	0.943	0.935	0.923	0.966	0.973	0.976	0.98	0.977	0.954	0.702	0.94	0.836	0.466
1	500	0.3	3	50	2	0	0	1	1	1	1	1	1	1	1	1	1	1	1	1	1	1
1	100	0	3	100	2	0	0	0.158	0.076	0.291	0.138	0.094	0.478	0.533	0.573	0.608	0.594	0.34	0.278	0.098	0.186	NA
1	500	0	3	100	2	0	0	0.197	0.08	0.998	0.411	0.277	1	1	1	1	1	1	0.999	0.226	0.527	1
1	100	0.1	3	100	2	0	0	0.492	0.421	0.568	0.48	0.433	0.672	0.7	0.743	0.763	0.742	0.59	0.348	0.481	0.409	NA
1	500	0.1	3	100	2	0	0	0.984	0.974	0.998	0.987	0.985	1	1	1	1	1	1	1	0.989	0.984	1
1	100	0.3	3	100	2	0	0	0.916	0.909	0.933	0.915	0.909	0.942	0.951	0.958	0.96	0.947	0.93	0.526	0.922	0.763	NA
1	500	0.3	3	100	2	0	0	1	1	1	1	1	1	1	1	1	1	1	1	1	1	1
1	100	0	3	500	2	0	0	0.113	0.061	0.134	0.092	0.075	0.216	0.247	0.287	0.367	0.421	0.155	0.124	0.071	0.1	NA
1	500	0	3	500	2	0	0	0.113	0.063	0.683	0.129	0.082	0.989	0.998	0.999	1	1	0.853	0.754	0.098	0.193	NA
1	100	0.1	3	500	2	0	0	0.493	0.476	0.51	0.49	0.483	0.534	0.542	0.568	0.613	0.649	0.514	0.144	0.489	0.307	NA
1	500	0.1	3	500	2	0	0	0.99	0.99	0.995	0.991	0.988	0.998	1	1	1	1	0.995	0.87	0.992	0.958	NA
1	100	0.3	3	500	2	0	0	0.917	0.915	0.918	0.916	0.917	0.921	0.922	0.92	0.925	0.935	0.916	0.261	0.915	0.75	NA
1	500	0.3	3	500	2	0	0	1	1	1	1	1	1	1	1	1	1	1	0.987	1	1	NA
1	100	0	6	10	2	0	0	0.202	0.139	0.272	0.209	0.179	0.286	0.284	0.269	0.264	0.251	0.277	0.255	0.151	0.233	0.269
1	500	0	6	10	2	0	0	0.458	0.217	0.955	0.704	0.582	0.98	0.977	0.978	0.973	0.964	0.967	0.935	0.356	0.798	0.967
1	100	0.1	6	10	2	0	0	0.269	0.189	0.355	0.285	0.237	0.384	0.369	0.34	0.329	0.311	0.357	0.306	0.297	0.305	0.297
1	500	0.1	6	10	2	0	0	0.674	0.444	0.979	0.86	0.791	0.991	0.993	0.992	0.99	0.989	0.988	0.965	0.861	0.916	0.983
1	100	0.3	6	10	2	0	0	0.499	0.433	0.55	0.511	0.472	0.551	0.551	0.532	0.508	0.484	0.548	0.374	0.522	0.395	0.331
1	500	0.3	6	10	2	0	0	0.986	0.964	0.999	0.991	0.985	0.999	0.999	0.999	0.998	0.998	0.999	0.987	0.989	0.983	0.992
1	100	0	6	50	2	0	0	0.113	0.072	0.127	0.103	0.089	0.159	0.162	0.166	0.157	0.146	0.138	0.125	0.086	0.112	0.092
1	500	0	6	50	2	0	0	0.211	0.087	0.617	0.234	0.155	0.847	0.886	0.905	0.916	0.902	0.699	0.64	0.135	0.271	0.679
1	100	0.1	6	50	2	0	0	0.201	0.16	0.211	0.184	0.173	0.228	0.233	0.222	0.214	0.201	0.222	0.126	0.202	0.162	0.108
1	500	0.1	6	50	2	0	0	0.621	0.48	0.831	0.651	0.563	0.938	0.958	0.965	0.964	0.958	0.871	0.738	0.686	0.598	0.759
1	100	0.3	6	50	2	0	0	0.437	0.42	0.438	0.437	0.425	0.451	0.452	0.447	0.449	0.405	0.447	0.181	0.435	0.28	0.113

1	500	0.3	6	50	2	0	0	0.982	0.976	0.99	0.982	0.979	0.996	0.997	0.997	0.996	0.996	0.991	0.875	0.984	0.943	0.841
1	100	0	6	100	2	0	0	0.081	0.062	0.095	0.09	0.08	0.107	0.104	0.103	0.104	0.102	0.096	0.094	0.064	0.081	NA
1	500	0	6	100	2	0	0	0.197	0.085	0.462	0.196	0.124	0.747	0.804	0.85	0.875	0.865	0.552	0.498	0.102	0.165	0.496
1	100	0.1	6	100	2	0	0	0.178	0.154	0.188	0.172	0.156	0.197	0.201	0.208	0.204	0.16	0.191	0.1	0.171	0.15	NA
1	500	0.1	6	100	2	0	0	0.665	0.579	0.772	0.668	0.605	0.887	0.917	0.935	0.946	0.935	0.809	0.582	0.684	0.523	0.553
1	100	0.3	6	100	2	0	0	0.398	0.391	0.404	0.398	0.395	0.41	0.413	0.41	0.408	0.374	0.403	0.132	0.395	0.247	NA
1	500	0.3	6	100	2	0	0	0.982	0.977	0.986	0.982	0.979	0.987	0.988	0.99	0.992	0.992	0.986	0.737	0.985	0.918	0.651
1	100	0	6	500	2	0	0	0.07	0.057	0.082	0.067	0.056	0.095	0.09	0.091	0.087	0.07	0.073	0.076	0.052	0.062	NA
1	500	0	6	500	2	0	0	0.106	0.058	0.167	0.081	0.07	0.335	0.418	0.516	0.642	0.704	0.205	0.162	0.059	0.087	NA
1	100	0.1	6	500	2	0	0	0.185	0.188	0.187	0.181	0.173	0.187	0.187	0.188	0.184	0.153	0.192	0.081	0.174	0.118	NA
1	500	0.1	6	500	2	0	0	0.647	0.635	0.67	0.649	0.641	0.699	0.715	0.755	0.816	0.872	0.673	0.198	0.645	0.444	NA
1	100	0.3	6	500	2	0	0	0.441	0.442	0.438	0.439	0.438	0.437	0.44	0.448	0.443	0.432	0.447	0.104	0.44	0.24	NA
1	500	0.3	6	500	2	0	0	0.976	0.975	0.977	0.976	0.975	0.977	0.978	0.978	0.981	0.984	0.976	0.335	0.975	0.891	NA
1	100	0	1	10	2	2	0	0.941	0.885	1	1	1	1	1	1	1	1	1	1	0.645	0.994	1
1	500	0	1	10	2	2	0	0.964	1	1	1	1	1	1	1	1	1	1	1	0.82	1	1
1	100	0.1	1	10	2	2	0	0.998	1	1	1	1	1	1	1	1	1	1	1	0.996	1	1
1	500	0.1	1	10	2	2	0	1	1	1	1	1	1	1	1	1	1	1	1	1	1	1
1	100	0.3	1	10	2	2	0	1	1	1	1	1	1	1	1	1	1	1	1	1	1	1
1	500	0.3	1	10	2	2	0	1	1	1	1	1	1	1	1	1	1	1	1	1	1	1
1	100	0	1	50	2	2	0	0.501	0.185	1	0.848	0.678	1	1	1	1	1	1	1	0.391	0.847	1
1	500	0	1	50	2	2	0	0.556	0.201	1	1	1	1	1	1	1	1	1	1	0.616	0.98	1
1	100	0.1	1	50	2	2	0	0.999	0.997	1	0.999	0.999	1	1	1	1	1	1	1	1	1	1
1	500	0.1	1	50	2	2	0	1	1	1	1	1	1	1	1	1	1	1	1	1	1	1
1	100	0.3	1	50	2	2	0	1	1	1	1	1	1	1	1	1	1	1	1	1	1	1
1	500	0.3	1	50	2	2	0	1	1	1	1	1	1	1	1	1	1	1	1	1	1	1
1	100	0	1	100	2	2	0	0.358	0.133	1	0.61	0.384	1	1	1	1	1	1	1	0.309	0.672	NA
1	500	0	1	100	2	2	0	0.402	0.131	1	0.995	1	1	1	1	1	1	1	1	0.534	0.948	1
1	100	0.1	1	100	2	2	0	1	1	1	1	1	1	1	1	1	1	1	1	1	0.999	NA
1	500	0.1	1	100	2	2	0	1	1	1	1	1	1	1	1	1	1	1	1	1	1	1
1	100	0.3	1	100	2	2	0	1	1	1	1	1	1	1	1	1	1	1	1	1	1	NA
1	500	0.3	1	100	2	2	0	1	1	1	1	1	1	1	1	1	1	1	1	1	1	1
1	100	0	1	500	2	2	0	0.188	0.082	0.826	0.222	0.151	1	1	1	1	1	0.878	0.815	0.156	0.316	NA
1	500	0	1	500	2	2	0	0.202	0.064	1	0.61	0.527	1	1	1	1	1	1	1	0.31	0.675	NA
1	100	0.1	1	500	2	2	0	1	1	1	1	1	1	1	1	1	1	1	0.989	1	0.998	NA
1	500	0.1	1	500	2	2	0	1	1	1	1	1	1	1	1	1	1	1	1	1	1	NA
1	100	0.3	1	500	2	2	0	1	1	1	1	1	1	1	1	1	1	1	1	1	1	NA
1	500	0.3	1	500	2	2	0	1	1	1	1	1	1	1	1	1	1	1	1	1	1	NA
1	100	0	3	10	2	2	0	0.89	0.696	1	0.996	0.992	1	1	1	1	1	1	0.997	0.55	0.963	1
1	500	0	3	10	2	2	0	0.95	0.999	1	1	1	1	1	1	1	1	1	1	0.771	1	1
1	100	0.1	3	10	2	2	0	0.996	0.987	1	1	1	1	1	1	1	1	1	1	0.991	0.999	1

1	500	0.1	3	10	2	2	0	1	1	1	1	1	1	1	1	1	1	1	1	1	1	1
1	100	0.3	3	10	2	2	0	1	1	1	1	1	1	1	1	1	1	1	1	1	1	1
1	500	0.3	3	10	2	2	0	1	1	1	1	1	1	1	1	1	1	1	1	1	1	1
1	100	0	3	50	2	2	0	0.432	0.177	0.988	0.579	0.383	1	1	1	1	1	0.995	0.986	0.275	0.647	0.995
1	500	0	3	50	2	2	0	0.548	0.194	1	0.999	1	1	1	1	1	1	1	1	0.517	0.944	1
1	100	0.1	3	50	2	2	0	0.992	0.973	1	0.995	0.989	1	1	1	1	1	1	1	0.995	0.99	1
1	500	0.1	3	50	2	2	0	1	1	1	1	1	1	1	1	1	1	1	1	1	1	1
1	100	0.3	3	50	2	2	0	1	1	1	1	1	1	1	1	1	1	1	1	1	1	1
1	500	0.3	3	50	2	2	0	1	1	1	1	1	1	1	1	1	1	1	1	1	1	1
1	100	0	3	100	2	2	0	0.324	0.128	0.901	0.376	0.228	0.999	0.999	1	1	1	0.938	0.929	0.217	0.457	NA
1	500	0	3	100	2	2	0	0.414	0.108	1	0.933	0.914	1	1	1	1	1	1	1	0.432	0.868	1
1	100	0.1	3	100	2	2	0	0.993	0.981	1	0.994	0.99	1	1	1	1	1	1	0.993	0.99	0.984	NA
1	500	0.1	3	100	2	2	0	1	1	1	1	1	1	1	1	1	1	1	1	1	1	1
1	100	0.3	3	100	2	2	0	1	1	1	1	1	1	1	1	1	1	1	1	1	1	NA
1	500	0.3	3	100	2	2	0	1	1	1	1	1	1	1	1	1	1	1	1	1	1	1
1	100	0	3	500	2	2	0	0.17	0.085	0.454	0.164	0.109	0.829	0.921	0.974	0.991	0.995	0.516	0.381	0.106	0.192	NA
1	500	0	3	500	2	2	0	0.196	0.072	1	0.387	0.258	1	1	1	1	1	1	1	0.216	0.471	NA
1	100	0.1	3	500	2	2	0	0.997	0.991	0.999	0.996	0.992	0.999	1	1	1	1	0.999	0.671	0.996	0.951	NA
1	500	0.1	3	500	2	2	0	1	1	1	1	1	1	1	1	1	1	1	1	1	1	NA
1	100	0.3	3	500	2	2	0	1	1	1	1	1	1	1	1	1	1	1	0.987	1	1	NA
1	500	0.3	3	500	2	2	0	1	1	1	1	1	1	1	1	1	1	1	1	1	1	NA
1	100	0	6	10	2	2	0	0.696	0.447	0.943	0.775	0.678	0.944	0.939	0.93	0.911	0.889	0.953	0.91	0.368	0.778	0.96
1	500	0	6	10	2	2	0	0.943	0.906	1	1	1	1	1	1	1	1	1	1	0.63	0.996	1
1	100	0.1	6	10	2	2	0	0.927	0.812	0.994	0.962	0.942	0.994	0.993	0.992	0.986	0.979	0.994	0.982	0.944	0.969	0.99
1	500	0.1	6	10	2	2	0	1	1	1	1	1	1	1	1	1	1	1	1	1	1	1
1	100	0.3	6	10	2	2	0	1	0.998	1	1	1	1	1	1	1	1	1	0.998	1	0.999	0.998
1	500	0.3	6	10	2	2	0	1	1	1	1	1	1	1	1	1	1	1	1	1	1	1
1	100	0	6	50	2	2	0	0.292	0.122	0.543	0.29	0.187	0.725	0.754	0.76	0.745	0.7	0.612	0.547	0.165	0.306	0.435
1	500	0	6	50	2	2	0	0.509	0.187	1	0.862	0.691	1	1	1	1	1	1	1	0.344	0.78	1
1	100	0.1	6	50	2	2	0	0.831	0.717	0.932	0.824	0.758	0.968	0.967	0.963	0.956	0.916	0.944	0.736	0.862	0.769	0.581
1	500	0.1	6	50	2	2	0	1	0.999	1	1	1	1	1	1	1	1	1	1	1	1	1
1	100	0.3	6	50	2	2	0	1	1	1	1	1	1	1	1	1	0.998	1	0.955	1	0.989	0.781
1	500	0.3	6	50	2	2	0	1	1	1	1	1	1	1	1	1	1	1	1	1	1	1
1	100	0	6	100	2	2	0	0.228	0.108	0.385	0.192	0.13	0.564	0.598	0.628	0.639	0.607	0.426	0.364	0.121	0.208	NA
1	500	0	6	100	2	2	0	0.412	0.142	1	0.657	0.437	1	1	1	1	1	1	0.999	0.26	0.597	1
1	100	0.1	6	100	2	2	0	0.803	0.729	0.878	0.792	0.732	0.914	0.922	0.931	0.923	0.873	0.883	0.521	0.8	0.662	NA
1	500	0.1	6	100	2	2	0	1	1	1	1	1	1	1	1	1	1	1	1	1	1	1
1	100	0.3	6	100	2	2	0	0.999	0.999	0.999	0.999	0.999	0.999	0.999	0.999	0.999	0.998	0.999	0.847	0.999	0.985	NA
1	500	0.3	6	100	2	2	0	1	1	1	1	1	1	1	1	1	1	1	1	1	1	1
1	100	0	6	500	2	2	0	0.123	0.066	0.16	0.105	0.092	0.263	0.297	0.325	0.389	0.417	0.179	0.149	0.072	0.102	NA

1	500	0	6	500	2	2	0	0.18	0.071	0.817	0.208	0.126	0.997	1	1	1	1	0.926	0.857	0.12	0.222	NA
1	100	0.1	6	500	2	2	0	0.809	0.79	0.812	0.805	0.797	0.827	0.833	0.845	0.858	0.841	0.815	0.211	0.794	0.608	NA
1	500	0.1	6	500	2	2	0	1	1	1	1	1	1	1	1	1	1	1	0.985	1	1	NA
1	100	0.3	6	500	2	2	0	0.999	0.999	0.999	0.999	0.999	0.999	0.999	0.999	0.999	1	0.999	0.537	0.999	0.978	NA
1	500	0.3	6	500	2	2	0	1	1	1	1	1	1	1	1	1	1	1	1	1	1	NA
1	100	0	1	10	5	0	0	0.999	1	1	1	1	1	1	1	1	1	1	0.999	0.642	0.984	1
1	500	0	1	10	5	0	0	0.999	1	1	1	1	1	1	1	1	1	1	1	0.792	1	1
1	100	0.1	1	10	5	0	0	1	1	1	1	1	1	1	1	1	1	1	1	0.999	1	1
1	500	0.1	1	10	5	0	0	1	1	1	1	1	1	1	1	1	1	1	1	1	1	1
1	100	0.3	1	10	5	0	0	1	1	1	1	1	1	1	1	1	1	1	1	1	1	1
1	500	0.3	1	10	5	0	0	1	1	1	1	1	1	1	1	1	1	1	1	1	1	1
1	100	0	1	50	5	0	0	0.676	0.263	1	0.894	0.712	1	1	1	1	1	1	1	0.405	0.803	1
1	500	0	1	50	5	0	0	0.711	0.262	1	1	1	1	1	1	1	1	1	1	0.62	0.986	1
1	100	0.1	1	50	5	0	0	1	1	1	1	1	1	1	1	1	1	1	1	1	1	1
1	500	0.1	1	50	5	0	0	1	1	1	1	1	1	1	1	1	1	1	1	1	1	1
1	100	0.3	1	50	5	0	0	1	1	1	1	1	1	1	1	1	1	1	1	1	1	1
1	500	0.3	1	50	5	0	0	1	1	1	1	1	1	1	1	1	1	1	1	1	1	1
1	100	0	1	100	5	0	0	0.508	0.18	0.997	0.643	0.392	1	1	1	1	1	0.999	0.998	0.282	0.605	NA
1	500	0	1	100	5	0	0	0.54	0.165	1	1	1	1	1	1	1	1	1	1	0.523	0.948	1
1	100	0.1	1	100	5	0	0	1	1	1	1	1	1	1	1	1	1	1	1	1	1	NA
1	500	0.1	1	100	5	0	0	1	1	1	1	1	1	1	1	1	1	1	1	1	1	1
1	100	0.3	1	100	5	0	0	1	1	1	1	1	1	1	1	1	1	1	1	1	1	NA
1	500	0.3	1	100	5	0	0	1	1	1	1	1	1	1	1	1	1	1	1	1	1	1
1	100	0	1	500	5	0	0	0.266	0.095	0.718	0.246	0.147	0.985	0.999	0.999	1	1	0.795	0.74	0.131	0.275	NA
1	500	0	1	500	5	0	0	0.248	0.087	1	0.663	0.51	1	1	1	1	1	1	1	0.3	0.653	NA
1	100	0.1	1	500	5	0	0	1	1	1	1	1	1	1	1	1	1	1	0.989	1	1	NA
1	500	0.1	1	500	5	0	0	1	1	1	1	1	1	1	1	1	1	1	1	1	1	NA
1	100	0.3	1	500	5	0	0	1	1	1	1	1	1	1	1	1	1	1	1	1	1	NA
1	500	0.3	1	500	5	0	0	1	1	1	1	1	1	1	1	1	1	1	1	1	1	NA
1	100	0	3	10	5	0	0	0.974	0.9	0.996	0.986	0.974	0.995	0.995	0.993	0.982	0.96	0.996	0.976	0.486	0.902	0.999
1	500	0	3	10	5	0	0	0.999	1	1	1	1	1	1	1	1	1	1	1	0.703	0.996	1
1	100	0.1	3	10	5	0	0	1	0.998	1	1	1	1	1	1	1	1	1	1	0.996	1	1
1	500	0.1	3	10	5	0	0	1	1	1	1	1	1	1	1	1	1	1	1	1	1	1
1	100	0.3	3	10	5	0	0	1	1	1	1	1	1	1	1	1	1	1	1	1	1	1
1	500	0.3	3	10	5	0	0	1	1	1	1	1	1	1	1	1	1	1	1	1	1	1
1	100	0	3	50	5	0	0	0.544	0.216	0.86	0.513	0.343	0.934	0.934	0.935	0.913	0.844	0.889	0.862	0.24	0.49	0.83
1	500	0	3	50	5	0	0	0.693	0.252	1	0.997	0.996	1	1	1	1	1	1	1	0.437	0.909	1
1	100	0.1	3	50	5	0	0	0.998	0.975	1	0.999	0.987	1	1	1	1	0.997	1	0.991	0.996	0.985	0.954
1	500	0.1	3	50	5	0	0	1	1	1	1	1	1	1	1	1	1	1	1	1	1	1
1	100	0.3	3	50	5	0	0	1	1	1	1	1	1	1	1	1	1	1	1	1	1	0.999

1	500	0.3	3	50	5	0	0	1	1	1	1	1	1	1	1	1	1	1	1	1	1	1
1	100	0	3	100	5	0	0	0.388	0.154	0.646	0.33	0.205	0.838	0.858	0.864	0.837	0.763	0.715	0.648	0.15	0.313	NA
1	500	0	3	100	5	0	0	0.564	0.175	1	0.932	0.801	1	1	1	1	1	1	1	0.349	0.773	1
1	100	0.1	3	100	5	0	0	0.993	0.976	0.999	0.989	0.982	1	1	1	1	0.992	0.999	0.919	0.993	0.969	NA
1	500	0.1	3	100	5	0	0	1	1	1	1	1	1	1	1	1	1	1	1	1	1	1
1	100	0.3	3	100	5	0	0	1	1	1	1	1	1	1	1	1	1	1	0.999	1	1	NA
1	500	0.3	3	100	5	0	0	1	1	1	1	1	1	1	1	1	1	1	1	1	1	1
1	100	0	3	500	5	0	0	0.191	0.083	0.248	0.168	0.117	0.442	0.503	0.553	0.603	0.595	0.288	0.229	0.079	0.138	NA
1	500	0	3	500	5	0	0	0.247	0.077	0.993	0.332	0.189	1	1	1	1	1	0.999	0.995	0.156	0.364	NA
1	100	0.1	3	500	5	0	0	0.991	0.99	0.991	0.991	0.99	0.994	0.996	0.997	0.999	0.994	0.991	0.498	0.99	0.919	NA
1	500	0.1	3	500	5	0	0	1	1	1	1	1	1	1	1	1	1	1	1	1	1	NA
1	100	0.3	3	500	5	0	0	1	1	1	1	1	1	1	1	1	1	1	0.938	1	1	NA
1	500	0.3	3	500	5	0	0	1	1	1	1	1	1	1	1	1	1	1	1	1	1	NA
1	100	0	6	10	5	0	0	0.553	0.39	0.661	0.549	0.475	0.619	0.596	0.548	0.489	0.452	0.654	0.611	0.263	0.516	0.65
1	500	0	6	10	5	0	0	0.997	0.991	1	1	1	1	1	1	1	1	1	0.997	0.515	0.96	1
1	100	0.1	6	10	5	0	0	0.903	0.784	0.933	0.902	0.865	0.914	0.9	0.878	0.827	0.763	0.931	0.851	0.902	0.866	0.85
1	500	0.1	6	10	5	0	0	1	1	1	1	1	1	1	1	1	1	1	1	1	1	1
1	100	0.3	6	10	5	0	0	0.997	0.996	0.998	0.997	0.998	0.997	0.997	0.997	0.995	0.99	0.998	0.982	1	0.989	0.976
1	500	0.3	6	10	5	0	0	1	1	1	1	1	1	1	1	1	1	1	1	1	1	1
1	100	0	6	50	5	0	0	0.235	0.122	0.286	0.198	0.149	0.346	0.345	0.323	0.301	0.25	0.308	0.279	0.131	0.19	0.198
1	500	0	6	50	5	0	0	0.664	0.25	0.995	0.749	0.526	0.999	1	1	0.999	0.996	0.998	0.989	0.231	0.583	0.998
1	100	0.1	6	50	5	0	0	0.723	0.633	0.776	0.705	0.644	0.802	0.792	0.774	0.72	0.6	0.789	0.475	0.757	0.588	0.289
1	500	0.1	6	50	5	0	0	1	0.999	1	1	1	1	1	1	1	1	1	1	1	1	1
1	100	0.3	6	50	5	0	0	0.992	0.987	0.994	0.992	0.991	0.995	0.995	0.993	0.993	0.987	0.993	0.808	0.992	0.966	0.496
1	500	0.3	6	50	5	0	0	1	1	1	1	1	1	1	1	1	1	1	1	1	1	1
1	100	0	6	100	5	0	0	0.165	0.096	0.201	0.129	0.108	0.244	0.235	0.225	0.207	0.177	0.218	0.182	0.088	0.13	NA
1	500	0	6	100	5	0	0	0.507	0.194	0.933	0.545	0.332	0.992	0.993	0.995	0.995	0.988	0.961	0.946	0.18	0.368	0.956
1	100	0.1	6	100	5	0	0	0.706	0.634	0.723	0.676	0.621	0.732	0.724	0.712	0.673	0.55	0.73	0.309	0.688	0.523	NA
1	500	0.1	6	100	5	0	0	0.999	0.998	0.999	0.999	0.998	1	1	1	1	1	1	0.997	0.999	0.997	0.997
1	100	0.3	6	100	5	0	0	0.99	0.987	0.991	0.991	0.99	0.991	0.991	0.991	0.991	0.985	0.992	0.612	0.991	0.926	NA
1	500	0.3	6	100	5	0	0	1	1	1	1	1	1	1	1	1	1	1	1	1	1	1
1	100	0	6	500	5	0	0	0.103	0.068	0.108	0.081	0.071	0.115	0.121	0.109	0.11	0.092	0.111	0.092	0.066	0.083	NA
1	500	0	6	500	5	0	0	0.214	0.088	0.443	0.168	0.103	0.797	0.862	0.924	0.952	0.957	0.566	0.458	0.081	0.138	NA
1	100	0.1	6	500	5	0	0	0.709	0.697	0.713	0.705	0.69	0.717	0.717	0.711	0.693	0.592	0.714	0.142	0.698	0.453	NA
1	500	0.1	6	500	5	0	0	1	0.999	1	1	1	1	1	1	1	1	1	0.799	1	0.994	NA
1	100	0.3	6	500	5	0	0	0.992	0.991	0.992	0.99	0.99	0.992	0.992	0.991	0.991	0.984	0.992	0.353	0.989	0.921	NA
1	500	0.3	6	500	5	0	0	1	1	1	1	1	1	1	1	1	1	1	0.998	1	1	NA
1	100	0	1	10	5	5	0	1	1	1	1	1	1	1	1	1	1	1	1	0.621	0.991	1
1	500	0	1	10	5	5	0	1	1	1	1	1	1	1	1	1	1	1	1	0.824	1	1
1	100	0.1	1	10	5	5	0	1	1	1	1	1	1	1	1	1	1	1	1	0.999	1	1

1	500	0.1	1	10	5	5	0	1	1	1	1	1	1	1	1	1	1	1	1	1	1	1
1	100	0.3	1	10	5	5	0	1	1	1	1	1	1	1	1	1	1	1	1	1	1	1
1	500	0.3	1	10	5	5	0	1	1	1	1	1	1	1	1	1	1	1	1	1	1	1
1	100	0	1	50	5	5	0	0.963	0.696	1	0.987	0.945	1	1	1	1	1	1	1	0.413	0.843	1
1	500	0	1	50	5	5	0	0.993	1	1	1	1	1	1	1	1	1	1	1	0.617	0.989	1
1	100	0.1	1	50	5	5	0	1	1	1	1	1	1	1	1	1	1	1	1	1	1	1
1	500	0.1	1	50	5	5	0	1	1	1	1	1	1	1	1	1	1	1	1	1	1	1
1	100	0.3	1	50	5	5	0	1	1	1	1	1	1	1	1	1	1	1	1	1	1	1
1	500	0.3	1	50	5	5	0	1	1	1	1	1	1	1	1	1	1	1	1	1	1	1
1	100	0	1	100	5	5	0	0.814	0.354	1	0.856	0.631	1	1	1	1	1	1	1	0.312	0.699	NA
1	500	0	1	100	5	5	0	0.951	0.543	1	1	1	1	1	1	1	1	1	1	0.535	0.955	1
1	100	0.1	1	100	5	5	0	1	1	1	1	1	1	1	1	1	1	1	1	1	1	NA
1	500	0.1	1	100	5	5	0	1	1	1	1	1	1	1	1	1	1	1	1	1	1	1
1	100	0.3	1	100	5	5	0	1	1	1	1	1	1	1	1	1	1	1	1	1	1	NA
1	500	0.3	1	100	5	5	0	1	1	1	1	1	1	1	1	1	1	1	1	1	1	1
1	100	0	1	500	5	5	0	0.412	0.131	0.815	0.328	0.182	0.992	0.999	0.999	1	1	0.896	0.845	0.151	0.343	NA
1	500	0	1	500	5	5	0	0.585	0.158	1	0.898	0.716	1	1	1	1	1	1	1	0.318	0.723	NA
1	100	0.1	1	500	5	5	0	1	1	1	1	1	1	1	1	1	1	1	1	1	1	NA
1	500	0.1	1	500	5	5	0	1	1	1	1	1	1	1	1	1	1	1	1	1	1	NA
1	100	0.3	1	500	5	5	0	1	1	1	1	1	1	1	1	1	1	1	1	1	1	NA
1	500	0.3	1	500	5	5	0	1	1	1	1	1	1	1	1	1	1	1	1	1	1	NA
1	100	0	3	10	5	5	0	1	1	1	1	1	1	1	1	1	1	1	1	0.58	0.981	1
1	500	0	3	10	5	5	0	1	1	1	1	1	1	1	1	1	1	1	1	0.793	0.999	1
1	100	0.1	3	10	5	5	0	1	1	1	1	1	1	1	1	1	1	1	1	0.999	1	1
1	500	0.1	3	10	5	5	0	1	1	1	1	1	1	1	1	1	1	1	1	1	1	1
1	100	0.3	3	10	5	5	0	1	1	1	1	1	1	1	1	1	1	1	1	1	1	1
1	500	0.3	3	10	5	5	0	1	1	1	1	1	1	1	1	1	1	1	1	1	1	1
1	100	0	3	50	5	5	0	0.929	0.628	1	0.963	0.825	1	1	1	1	1	1	1	0.357	0.768	1
1	500	0	3	50	5	5	0	0.994	0.991	1	1	1	1	1	1	1	1	1	1	0.581	0.979	1
1	100	0.1	3	50	5	5	0	1	1	1	1	1	1	1	1	1	1	1	1	1	1	1
1	500	0.1	3	50	5	5	0	1	1	1	1	1	1	1	1	1	1	1	1	1	1	1
1	100	0.3	3	50	5	5	0	1	1	1	1	1	1	1	1	1	1	1	1	1	1	1
1	500	0.3	3	50	5	5	0	1	1	1	1	1	1	1	1	1	1	1	1	1	1	1
1	100	0	3	100	5	5	0	0.744	0.341	0.992	0.73	0.477	1	1	1	1	0.996	0.998	0.994	0.268	0.588	NA
1	500	0	3	100	5	5	0	0.951	0.542	1	1	1	1	1	1	1	1	1	1	0.48	0.921	1
1	100	0.1	3	100	5	5	0	1	1	1	1	1	1	1	1	1	1	1	1	1	1	NA
1	500	0.1	3	100	5	5	0	1	1	1	1	1	1	1	1	1	1	1	1	1	1	1
1	100	0.3	3	100	5	5	0	1	1	1	1	1	1	1	1	1	1	1	1	1	1	NA
1	500	0.3	3	100	5	5	0	1	1	1	1	1	1	1	1	1	1	1	1	1	1	1
1	100	0	3	500	5	5	0	0.372	0.123	0.635	0.278	0.173	0.918	0.947	0.979	0.985	0.97	0.719	0.619	0.133	0.269	NA

1	500	0	3	500	5	5	0	0.583	0.154	1	0.802	0.565	1	1	1	1	1	1	1	0.28	0.634	NA
1	100	0.1	3	500	5	5	0	1	1	1	1	1	1	1	1	1	1	1	0.994	1	1	NA
1	500	0.1	3	500	5	5	0	1	1	1	1	1	1	1	1	1	1	1	1	1	1	NA
1	100	0.3	3	500	5	5	0	1	1	1	1	1	1	1	1	1	1	1	1	1	1	NA
1	500	0.3	3	500	5	5	0	1	1	1	1	1	1	1	1	1	1	1	1	1	1	NA
1	100	0	6	10	5	5	0	0.999	0.985	1	0.998	0.998	1	0.999	0.999	0.99	0.962	1	0.989	0.466	0.918	1
1	500	0	6	10	5	5	0	1	1	1	1	1	1	1	1	1	1	1	1	0.713	0.998	1
1	100	0.1	6	10	5	5	0	1	1	1	1	1	1	1	1	1	0.999	1	1	0.999	1	1
1	500	0.1	6	10	5	5	0	1	1	1	1	1	1	1	1	1	1	1	1	1	1	1
1	100	0.3	6	10	5	5	0	1	1	1	1	1	1	1	1	1	1	1	1	1	1	1
1	500	0.3	6	10	5	5	0	1	1	1	1	1	1	1	1	1	1	1	1	1	1	1
1	100	0	6	50	5	5	0	0.759	0.422	0.933	0.71	0.538	0.965	0.965	0.952	0.916	0.831	0.948	0.933	0.262	0.546	0.916
1	500	0	6	50	5	5	0	0.986	0.911	1	1	1	1	1	1	1	1	1	1	0.472	0.926	1
1	100	0.1	6	50	5	5	0	1	1	1	1	1	1	1	1	1	1	1	1	1	1	1
1	500	0.1	6	50	5	5	0	1	1	1	1	1	1	1	1	1	1	1	1	1	1	1
1	100	0.3	6	50	5	5	0	1	1	1	1	1	1	1	1	1	1	1	1	1	1	1
1	500	0.3	6	50	5	5	0	1	1	1	1	1	1	1	1	1	1	1	1	1	1	1
1	100	0	6	100	5	5	0	0.55	0.256	0.776	0.475	0.304	0.903	0.9	0.9	0.877	0.785	0.83	0.762	0.178	0.369	NA
1	500	0	6	100	5	5	0	0.928	0.488	1	0.997	0.966	1	1	1	1	1	1	1	0.365	0.797	1
1	100	0.1	6	100	5	5	0	1	1	1	1	1	1	1	1	1	1	1	0.997	1	1	NA
1	500	0.1	6	100	5	5	0	1	1	1	1	1	1	1	1	1	1	1	1	1	1	1
1	100	0.3	6	100	5	5	0	1	1	1	1	1	1	1	1	1	1	1	1	1	1	NA
1	500	0.3	6	100	5	5	0	1	1	1	1	1	1	1	1	1	1	1	1	1	1	1
1	100	0	6	500	5	5	0	0.249	0.103	0.314	0.176	0.129	0.452	0.487	0.537	0.573	0.532	0.346	0.25	0.099	0.153	NA
1	500	0	6	500	5	5	0	0.517	0.13	0.996	0.564	0.304	1	1	1	1	1	0.999	0.999	0.176	0.42	NA
1	100	0.1	6	500	5	5	0	1	1	1	1	1	1	1	1	1	1	1	0.815	1	1	NA
1	500	0.1	6	500	5	5	0	1	1	1	1	1	1	1	1	1	1	1	1	1	1	NA
1	100	0.3	6	500	5	5	0	1	1	1	1	1	1	1	1	1	1	1	1	1	1	NA
1	500	0.3	6	500	5	5	0	1	1	1	1	1	1	1	1	1	1	1	1	1	1	NA
1	100	0	1	10	10	0	0	1	1	1	1	1	1	1	1	1	1	1	1	0.618	0.988	1
1	500	0	1	10	10	0	0	1	1	1	1	1	1	1	1	1	1	1	1	0.807	1	1
1	100	0.1	1	10	10	0	0	1	1	1	1	1	1	1	1	1	1	1	1	0.999	1	1
1	500	0.1	1	10	10	0	0	1	1	1	1	1	1	1	1	1	1	1	1	1	1	1
1	100	0.3	1	10	10	0	0	1	1	1	1	1	1	1	1	1	1	1	1	1	1	1
1	500	0.3	1	10	10	0	0	1	1	1	1	1	1	1	1	1	1	1	1	1	1	1
1	100	0	1	50	10	0	0	0.988	0.872	1	0.993	0.941	1	1	1	1	0.999	1	1	0.403	0.829	1
1	500	0	1	50	10	0	0	0.993	1	1	1	1	1	1	1	1	1	1	1	0.604	0.986	1
1	100	0.1	1	50	10	0	0	1	1	1	1	1	1	1	1	1	1	1	1	1	1	1
1	500	0.1	1	50	10	0	0	1	1	1	1	1	1	1	1	1	1	1	1	1	1	1
1	100	0.3	1	50	10	0	0	1	1	1	1	1	1	1	1	1	1	1	1	1	1	1

1	500	0.3	1	50	10	0	0	1	1	1	1	1	1	1	1	1	1	1	1	1	1	1
1	100	0	1	100	10	0	0	0.881	0.45	0.999	0.878	0.649	1	1	1	1	1	1	1	0.281	0.66	NA
1	500	0	1	100	10	0	0	0.957	0.557	1	1	1	1	1	1	1	1	1	1	0.524	0.951	1
1	100	0.1	1	100	10	0	0	1	1	1	1	1	1	1	1	1	1	1	1	1	1	NA
1	500	0.1	1	100	10	0	0	1	1	1	1	1	1	1	1	1	1	1	1	1	1	1
1	100	0.3	1	100	10	0	0	1	1	1	1	1	1	1	1	1	1	1	1	1	1	NA
1	500	0.3	1	100	10	0	0	1	1	1	1	1	1	1	1	1	1	1	1	1	1	1
1	100	0	1	500	10	0	0	0.481	0.156	0.778	0.355	0.224	0.981	0.989	0.994	0.996	0.983	0.872	0.789	0.135	0.32	NA
1	500	0	1	500	10	0	0	0.596	0.146	1	0.907	0.699	1	1	1	1	1	1	1	0.318	0.71	NA
1	100	0.1	1	500	10	0	0	1	1	1	1	1	1	1	1	1	1	1	1	1	1	NA
1	500	0.1	1	500	10	0	0	1	1	1	1	1	1	1	1	1	1	1	1	1	1	NA
1	100	0.3	1	500	10	0	0	1	1	1	1	1	1	1	1	1	1	1	1	1	1	NA
1	500	0.3	1	500	10	0	0	1	1	1	1	1	1	1	1	1	1	1	1	1	1	NA
1	100	0	3	10	10	0	0	1	1	1	1	1	1	1	1	0.996	0.987	1	0.994	0.519	0.956	1
1	500	0	3	10	10	0	0	1	1	1	1	1	1	1	1	1	1	1	1	0.736	0.999	1
1	100	0.1	3	10	10	0	0	1	1	1	1	1	1	1	1	1	1	1	1	0.999	1	1
1	500	0.1	3	10	10	0	0	1	1	1	1	1	1	1	1	1	1	1	1	1	1	1
1	100	0.3	3	10	10	0	0	1	1	1	1	1	1	1	1	1	1	1	1	1	1	1
1	500	0.3	3	10	10	0	0	1	1	1	1	1	1	1	1	1	1	1	1	1	1	1
1	100	0	3	50	10	0	0	0.912	0.61	0.99	0.87	0.711	0.996	0.993	0.989	0.972	0.906	0.993	0.987	0.3	0.632	0.996
1	500	0	3	50	10	0	0	0.995	1	1	1	1	1	1	1	1	1	1	1	0.503	0.954	1
1	100	0.1	3	50	10	0	0	1	1	1	1	1	1	1	1	1	1	1	1	1	1	1
1	500	0.1	3	50	10	0	0	1	1	1	1	1	1	1	1	1	1	1	1	1	1	1
1	100	0.3	3	50	10	0	0	1	1	1	1	1	1	1	1	1	1	1	1	1	1	1
1	500	0.3	3	50	10	0	0	1	1	1	1	1	1	1	1	1	1	1	1	1	1	1
1	100	0	3	100	10	0	0	0.719	0.361	0.912	0.638	0.443	0.968	0.968	0.96	0.935	0.836	0.941	0.923	0.203	0.452	NA
1	500	0	3	100	10	0	0	0.962	0.561	1	0.999	0.999	1	1	1	1	1	1	1	0.415	0.87	1
1	100	0.1	3	100	10	0	0	1	1	1	1	1	1	1	1	1	1	1	1	1	1	NA
1	500	0.1	3	100	10	0	0	1	1	1	1	1	1	1	1	1	1	1	1	1	1	1
1	100	0.3	3	100	10	0	0	1	1	1	1	1	1	1	1	1	1	1	1	1	1	NA
1	500	0.3	3	100	10	0	0	1	1	1	1	1	1	1	1	1	1	1	1	1	1	1
1	100	0	3	500	10	0	0	0.316	0.134	0.425	0.232	0.155	0.617	0.659	0.696	0.698	0.596	0.471	0.379	0.108	0.187	NA
1	500	0	3	500	10	0	0	0.569	0.139	1	0.712	0.417	1	1	1	1	1	1	1	0.215	0.494	NA
1	100	0.1	3	500	10	0	0	1	1	1	1	1	1	1	1	1	1	1	0.953	1	1	NA
1	500	0.1	3	500	10	0	0	1	1	1	1	1	1	1	1	1	1	1	1	1	1	NA
1	100	0.3	3	500	10	0	0	1	1	1	1	1	1	1	1	1	1	1	1	1	1	NA
1	500	0.3	3	500	10	0	0	1	1	1	1	1	1	1	1	1	1	1	1	1	1	NA
1	100	0	6	10	10	0	0	0.916	0.835	0.943	0.914	0.88	0.902	0.853	0.796	0.719	0.63	0.938	0.876	0.343	0.753	0.949
1	500	0	6	10	10	0	0	1	1	1	1	1	1	1	1	1	1	1	1	0.602	0.982	1
1	100	0.1	6	10	10	0	0	1	1	0.999	1	1	0.999	0.999	0.999	0.995	0.989	0.999	0.999	0.998	0.999	0.999

1	500	0.1	6	10	10	0	0	1	1	1	1	1	1	1	1	1	1	1	1	1	1	1
1	100	0.3	6	10	10	0	0	1	1	1	1	1	1	1	1	1	1	1	1	1	1	1
1	500	0.3	6	10	10	0	0	1	1	1	1	1	1	1	1	1	1	1	1	1	1	1
1	100	0	6	50	10	0	0	0.498	0.287	0.576	0.427	0.321	0.613	0.606	0.564	0.464	0.356	0.604	0.531	0.173	0.322	0.45
1	500	0	6	50	10	0	0	0.989	0.931	1	0.998	0.976	1	1	1	1	1	1	1	0.34	0.802	1
1	100	0.1	6	50	10	0	0	1	0.998	1	1	0.999	1	1	1	0.998	0.968	1	0.956	1	0.993	0.805
1	500	0.1	6	50	10	0	0	1	1	1	1	1	1	1	1	1	1	1	1	1	1	1
1	100	0.3	6	50	10	0	0	1	1	1	1	1	1	1	1	1	1	1	1	1	1	0.994
1	500	0.3	6	50	10	0	0	1	1	1	1	1	1	1	1	1	1	1	1	1	1	1
1	100	0	6	100	10	0	0	0.338	0.186	0.394	0.284	0.199	0.434	0.434	0.41	0.356	0.265	0.429	0.353	0.104	0.215	NA
1	500	0	6	100	10	0	0	0.93	0.512	1	0.94	0.743	1	1	1	1	0.999	1	0.999	0.243	0.586	1
1	100	0.1	6	100	10	0	0	0.999	0.994	0.999	0.999	0.994	0.999	0.998	0.998	0.991	0.961	1	0.822	0.998	0.978	NA
1	500	0.1	6	100	10	0	0	1	1	1	1	1	1	1	1	1	1	1	1	1	1	1
1	100	0.3	6	100	10	0	0	1	1	1	1	1	1	1	1	1	1	1	1	1	1	NA
1	500	0.3	6	100	10	0	0	1	1	1	1	1	1	1	1	1	1	1	1	1	1	1
1	100	0	6	500	10	0	0	0.15	0.088	0.164	0.113	0.102	0.195	0.192	0.191	0.175	0.143	0.161	0.118	0.074	0.101	NA
1	500	0	6	500	10	0	0	0.493	0.134	0.833	0.359	0.201	0.986	0.995	0.998	0.998	0.997	0.907	0.867	0.124	0.241	NA
1	100	0.1	6	500	10	0	0	0.999	0.999	0.999	0.998	0.997	0.999	0.998	0.997	0.997	0.987	1	0.444	0.999	0.964	NA
1	500	0.1	6	500	10	0	0	1	1	1	1	1	1	1	1	1	1	1	1	1	1	NA
1	100	0.3	6	500	10	0	0	1	1	1	1	1	1	1	1	1	1	1	0.949	1	1	NA
1	500	0.3	6	500	10	0	0	1	1	1	1	1	1	1	1	1	1	1	1	1	1	NA
1	100	0	1	50	10	10	0	1	0.995	1	1	0.997	1	1	1	1	0.997	1	1	0.408	0.853	1
1	500	0	1	50	10	10	0	1	1	1	1	1	1	1	1	1	1	1	1	0.63	0.985	1
1	100	0.1	1	50	10	10	0	1	1	1	1	1	1	1	1	1	1	1	1	1	1	1
1	500	0.1	1	50	10	10	0	1	1	1	1	1	1	1	1	1	1	1	1	1	1	1
1	100	0.3	1	50	10	10	0	1	1	1	1	1	1	1	1	1	1	1	1	1	1	1
1	500	0.3	1	50	10	10	0	1	1	1	1	1	1	1	1	1	1	1	1	1	1	1
1	100	0	1	100	10	10	0	0.989	0.805	1	0.977	0.854	1	1	1	0.999	0.994	1	1	0.31	0.723	NA
1	500	0	1	100	10	10	0	1	1	1	1	1	1	1	1	1	1	1	1	0.539	0.956	1
1	100	0.1	1	100	10	10	0	1	1	1	1	1	1	1	1	1	1	1	1	1	1	NA
1	500	0.1	1	100	10	10	0	1	1	1	1	1	1	1	1	1	1	1	1	1	1	1
1	100	0.3	1	100	10	10	0	1	1	1	1	1	1	1	1	1	1	1	1	1	1	NA
1	500	0.3	1	100	10	10	0	1	1	1	1	1	1	1	1	1	1	1	1	1	1	1
1	100	0	1	500	10	10	0	0.629	0.239	0.828	0.464	0.278	0.968	0.981	0.989	0.992	0.952	0.888	0.832	0.157	0.347	NA
1	500	0	1	500	10	10	0	0.939	0.4	1	0.992	0.913	1	1	1	1	1	1	1	0.314	0.733	NA
1	100	0.1	1	500	10	10	0	1	1	1	1	1	1	1	1	1	1	1	1	1	1	NA
1	500	0.1	1	500	10	10	0	1	1	1	1	1	1	1	1	1	1	1	1	1	1	NA
1	100	0.3	1	500	10	10	0	1	1	1	1	1	1	1	1	1	1	1	1	1	1	NA
1	500	0.3	1	500	10	10	0	1	1	1	1	1	1	1	1	1	1	1	1	1	1	NA
1	100	0	3	50	10	10	0	1	0.981	1	0.998	0.986	1	1	1	1	0.979	1	1	0.379	0.812	1

1	500	0	3	50	10	10	0	1	1	1	1	1	1	1	1	1	1	1	1	0.611	0.974	1
1	100	0.1	3	50	10	10	0	1	1	1	1	1	1	1	1	1	1	1	1	1	1	1
1	500	0.1	3	50	10	10	0	1	1	1	1	1	1	1	1	1	1	1	1	1	1	1
1	100	0.3	3	50	10	10	0	1	1	1	1	1	1	1	1	1	1	1	1	1	1	1
1	500	0.3	3	50	10	10	0	1	1	1	1	1	1	1	1	1	1	1	1	1	1	1
1	100	0	3	100	10	10	0	0.976	0.751	1	0.944	0.793	1	1	0.999	0.997	0.965	1	0.998	0.28	0.643	NA
1	500	0	3	100	10	10	0	1	1	1	1	1	1	1	1	1	1	1	1	0.505	0.944	1
1	100	0.1	3	100	10	10	0	1	1	1	1	1	1	1	1	1	1	1	1	1	1	NA
1	500	0.1	3	100	10	10	0	1	1	1	1	1	1	1	1	1	1	1	1	1	1	1
1	100	0.3	3	100	10	10	0	1	1	1	1	1	1	1	1	1	1	1	1	1	1	NA
1	500	0.3	3	100	10	10	0	1	1	1	1	1	1	1	1	1	1	1	1	1	1	1
1	100	0	3	500	10	10	0	0.556	0.249	0.718	0.41	0.267	0.906	0.934	0.951	0.936	0.852	0.788	0.714	0.146	0.296	NA
1	500	0	3	500	10	10	0	0.935	0.4	1	0.978	0.852	1	1	1	1	1	1	1	0.275	0.678	NA
1	100	0.1	3	500	10	10	0	1	1	1	1	1	1	1	1	1	1	1	1	1	1	NA
1	500	0.1	3	500	10	10	0	1	1	1	1	1	1	1	1	1	1	1	1	1	1	NA
1	100	0.3	3	500	10	10	0	1	1	1	1	1	1	1	1	1	1	1	1	1	1	NA
1	500	0.3	3	500	10	10	0	1	1	1	1	1	1	1	1	1	1	1	1	1	1	NA
1	100	0	6	50	10	10	0	0.979	0.874	0.995	0.96	0.895	0.997	0.992	0.983	0.956	0.84	0.997	0.993	0.299	0.679	1
1	500	0	6	50	10	10	0	1	1	1	1	1	1	1	1	1	1	1	1	0.539	0.951	1
1	100	0.1	6	50	10	10	0	1	1	1	1	1	1	1	1	1	1	1	1	1	1	1
1	500	0.1	6	50	10	10	0	1	1	1	1	1	1	1	1	1	1	1	1	1	1	1
1	100	0.3	6	50	10	10	0	1	1	1	1	1	1	1	1	1	1	1	1	1	1	1
1	500	0.3	6	50	10	10	0	1	1	1	1	1	1	1	1	1	1	1	1	1	1	1
1	100	0	6	100	10	10	0	0.878	0.537	0.959	0.787	0.579	0.98	0.976	0.961	0.917	0.759	0.967	0.952	0.221	0.491	NA
1	500	0	6	100	10	10	0	1	0.999	1	1	1	1	1	1	1	1	1	1	0.414	0.878	1
1	100	0.1	6	100	10	10	0	1	1	1	1	1	1	1	1	1	1	1	1	1	1	NA
1	500	0.1	6	100	10	10	0	1	1	1	1	1	1	1	1	1	1	1	1	1	1	1
1	100	0.3	6	100	10	10	0	1	1	1	1	1	1	1	1	1	1	1	1	1	1	NA
1	500	0.3	6	100	10	10	0	1	1	1	1	1	1	1	1	1	1	1	1	1	1	1
1	100	0	6	500	10	10	0	0.405	0.184	0.481	0.308	0.215	0.638	0.66	0.688	0.658	0.507	0.524	0.418	0.107	0.207	NA
1	500	0	6	500	10	10	0	0.897	0.344	1	0.905	0.674	1	1	1	1	1	1	1	0.21	0.53	NA
1	100	0.1	6	500	10	10	0	1	1	1	1	1	1	1	1	1	1	1	0.998	1	1	NA
1	500	0.1	6	500	10	10	0	1	1	1	1	1	1	1	1	1	1	1	1	1	1	NA
1	100	0.3	6	500	10	10	0	1	1	1	1	1	1	1	1	1	1	1	1	1	1	NA
1	500	0.3	6	500	10	10	0	1	1	1	1	1	1	1	1	1	1	1	1	1	1	NA
1	100	0	1	50	25	0	0	1	1	1	1	1	1	1	1	0.999	0.971	1	1	0.412	0.849	1
1	500	0	1	50	25	0	0	1	1	1	1	1	1	1	1	1	1	1	1	0.625	0.986	1
1	100	0.1	1	50	25	0	0	1	1	1	1	1	1	1	1	1	1	1	1	1	1	1
1	500	0.1	1	50	25	0	0	1	1	1	1	1	1	1	1	1	1	1	1	1	1	1
1	100	0.3	1	50	25	0	0	1	1	1	1	1	1	1	1	1	1	1	1	1	1	1

1	500	0.3	1	50	25	0	0	1	1	1	1	1	1	1	1	1	1	1	1	1	1	1
1	100	0	1	100	25	0	0	0.998	0.959	1	0.996	0.951	1	1	0.999	0.992	0.937	1	0.999	0.301	0.699	NA
1	500	0	1	100	25	0	0	1	1	1	1	1	1	1	1	1	1	1	1	0.546	0.951	1
1	100	0.1	1	100	25	0	0	1	1	1	1	1	1	1	1	1	1	1	1	1	1	NA
1	500	0.1	1	100	25	0	0	1	1	1	1	1	1	1	1	1	1	1	1	1	1	1
1	100	0.3	1	100	25	0	0	1	1	1	1	1	1	1	1	1	1	1	1	1	1	NA
1	500	0.3	1	100	25	0	0	1	1	1	1	1	1	1	1	1	1	1	1	1	1	1
1	100	0	1	500	25	0	0	0.732	0.33	0.847	0.533	0.357	0.945	0.956	0.957	0.921	0.753	0.888	0.828	0.141	0.325	NA
1	500	0	1	500	25	0	0	0.995	0.724	1	0.998	0.964	1	1	1	1	1	1	1	0.318	0.716	NA
1	100	0.1	1	500	25	0	0	1	1	1	1	1	1	1	1	1	1	1	1	1	1	NA
1	500	0.1	1	500	25	0	0	1	1	1	1	1	1	1	1	1	1	1	1	1	1	NA
1	100	0.3	1	500	25	0	0	1	1	1	1	1	1	1	1	1	1	1	1	1	1	NA
1	500	0.3	1	500	25	0	0	1	1	1	1	1	1	1	1	1	1	1	1	1	1	NA
1	100	0	3	50	25	0	0	0.999	0.993	1	0.999	0.995	1	1	0.997	0.979	0.863	1	1	0.349	0.772	1
1	500	0	3	50	25	0	0	1	1	1	1	1	1	1	1	1	1	1	1	0.557	0.968	1
1	100	0.1	3	50	25	0	0	1	1	1	1	1	1	1	1	1	1	1	1	1	1	1
1	500	0.1	3	50	25	0	0	1	1	1	1	1	1	1	1	1	1	1	1	1	1	1
1	100	0.3	3	50	25	0	0	1	1	1	1	1	1	1	1	1	1	1	1	1	1	1
1	500	0.3	3	50	25	0	0	1	1	1	1	1	1	1	1	1	1	1	1	1	1	1
1	100	0	3	100	25	0	0	0.984	0.868	0.996	0.948	0.851	0.995	0.991	0.983	0.95	0.774	0.996	0.993	0.255	0.574	NA
1	500	0	3	100	25	0	0	1	1	1	1	1	1	1	1	1	1	1	1	0.497	0.918	1
1	100	0.1	3	100	25	0	0	1	1	1	1	1	1	1	1	1	1	1	1	1	1	NA
1	500	0.1	3	100	25	0	0	1	1	1	1	1	1	1	1	1	1	1	1	1	1	1
1	100	0.3	3	100	25	0	0	1	1	1	1	1	1	1	1	1	1	1	1	1	1	NA
1	500	0.3	3	100	25	0	0	1	1	1	1	1	1	1	1	1	1	1	1	1	1	1
1	100	0	3	500	25	0	0	0.574	0.269	0.656	0.425	0.307	0.786	0.786	0.763	0.702	0.495	0.702	0.594	0.131	0.268	NA
1	500	0	3	500	25	0	0	0.991	0.697	1	0.992	0.881	1	1	1	1	1	1	1	0.255	0.626	NA
1	100	0.1	3	500	25	0	0	1	1	1	1	1	1	1	1	1	1	1	1	1	1	NA
1	500	0.1	3	500	25	0	0	1	1	1	1	1	1	1	1	1	1	1	1	1	1	NA
1	100	0.3	3	500	25	0	0	1	1	1	1	1	1	1	1	1	1	1	1	1	1	NA
1	500	0.3	3	500	25	0	0	1	1	1	1	1	1	1	1	1	1	1	1	1	1	NA
1	100	0	6	50	25	0	0	0.924	0.771	0.94	0.879	0.796	0.909	0.882	0.829	0.7	0.451	0.939	0.909	0.247	0.55	0.917
1	500	0	6	50	25	0	0	1	1	1	1	1	1	1	1	1	1	1	1	0.457	0.916	1
1	100	0.1	6	50	25	0	0	1	1	1	1	1	1	1	1	1	1	1	1	1	1	1
1	500	0.1	6	50	25	0	0	1	1	1	1	1	1	1	1	1	1	1	1	1	1	1
1	100	0.3	6	50	25	0	0	1	1	1	1	1	1	1	1	1	1	1	1	1	1	1
1	500	0.3	6	50	25	0	0	1	1	1	1	1	1	1	1	1	1	1	1	1	1	1
1	100	0	6	100	25	0	0	0.739	0.493	0.791	0.652	0.511	0.794	0.772	0.713	0.589	0.375	0.805	0.756	0.19	0.375	NA
1	500	0	6	100	25	0	0	1	1	1	1	1	1	1	1	1	1	1	1	0.364	0.772	1
1	100	0.1	6	100	25	0	0	1	1	1	1	1	1	1	1	1	1	1	1	1	1	NA

1	500	0.1	6	100	25	0	0	1	1	1	1	1	1	1	1	1	1	1	1	1	1	1
1	100	0.3	6	100	25	0	0	1	1	1	1	1	1	1	1	1	1	1	1	1	1	NA
1	500	0.3	6	100	25	0	0	1	1	1	1	1	1	1	1	1	1	1	1	1	1	1
1	100	0	6	500	25	0	0	0.309	0.175	0.337	0.24	0.165	0.357	0.352	0.334	0.28	0.176	0.358	0.248	0.094	0.156	NA
1	500	0	6	500	25	0	0	0.958	0.539	0.999	0.884	0.628	1	1	1	1	1	1	1	0.18	0.398	NA
1	100	0.1	6	500	25	0	0	1	1	1	1	1	1	1	1	1	1	1	0.996	1	1	NA
1	500	0.1	6	500	25	0	0	1	1	1	1	1	1	1	1	1	1	1	1	1	1	NA
1	100	0.3	6	500	25	0	0	1	1	1	1	1	1	1	1	1	1	1	1	1	1	NA
1	500	0.3	6	500	25	0	0	1	1	1	1	1	1	1	1	1	1	1	1	1	1	NA
1	100	0	1	50	25	25	0	1	1	1	1	1	1	1	1	0.992	0.915	1	1	0.41	0.853	1
1	500	0	1	50	25	25	0	1	1	1	1	1	1	1	1	1	1	1	1	0.609	0.984	1
1	100	0.1	1	50	25	25	0	1	1	1	1	1	1	1	1	1	1	1	1	1	1	1
1	500	0.1	1	50	25	25	0	1	1	1	1	1	1	1	1	1	1	1	1	1	1	1
1	100	0.3	1	50	25	25	0	1	1	1	1	1	1	1	1	1	1	1	1	1	1	1
1	500	0.3	1	50	25	25	0	1	1	1	1	1	1	1	1	1	1	1	1	1	1	1
1	100	0	1	100	25	25	0	1	0.991	1	0.998	0.985	1	1	1	0.987	0.838	1	1	0.29	0.689	NA
1	500	0	1	100	25	25	0	1	1	1	1	1	1	1	1	1	1	1	1	0.55	0.96	1
1	100	0.1	1	100	25	25	0	1	1	1	1	1	1	1	1	1	1	1	1	1	1	NA
1	500	0.1	1	100	25	25	0	1	1	1	1	1	1	1	1	1	1	1	1	1	1	1
1	100	0.3	1	100	25	25	0	1	1	1	1	1	1	1	1	1	1	1	1	1	1	NA
1	500	0.3	1	100	25	25	0	1	1	1	1	1	1	1	1	1	1	1	1	1	1	1
1	100	0	1	500	25	25	0	0.786	0.422	0.852	0.636	0.464	0.92	0.915	0.897	0.837	0.577	0.874	0.868	0.181	0.323	NA
1	500	0	1	500	25	25	0	1	1	1	1	0.994	1	1	1	1	1	1	1	0.33	0.72	NA
1	100	0.1	1	500	25	25	0	1	1	1	1	1	1	1	1	1	1	1	1	1	1	NA
1	500	0.1	1	500	25	25	0	1	1	1	1	1	1	1	1	1	1	1	1	1	1	NA
1	100	0.3	1	500	25	25	0	1	1	1	1	1	1	1	1	1	1	1	1	1	1	NA
1	500	0.3	1	500	25	25	0	1	1	1	1	1	1	1	1	1	1	1	1	1	1	NA
1	100	0	3	50	25	25	0	1	1	1	1	1	1	1	1	0.989	0.889	1	1	0.394	0.85	1
1	500	0	3	50	25	25	0	1	1	1	1	1	1	1	1	1	1	1	1	0.612	0.982	1
1	100	0.1	3	50	25	25	0	1	1	1	1	1	1	1	1	1	1	1	1	1	1	1
1	500	0.1	3	50	25	25	0	1	1	1	1	1	1	1	1	1	1	1	1	1	1	1
1	100	0.3	3	50	25	25	0	1	1	1	1	1	1	1	1	1	1	1	1	1	1	1
1	500	0.3	3	50	25	25	0	1	1	1	1	1	1	1	1	1	1	1	1	1	1	1
1	100	0	3	100	25	25	0	1	0.98	1	0.997	0.968	1	1	0.999	0.974	0.804	1	1	0.279	0.655	NA
1	500	0	3	100	25	25	0	1	1	1	1	1	1	1	1	1	1	1	1	0.534	0.951	1
1	100	0.1	3	100	25	25	0	1	1	1	1	1	1	1	1	1	1	1	1	1	1	NA
1	500	0.1	3	100	25	25	0	1	1	1	1	1	1	1	1	1	1	1	1	1	1	1
1	100	0.3	3	100	25	25	0	1	1	1	1	1	1	1	1	1	1	1	1	1	1	NA
1	500	0.3	3	100	25	25	0	1	1	1	1	1	1	1	1	1	1	1	1	1	1	1
1	100	0	3	500	25	25	0	0.755	0.408	0.816	0.581	0.415	0.881	0.881	0.862	0.777	0.543	0.844	0.817	0.171	0.318	NA

1	500	0	3	500	25	25	0	1	1	1	1	0.995	1	1	1	1	1	1	1	0.324	0.706	NA
1	100	0.1	3	500	25	25	0	1	1	1	1	1	1	1	1	1	1	1	1	1	1	NA
1	500	0.1	3	500	25	25	0	1	1	1	1	1	1	1	1	1	1	1	1	1	1	NA
1	100	0.3	3	500	25	25	0	1	1	1	1	1	1	1	1	1	1	1	1	1	1	NA
1	500	0.3	3	500	25	25	0	1	1	1	1	1	1	1	1	1	1	1	1	1	1	NA
1	100	0	6	50	25	25	0	1	0.998	1	1	0.997	1	1	0.991	0.962	0.795	1	1	0.365	0.781	1
1	500	0	6	50	25	25	0	1	1	1	1	1	1	1	1	1	1	1	1	0.584	0.973	1
1	100	0.1	6	50	25	25	0	1	1	1	1	1	1	1	1	1	1	1	1	1	1	1
1	500	0.1	6	50	25	25	0	1	1	1	1	1	1	1	1	1	1	1	1	1	1	1
1	100	0.3	6	50	25	25	0	1	1	1	1	1	1	1	1	1	1	1	1	1	1	1
1	500	0.3	6	50	25	25	0	1	1	1	1	1	1	1	1	1	1	1	1	1	1	1
1	100	0	6	100	25	25	0	0.997	0.928	1	0.977	0.923	0.997	0.992	0.979	0.918	0.684	1	0.998	0.255	0.573	NA
1	500	0	6	100	25	25	0	1	1	1	1	1	1	1	1	1	1	1	1	0.492	0.924	1
1	100	0.1	6	100	25	25	0	1	1	1	1	1	1	1	1	1	1	1	1	1	1	NA
1	500	0.1	6	100	25	25	0	1	1	1	1	1	1	1	1	1	1	1	1	1	1	1
1	100	0.3	6	100	25	25	0	1	1	1	1	1	1	1	1	1	1	1	1	1	1	NA
1	500	0.3	6	100	25	25	0	1	1	1	1	1	1	1	1	1	1	1	1	1	1	1
1	100	0	6	500	25	25	0	0.629	0.34	0.683	0.477	0.343	0.76	0.754	0.719	0.639	0.417	0.719	0.656	0.147	0.26	NA
1	500	0	6	500	25	25	0	1	0.994	1	1	0.987	1	1	1	1	1	1	1	0.276	0.631	NA
1	100	0.1	6	500	25	25	0	1	1	1	1	1	1	1	1	1	1	1	1	1	1	NA
1	500	0.1	6	500	25	25	0	1	1	1	1	1	1	1	1	1	1	1	1	1	1	NA
1	100	0.3	6	500	25	25	0	1	1	1	1	1	1	1	1	1	1	1	1	1	1	NA
1	500	0.3	6	500	25	25	0	1	1	1	1	1	1	1	1	1	1	1	1	1	1	NA
1	100	0	1	50	50	0	0	1	1	1	1	1	1	1	1	0.992	0.85	1	1	0.42	0.848	1
1	500	0	1	50	50	0	0	1	1	1	1	1	1	1	1	1	1	1	1	0.618	0.982	1
1	100	0.1	1	50	50	0	0	1	1	1	1	1	1	1	1	1	1	1	1	1	1	1
1	500	0.1	1	50	50	0	0	1	1	1	1	1	1	1	1	1	1	1	1	1	1	1
1	100	0.3	1	50	50	0	0	1	1	1	1	1	1	1	1	1	1	1	1	1	1	1
1	500	0.3	1	50	50	0	0	1	1	1	1	1	1	1	1	1	1	1	1	1	1	1
1	100	0	1	100	50	0	0	1	0.994	1	0.999	0.995	1	1	0.996	0.974	0.769	1	1	0.291	0.686	NA
1	500	0	1	100	50	0	0	1	1	1	1	1	1	1	1	1	1	1	1	0.546	0.958	1
1	100	0.1	1	100	50	0	0	1	1	1	1	1	1	1	1	1	1	1	1	1	1	NA
1	500	0.1	1	100	50	0	0	1	1	1	1	1	1	1	1	1	1	1	1	1	1	1
1	100	0.3	1	100	50	0	0	1	1	1	1	1	1	1	1	1	1	1	1	1	1	NA
1	500	0.3	1	100	50	0	0	1	1	1	1	1	1	1	1	1	1	1	1	1	1	1
1	100	0	1	500	50	0	0	0.804	0.496	0.851	0.674	0.48	0.908	0.9	0.875	0.748	0.484	0.883	0.855	0.169	0.335	NA
1	500	0	1	500	50	0	0	1	1	1	1	1	1	1	1	1	1	1	1	0.323	0.7	NA
1	100	0.1	1	500	50	0	0	1	1	1	1	1	1	1	1	1	1	1	1	1	1	NA
1	500	0.1	1	500	50	0	0	1	1	1	1	1	1	1	1	1	1	1	1	1	1	NA
1	100	0.3	1	500	50	0	0	1	1	1	1	1	1	1	1	1	1	1	1	1	1	NA

1	500	0.3	1	500	50	0	0	1	1	1	1	1	1	1	1	1	1	1	1	1	1	NA
1	100	0	3	50	50	0	0	1	1	1	1	0.999	1	1	0.995	0.966	0.776	1	1	0.384	0.802	1
1	500	0	3	50	50	0	0	1	1	1	1	1	1	1	1	1	1	1	1	0.59	0.978	1
1	100	0.1	3	50	50	0	0	1	1	1	1	1	1	1	1	1	1	1	1	1	1	1
1	500	0.1	3	50	50	0	0	1	1	1	1	1	1	1	1	1	1	1	1	1	1	1
1	100	0.3	3	50	50	0	0	1	1	1	1	1	1	1	1	1	1	1	1	1	1	1
1	500	0.3	3	50	50	0	0	1	1	1	1	1	1	1	1	1	1	1	1	1	1	1
1	100	0	3	100	50	0	0	1	0.98	1	0.998	0.975	0.999	0.996	0.989	0.931	0.664	1	1	0.252	0.626	NA
1	500	0	3	100	50	0	0	1	1	1	1	1	1	1	1	1	1	1	1	0.514	0.942	1
1	100	0.1	3	100	50	0	0	1	1	1	1	1	1	1	1	1	1	1	1	1	1	NA
1	500	0.1	3	100	50	0	0	1	1	1	1	1	1	1	1	1	1	1	1	1	1	1
1	100	0.3	3	100	50	0	0	1	1	1	1	1	1	1	1	1	1	1	1	1	1	NA
1	500	0.3	3	100	50	0	0	1	1	1	1	1	1	1	1	1	1	1	1	1	1	1
1	100	0	3	500	50	0	0	0.707	0.41	0.754	0.572	0.426	0.801	0.79	0.745	0.636	0.385	0.774	0.726	0.146	0.299	NA
1	500	0	3	500	50	0	0	1	1	1	1	0.996	1	1	1	1	1	1	1	0.286	0.659	NA
1	100	0.1	3	500	50	0	0	1	1	1	1	1	1	1	1	1	1	1	1	1	1	NA
1	500	0.1	3	500	50	0	0	1	1	1	1	1	1	1	1	1	1	1	1	1	1	NA
1	100	0.3	3	500	50	0	0	1	1	1	1	1	1	1	1	1	1	1	1	1	1	NA
1	500	0.3	3	500	50	0	0	1	1	1	1	1	1	1	1	1	1	1	1	1	1	NA
1	100	0	6	50	50	0	0	0.996	0.976	0.998	0.992	0.973	0.989	0.977	0.922	0.807	0.527	0.997	0.99	0.314	0.673	1
1	500	0	6	50	50	0	0	1	1	1	1	1	1	1	1	1	1	1	1	0.525	0.952	1
1	100	0.1	6	50	50	0	0	1	1	1	1	1	1	1	1	1	1	1	1	1	1	1
1	500	0.1	6	50	50	0	0	1	1	1	1	1	1	1	1	1	1	1	1	1	1	1
1	100	0.3	6	50	50	0	0	1	1	1	1	1	1	1	1	1	1	1	1	1	1	1
1	500	0.3	6	50	50	0	0	1	1	1	1	1	1	1	1	1	1	1	1	1	1	1
1	100	0	6	100	50	0	0	0.955	0.819	0.968	0.902	0.802	0.939	0.904	0.847	0.697	0.433	0.97	0.95	0.219	0.479	NA
1	500	0	6	100	50	0	0	1	1	1	1	1	1	1	1	1	1	1	1	0.428	0.89	1
1	100	0.1	6	100	50	0	0	1	1	1	1	1	1	1	1	1	1	1	1	1	1	NA
1	500	0.1	6	100	50	0	0	1	1	1	1	1	1	1	1	1	1	1	1	1	1	1
1	100	0.3	6	100	50	0	0	1	1	1	1	1	1	1	1	1	1	1	1	1	1	NA
1	500	0.3	6	100	50	0	0	1	1	1	1	1	1	1	1	1	1	1	1	1	1	1
1	100	0	6	500	50	0	0	0.476	0.285	0.502	0.379	0.283	0.532	0.489	0.451	0.356	0.217	0.521	0.413	0.116	0.191	NA
1	500	0	6	500	50	0	0	1	0.986	1	0.999	0.969	1	1	1	1	1	1	1	0.215	0.518	NA
1	100	0.1	6	500	50	0	0	1	1	1	1	1	1	1	1	1	1	1	1	1	1	NA
1	500	0.1	6	500	50	0	0	1	1	1	1	1	1	1	1	1	1	1	1	1	1	NA
1	100	0.3	6	500	50	0	0	1	1	1	1	1	1	1	1	1	1	1	1	1	1	NA
1	500	0.3	6	500	50	0	0	1	1	1	1	1	1	1	1	1	1	1	1	1	1	NA
